# Dianthiamides A–E, Proline-Containing Orbitides from *Dianthus chinensis*

**DOI:** 10.3390/molecules26237275

**Published:** 2021-11-30

**Authors:** Jin Woo Lee, Jun Gu Kim, Jae Sang Han, Yong Beom Cho, Yu Jin Lee, Dongho Lee, Dae Hwan Shin, Jin Tae Hong, Mi Kyeong Lee, Bang Yeon Hwang

**Affiliations:** 1 College of Pharmacy, Chungbuk National University, Cheongju 28160, Korea; jinu15@hanmail.net (J.W.L.); sossgi@naver.com (J.G.K.); han00768@hanmail.net (J.S.H.); bum94@naver.com (Y.B.C.); yujinli@naver.com (Y.J.L.); dshin@chungbuk.ac.kr (D.H.S.); jinthong@chungbuk.ac.kr (J.T.H.); mklee@chungbuk.ac.kr (M.K.L.); 2 Department of Plant Biotechnology, College of Life Sciences and Biotechnology, Korea University, Seoul 02841, Korea; dongholee@korea.ac.kr

**Keywords:** *Dianthus chinensis*, Caryophyllaceae, orbitide, structure elucidation, cytotoxic activity

## Abstract

Orbitides are plant-derived small cyclic peptides with a wide range of biological activities. Phytochemical investigation of the whole plants of *Dianthus chinensis* was performed with the aim to discover new bioactive orbitides. Five undescribed proline-containing orbitides, dianthiamides A–E (**1**–**5**), were isolated from a methanolic extract of *Dianthus chinensis*. Their structures were elucidated by extensive analysis of 1D and 2D NMR and HRESI–TOF–MS as well as ESI–MS/MS fragmentation data. The absolute configuration of the amino acid residues of compounds **1**–**5** was determined by Marfey’s method. All compounds were tested for their cytotoxic activity, and dianthiamide A (**1**) exhibited weak activity against A549 cell line with IC_50_ value of 47.9 μM.

## 1. Introduction

**Figure 1:**
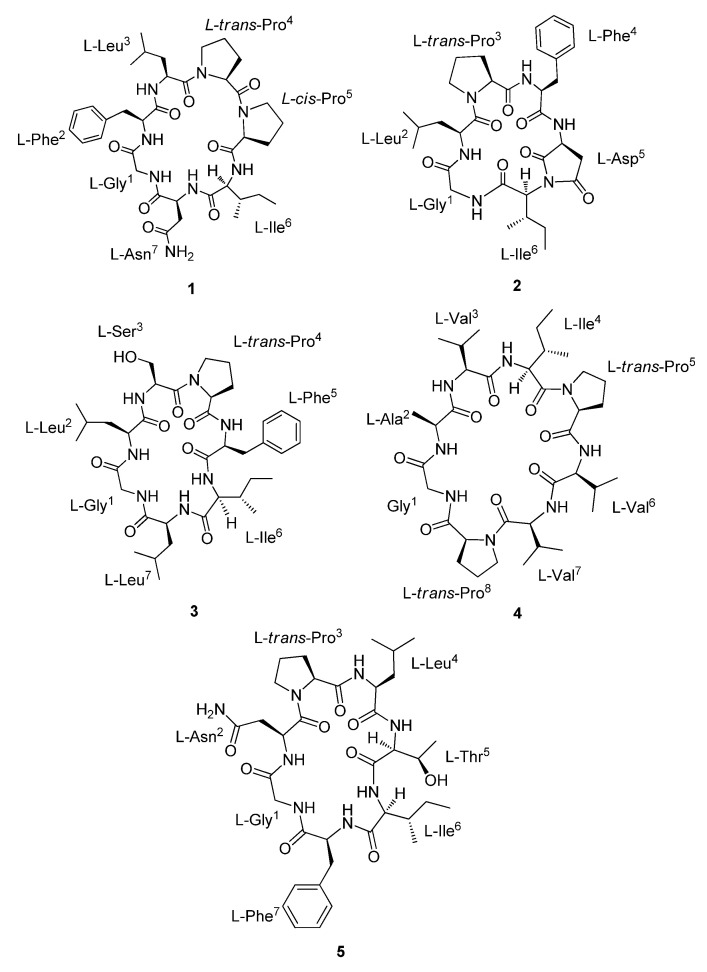
Structures of compounds **1**–**5**.

*Dianthus chinensis* L. is a perennial herbaceous plant belonging to the Caryophyllaceae family, and is distributed widely in Europe and Eastern Asia. The whole plant of *D. chinensis* is commonly used as a traditional medicine in Korea for treating diuretic, carcinoma, urethritis, and carbuncles [1,2,3,4,5,6]. Previous investigation on the phytochemical constituents of the genus *Dianthus* led to the isolation of cyclopeptides [4,5,6,7,8,9], dianthramides [5,10,11], triterpenoidal saponins [1,2,3,12,13], anthocyanins [14], and pyran-type glycosides [15]. Orbitides, formerly known as Caryophyllaceae-type cyclic peptides, are N-to-C cyclized plant peptides lacking disulfide bonds, which possess 5 to 12 amino acid residues. Orbitides are ribosomally synthesized and post-translationally modified cyclic peptides, which have been discovered in many plants of the families such as Annonaceae, Asteraceae, Caryophyllaceae, Euphorbiaceae, Lamiaceae, Linaceae, Phytolaccaceae, Rutaceae, Schizandraceae, and Verbenaceae. Recently, orbitides have gained increasing attention owed to a wide range of biological activities including cytotoxic, antimalarial, immunomodulatory, and antiproliferative activities [16,17,18,19]. The *Dianthus* genus is a rich source of proline-containing orbitides, some of which showed cytotoxic activity against several cancer cell lines [5,6,18,20]. Therefore, we have embarked on a research program for the isolation of new bioactive orbitides from medicinal plant, and five undescribed orbitides, dianthiamides A–E (**1**–**5**) (Figure 1), were isolated from a MeOH extract of the whole plants of the *D. chinensis*. Herein, the isolation and structure determination as well as their cytotoxic activity against A549 cell line are described.

## 2. Results and Discussion

Dianthiamide A (**1**) was obtained as a yellow amorphous powder. Its molecular formula of C_37_H_54_N_8_O_8_ was determined from the HRESI–TOF–MS data (*m*/*z* 739.4144 [M + H]^+^; calcd for 739.4137). The ^1^H and ^13^C NMR in conjunction with HSQC data of **1** displayed the presence of 37 carbon signals assigned to eight amide carbonyl carbons (δ_C_ 168.8, 169.8, 170.8, 170.9, 171.1, 171.4, 172.3, and 173.4), seven α-amino acid carbons [δ_C_ 44.4 (CH_2_), 48.6 (CH), 49.6 (CH), 56.0 (CH), 59.6 (CH), 60.8 (CH), and 61.6 (CH)], six aromatic carbons [δ_C_ 126.8, 128.8, (2C), 129.1 (2C), and 138.4], two methines, ten methylenes, and four methyls (Table 1), suggesting that **1** is a heptapeptide. Furthermore, HSQC, HMBC, and COSY spectra showed the identification of seven amino acid residues including phenylalanine (F), glycine (G), isoleucine (I), asparagine (N), leucine (L), and two prolines (P_a_ and P_b_).

**Figure 2:**
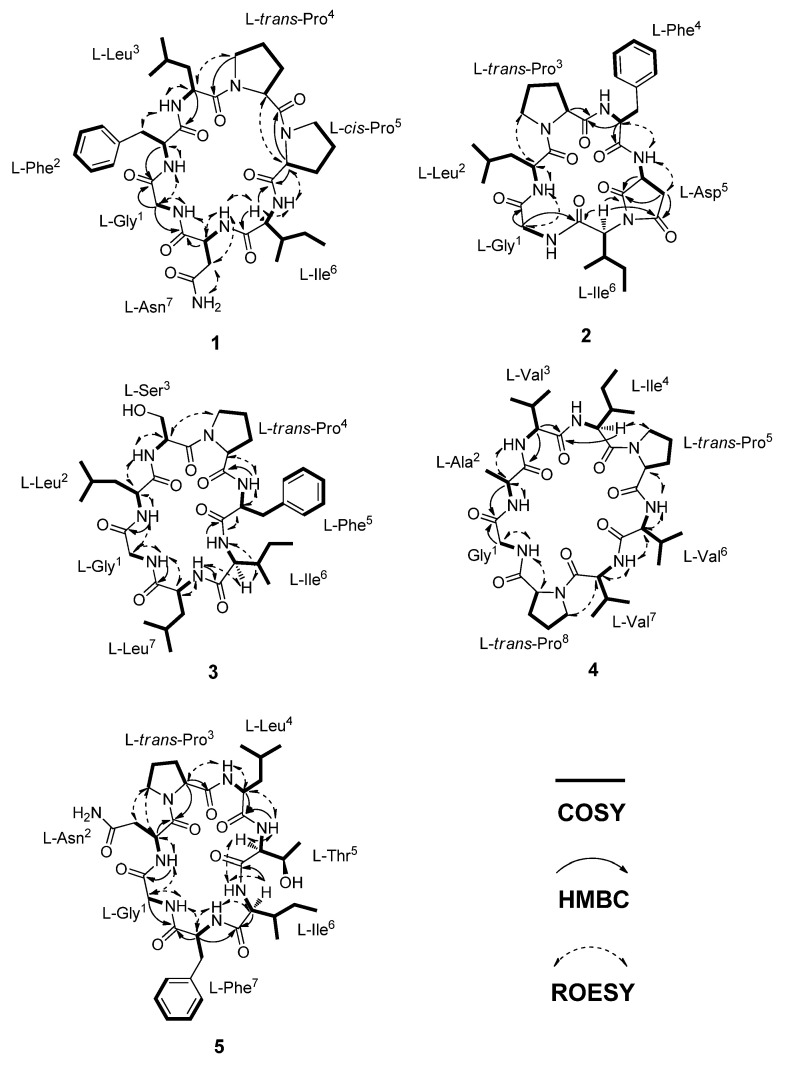
Key COSY, HMBC, and ROESY correlations of **1–5**.

In the HMBC and ROESY experiments, the cyclic feature and amino acid sequence of **1** were elucidated by the correlations observed between the amino acid Hα and continuous amide group (CONH). Therefore, the linear sequence of **1** was identified as G-F-L-P_a_-P_b_-I-N. Also, the HMBC correlation from Gly-Hα (δ_H_ 3.40 and 3.55) to Asn-C=O (δ_C_ 172.3) as well as the ROESY correlation between Gly-NH (δ_H_ 8.70) and Asn-Hα (δ_H_ 4.18) established the cyclic heptapeptide as *cyclo*-G-F-L-P_a_-P_b_-I-N (Figure 2). The amino acid sequence of **1** was further confirmed by analysis of the ESI–MS/MS fragment ions. Presumably, though there were several ring-opening sites, it occurred at two preferred positions at Pro_a_^4^-Pro_b_^5^ and Ile^6^-Asn^7^, respectively. Each the linear sequences, Pro_b_^5^-Ile^6^-Asn^7^-Gly^1^-Phe^2^-Leu^3^-Pro_a_^4^ (b_7_P_b_P_a_) and Asn^7^-Gly^1^-Phe^2^-Leu^3^-Pro_a_^4^-Pro_b_^5^-Ile^6^ (b_7_NI) was certified by acylium ions (b_n_P_b_P_a_ and b_n_NI) and after loss of CO (a_n_NI) at *m*/*z* 642 (b_6_P_b_P_a_), 626 (b_6_NI), 529 (b_5_P_b_P_a_ and b_5_NI), 404 (a_4_NI), 364 (b_4_P_b_P_a_-H_2_O), 319 (b_3_NI), and 211 (b_2_P_b_P_a_), corresponding to the successive loss of amino acid residues (Figure S9).

**Figure 3:**
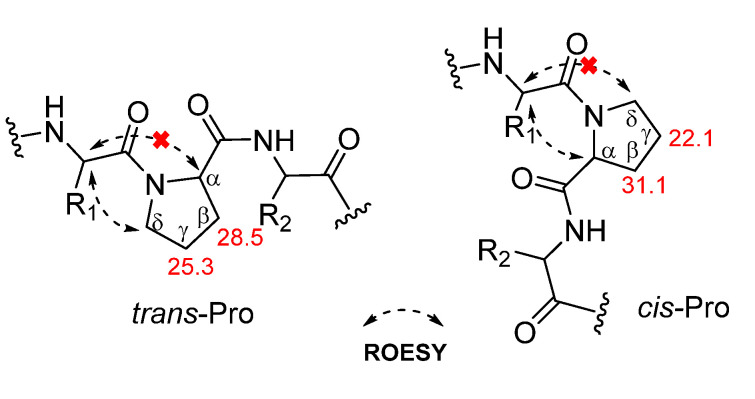
Proline isomerization of **1**.

The geometry of proline residues was assigned on the basis of the Δ*δ*_Cβ-Cγ_ values and the presence of a ROE correlations between the proline Hα or Hδ and the Hα of previous amino acid. The Δ*δ*_Cβ-Cγ_ value (3.2 ppm) of the Pro_a_^4^ and the ROE correlation between the Hα (δ_H_ 4.48) of Leu^3^ and the Hδ (δ_H_ 3.44) of Pro_a_^4^ indicated that the amide bond in the Pro_a_^4^ was a *trans*. However, the Δ*δ*_Cβ-Cγ_ value (9.0 ppm) of the Pro_b_^5^ and the ROE correlation between the Hα (δ_H_ 4.45) of Pro_a_^4^ and the Hα (δ_H_ 4.55) of Pro_b_^5^ indicated that the geometry of Pro_b_^5^ was a *cis* (Figure 3) [21,22,23]. The absolute configuration of amino acid residues in **1** were identified as L configuration, which was deduced by acid hydrolysis and Marfey’s derivatization, followed by HPLC analysis [24,25,26,27]. The *N*-*α*-(2,4-dinitro-5-fluorophenyl)-l-alaninamide (l-FDAA)-derivatives of **1** gave peaks at *t*_R_ (min) 15.0 (l-Asp, *m*/*z* 386), 20.8 (l-Pro, *m*/*z* 368), 29.5 (l-Leu, *m*/*z* 384), 29.7 (l-Phe, *m*/*z* 418), and 30.2 (l-Ile, *m*/*z* 384) (Figure S10). Therefore, dianthiamide A (**1**) was established as *cyclo*-(Gly^1^-l-Phe^2^-l-Leu^3^-l-*trans*-Pro_a_^4^-l-*cis*-Pro_b_^5^-l-Ile^6^-l-Asn^7^).

Dianthiamide B (**2**) was isolated as a yellow amorphous powder, the HRESI–TOF–MS data were consistent with the molecular formula C_32_H_44_N_6_O_7_ (*m*/*z* 647.3161 [M + Na]^+^; calcd for 647.3163). The ^1^H, ^13^C and HSQC NMR spectra of **2** showed 32 carbons, consisting of seven amide carbonyl carbons, six α-amino acid carbons, six aromatic carbons, two methines, seven methylenes, and four methyls (Table 2). HSQC, HMBC, and COSY spectra demonstrated the presence of six amino acid residues including aspartic acid (D), phenylalanine (F), glycine (G), isoleucine (I), leucine (L), and proline (P). Moreover, HMBC, COSY, and ROESY spectra indicated that the sequence and connectivity of the hexapeptide was *cyclo*-G-L-P-F-D-I (Figure 2). The HMBC correlations between Hα (δ_H_ 4.19) of Ile^6^ and two carbonyls (δ_C_ 176.2 and 177.2) of Asp^5^ showed that dehydration of NH-Ile^6^ and COOH-Asp^5^ formed an additional five membered ring system (pyrrolidine-2,5-dione). The amino acid sequence in **2** was further supported by the fragmentation pattern of ESI–MS/MS data, in which the preferred ring-opening of **2** occurred at the amide bond between leucine and proline (Figure S19). The geometry of amide bond of Pro^3^ residue in **2** was assigned the *trans* configuration, on the basis of the difference of the ^13^C NMR chemical shift (Δ*δ*_Cβ-Cγ_ = 3.9 ppm) [21,22,23] as well as the ROE correlation between the Hα (δ_H_ 4.51) of Leu^2^ and the Hδ (δ_H_ 3.68 and 3.42) of Pro^3^ residue. In addition, the absolute configuration of six amino acid residues in **2** were all assigned as L, which was determined by HPLC analysis of the acid hydrolysate after Marfey’s derivatization (Figure S20). Therefore, dianthiamide B (**2**) was determined as *cyclo*-(Gly^1^-l-Leu^2^-l-*trans*-Pro^3^-l-Phe^4^-l-Asp^5^-l-Ile^6^).

Dianthiamide C (**3**) was obtained as a yellow amorphous powder, showed a molecular formula of C_37_H_57_N_7_O_8_ as determined by its HRESI–TOF–MS data (*m*/*z* 750.4165 [M + Na]^+^; calcd 750.4160). The ^13^C and HSQC NMR data of **3** displayed the presence of 37 carbon signals including seven amide carbonyls, as well as seven α-amino acid carbons, suggesting a heptapeptide (Table 3). Full assignments of ^1^H and ^13^C NMR data for each amino acid residue were accomplished by combined analysis of COSY, HSQC, and HMBC spectra and suggested that **3** was composed of seven amino acid such as phenylalanine (F), glycine (G), isoleucine (I), two leucines (L_a_ and L_b_), proline (P), and serine (S) residue (Table 3). The HMBC and ROESY spectra indicated that the amino acid sequence was *cyclo*-G-L_a_-S-P-F-I-L_b_ (Figure 2), which was further confirmed by ESI–MS/MS fragmentation analysis (Figure S29). The observed Δ*δ*_Cβ-Cγ_ value (3.2 ppm) of the Pro^4^ and the ROE correlation from the Hα (δ_H_ 4.89) of Ser^3^ to the Hδ (δ_H_ 3.91 and 3.42) of Pro^4^ revealed that the geometry of Pro^4^ of **3** was a *trans* configuration [21,22,23]. The absolute configuration of **3** was determined by Marfey’s method [24,25,26,27], which indicated that all the amino acids were L configuration (Figure S30). Therefore, dianthiamide C (**3**) was confirmed as *cyclo*-(Gly^1^-l-Leu^2^-l-Ser^3^-l-*trans*-Pro^4^-l-Phe^5^-l-Ile^6^-l-Leu^7^).

Dianthiamide D (**4**), a yellow amorphous powder, gave the molecular formula C_36_H_60_N_8_O_8_, based on the HRESI–TOF–MS data (*m*/*z* 755.4448 [M + Na]^+^; calcd 755.4426). Detailed analyses of the 1D and 2D (COSY, HSQC, and HMBC) NMR data revealed that **4** was a octapeptide containing alanine (A), glycine (G), isoleucine (I), two prolines (P_a_ and P_b_) and three valines (V_a_, V_b_, and V_c_) residues (Table 4). The amino acid sequence of **4** was established as *cyclo*-G-A-V_a_-I-P_a_-V_b_-V_c_-P_b_ by analysis of HMBC and ROESY data (Figure 2). This conclusion was also supported by the ESI–MS/MS sequence analysis (Figure S39). The observed Δ*δ*_Cβ-Cγ_ values of the Pro_a_^5^ (2.6 ppm) and Pro_b_^8^ (4.0 ppm) and the ROE correlations from the Hα of Ile^4^ (δ_H_ 4.48) to the Hδ of Pro_a_^5^ (δ_H_ 3.79 and 3.62), and from the Hα of Val_c_^7^ (δ_H_ 4.43) to the Hδ of Pro_b_^8^ (δ_H_ 3.76 and 3.53) indicated that the geometry of both Pro_a_^5^ and Pro_b_^8^ of **4** were *trans* configuration [21,22,23]. Moreover, the absolute configuration of **4** was assigned by Marfey’s method [24,25,26,27], which indicated that all the amino acids had L configuration (Figure S40). Therefore, dianthiamide D (**4**) was established as *cyclo*-(Gly^1^-l-Ala^2^-l-Val_a_^3^-l-Ile^4^-l-*trans*-Pro_a_^5^-l-Val_b_^6^-l-Val_c_^7^-l-*trans*-Pro_b_^8^).

Dianthiamide E (**5**), a yellow amorphous powder. Its molecular formula of C_36_H_54_N_8_O_9_ was determined from the HRESI–TOF–MS data (*m*/*z* 743.4097 [M + H]^+^; calcd for 743.4086). Analysis of 1D and 2D (COSY, HSQC, and HMBC) NMR data (Table 5 and Figure 2) as well as ESI–MS/MS sequence data (Figure S49) demonstrated that the seven amino acid residues were phenylalanine (F), glycine (G), isoleucine (I), asparagine (N), leucine (L), proline (P), and threonine (T). The sequence of these amino acid residues was assigned as *cyclo*-G-N-P-L-T-I-F by the observed HMBC and ROESY data (Figure 2).

The small difference of the Δ*δ*_Cβ-Cγ_ values of the Pro^3^ (4.4 ppm) and the ROE correlations between the Hα of Asn^2^ (δ_H_ 4.79) and the Hδ of Pro^3^ (δ_H_ 3.73 and 3.56) revealed that the geometry of Pro^3^ of **5** was a *trans* [21,22,23]. Furthermore, Marfey’s analysis assigned L configurations to all the amino acid residues in **5** (Figure S50) [24,25,26,27]. Therefore, dianthiamide E (**5**) was established as *cyclo*-(Gly^1^-l-Asn^2^-l-*trans*-Pro^3^-l-Leu^4^-l-Thr^5^-l-Ile^6^-l-Phe^7^).

Recently, it has been reported that cyclic peptides isolated from the genus *Dianthus* exhibited cytotoxic activity against several cancer cell lines [5,6,18]. Therefore, all isolates were tested for their cytotoxic activity against human non-small cell lung cancer A549 and human stomach adenocarcinoma MKN-28 cells, with docetaxel as a positive control. However, dianthiamide A (**1**) only showed weak activity against A549 cell line with IC_50_ value of 47.9 μM, and docetaxel was used as a positive control (IC_50_: 0.08 μM). The other compounds **2**–**5** were inactive against A549 and MKN-28 cells (IC_50_: >200 μM).

## 3. Materials and Methods

### 3.1. Chemicals

HPLC grade acetonitrile was purchased from m Fisher Chemical (Loughborough, UK) and all other chromatographic solvents were purchased from Duksan Pure Chemicals Co., Cheongju, Korea). Paclitaxel as a positive control was obtained from LC Laboratories (Woburn, MA, USA). MTT (3-(4,5-Dimethylthiazol-2-yl)-2,5-diphenyltetrazolium bromide) was purchased from Sigma-Aldrich Corp. (St. Louis, MO, USA).

### 3.2. General Experimental Procedures

Optical rotations were measured on a JASCO DIP-1000 polarimeter. UV spectra were recorded on a JASCO UV-550 spectrophotometer. ECD spectra were obtained on a JASCO J-715 spectrometer, and IR spectra were measured on a JASCO FT-IR 4100 spectrometer (JASCO, Tokyo, Japan). NMR spectra were recorded on a Bruker AVANCE 700 MHz spectrometer (Bruker, MA, USA) using DMSO-*d*_6_ as solvent. ESI–MS and HRESI–TOF–MS were obtained with LCQ Fleet (Thermo Fisher Scientific, San Jose, CA, USA) and maXis 4G mass spectrometers (Bruker, Bremen, Germany), respectively. Column chromatography was performed on silica gel (70–230 mesh, Merck, Darmstadt, Germany) and Lichroprep RP-18 (40–63 μm, Merck, Darmstadt, Germany). MPLC was performed on a Biotage Isolera Prime chromatography system (Biotage, Uppsala, Sweden). Preparative HPLC was performed using Waters HPLC system equipped with two Waters 515 pumps with a 2996 photodiode-array detector (Waters Corporation, Milford, MA, USA) using an YMC J’sphere ODS-H80 column (4 μm, 150 × 20 mm, i.d., Kyoto, Japan, flow rate 6 mL/min). TLC was performed using precoated silica gel 60 F_254_ (0.25 mm, Merck, Darmstadt, Germany) plates, and spots were detected by a 10% vanillin-H_2_SO_4_ in water spray reagent.

### 3.3. Plant Material

The dried whole plants of *Dianthus chinensis* L. (Caryophyllaceae) were purchased from Kyungdong herbal market in Seoul, Korea, in June 2014. A voucher specimen (CBNU-2014-06-DC) was authenticated by B.Y.H. and deposited at the Herbarium of the College of Pharmacy, Chungbuk National University, Korea.

### 3.4. Isolation and Purification of Compounds **1**–**5**

The dried and powdered whole plants of *D. chinensis* (3.0 kg) were extracted with MeOH (3 × 16 L) at room temperature. The extract was evaporated under reduced pressure, and the residue (470 g) was suspended in water and partitioned successively with *n*-hexane (2 × 1.5 L), CH_2_Cl_2_ (2 × 1.5 L), and EtOAc (2 × 1.5 L). The CH_2_Cl_2_-soluble fraction (13 g) was separated by MPLC with Lichroprep RP-18 column and eluted with MeOH-H_2_O gradient system (10:90 to 100:0) to give eleven fractions (DCC1-DCC11). DCC1 (1.2 g) was separated on a silica gel column and eluted with CH_2_Cl_2_-MeOH gradient (from 100:0 to 0:100, 400 mL for each step) to obtain seven fractions (DCC1-1-DCC1-7) by MPLC. DCC1-4 (90 mg) was further purified by preparative HPLC (Waters system, YMC J’sphere ODS-H80, 150 × 20 mm i.d., MeCN-H_2_O, 30:70 to 60:40, flow rate 6 mL/min) to yield compound **1** (*t*_R_ = 20.1 min, 15 mg). DCC7 (1.0 g) was subjected to silica gel column chromatography and eluted with CH_2_Cl_2_-MeOH (from 100:0 to 0:100, 400 mL for each step) to give seven fractions (DCC7-1-DCC7-7) by MPLC. DCC7-4 (90 mg) was further purified by preparative HPLC (MeCN-H_2_O, 35:65 to 65:35) to yield compound **2** (*t*_R_ = 21.4 min, 17 mg). DCC9 (2.2 g) was separated on a silica gel column and eluted with CH_2_Cl_2_-MeOH gradient (from 100:0 to 0:100, 400 mL for each step) to obtain nine fractions (DCC9-1-DCC9-9) by MPLC. DCC9-7 (130 mg) was further purified by preparative HPLC (MeCN-H_2_O, 30:70 to 60:40) to afford compounds **3** (*t*_R_ = 18.9 min, 6 mg) and **5** (*t*_R_ = 23.1 min, 4 mg). DCC9-9 (90 mg) was further purified by preparative HPLC (MeCN-H_2_O, 30:70 to 60:40) to afford compound **4** (*t*_R_ = 21.9 min, 5 mg).

### 3.5. Characterization of Compounds **1**–**5**

Dianthiamide A (**1**, cyclo-(Gly^1^-l-Phe^2^-l-Leu^3^-l-*trans*-Pro_a_^4^-l-*cis*-Pro_b_^5^-l-Ile^6^-l-Asn^7^)), Yellow amorphous powder; [α]^25^_D_ -41.2 (*c* 0.05, MeOH); UV (MeOH) λ_max_ (log ε) 203 (3.65) nm; ECD (MeOH) λ_max_ (Δε) 201 (−10.9), 210 (−6.5), 219 (−7.8) nm; IR υ_max_ (film) 3330, 2944, 1657, 1530, 1454 cm^−1^; ^1^H NMR (700 MHz, DMSO-*d*_6_) and ^13^C NMR (175 MHz, DMSO-*d*_6_), see Table 1; ESI–MS *m*/*z* 739 [M + H]^+^; HRESI–TOF–MS *m*/*z* 739.4144 [M + H]^+^ (calcd for C_37_H_55_N_8_O_8_, 739.4137).

Dianthiamide B (**2**, cyclo-(Gly^1^-l-Leu^2^-l-*trans*-Pro^3^-l-Phe^4^-l-Asp^5^-l-Ile^6^)), Yellow amorphous powder; [α]^25^_D_ -20.0 (*c* 0.05, MeOH); UV (MeOH) λ_max_ (log ε) 202 (3.70) nm; ECD (MeOH) λ_max_ (Δε) 201 (−3.2), 205(−1.5), 217 (−4.2) nm; IR υ_max_ (film) 3312, 2972, 1644, 1530, 1448 cm^−1^; ^1^H NMR (700 MHz, DMSO-*d*_6_) and ^13^C NMR (175 MHz, DMSO-*d*_6_), see Table 2; ESI–MS *m*/*z* 647 [M + Na]^+^; HRESI–TOF–MS *m*/*z* 647.3161 [M + Na]^+^ (calcd for C_32_H_44_N_6_NaO_7_, 647.3163).

Dianthiamide C (**3**, cyclo-(Gly^1^-l-Leu^2^-l-Ser^3^-l-*trans*-Pro^4^-l-Phe^5^-l-Ile^6^-l-Leu^7^)), Yellow amorphous powder; [α]^25^_D_ -45.2 (*c* 0.05, MeOH); UV (MeOH) λ_max_ (log ε) 204 (3.24) nm; ECD (MeOH) λ_max_ (Δε) 207 (+1.4), 226 (−8.6) nm; IR υ_max_ (film) 3311, 2944, 1644, 1530, 1462 cm^−1^; ^1^H NMR (700 MHz, DMSO-*d*_6_) and ^13^C NMR (175 MHz, DMSO-*d*_6_), see Table 3; ESI–MS *m*/*z* 750 [M + Na]^+^; HRESI–TOF–MS *m*/*z* 750.4165 [M + Na]^+^ (calcd for C_37_H_57_N_7_NaO_8_, 750.4160).

Dianthiamide D (**4**, cyclo-(Gly^1^-l-Ala^2^-l-Val_a_^3^-l-Ile^4^-l-*trans*-Pro_a_^5^-l-Val_b_^6^-l-Val_c_^7^-l-*trans*-Pro_b_^8^)), Yellow amorphous powder; [α]^25^_D_ -37.8 (*c* 0.05, MeOH); UV (MeOH) λ_max_ (log ε) 210 (3.44) nm; ECD (MeOH) λ_max_ (Δε) 201 (+9.1), 219 (−7.1) nm; IR υ_max_ (film) 3312, 2943, 1644, 1548, 1454 cm^−1^; ^1^H NMR (700 MHz, DMSO-*d*_6_) and ^13^C NMR (175 MHz, DMSO-*d*_6_), see Table 4; ESI–MS *m*/*z* 755 [M + Na]^+^; HRESI–TOF–MS *m*/*z* 755.4448 [M + Na]^+^ (calcd for C_36_H_60_N_8_NaO_8_, 755.4426).

Dianthiamide E (**5**, cyclo-(Gly^1^-l-Asn^2^-l-*trans*-Pro^3^-l-Leu^4^-l-Thr^5^-l-Ile^6^-l-Phe^7^)), Yellow amorphous powder; [α]^25^_D_ -41.2 (*c* 0.05, MeOH); UV (MeOH) λ_max_ (log ε) 203 (3.47) nm; ECD (MeOH) λ_max_ (Δε) 201 (−5.8) nm; IR υ_max_ (film) 3309, 2942, 1741, 1644, 1548 cm^−1^; ^1^H NMR (700 MHz, DMSO-*d*_6_) and ^13^C NMR (175 MHz, DMSO-*d*_6_), see Table 5; ESI–MS *m*/*z* 743 [M + H]^+^; HRESI–TOF–MS *m*/*z* 743.4097 [M + H]^+^ (calcd for C_36_H_55_N_8_O_9_, 743.4086).

### 3.6. Absolute Configuration of Amino Acids in **1**–**5** Using Marfey’s Method

Compounds **1**–**5** (0.5 mg) were hydrolyzed in 1 mL of 6 N HCl at 105 °C for 12 h. After cooling to room temperature, the hydrolysate was evaporated to dryness and redissolved in 200 μL of water and 1 M NaHCO_3_ (20 μL). A solution of *N*-*α*-(2,4-dinitro-5-fluorophenyl)-l-alaninamide (l-FDAA, Marfey’s reagent, Sigma, 100 μL, 1%) in acetone was added to each reaction vial. The reaction mixture was heated at 37 °C for 1 h, quenched by adding 1 N HCl (20 μL), and then dissolved in CH_3_CN (800 μL). A volume of 5 μL of the FDAA derivatives were analyzed by LC/MS (YMC UltraHT Pro C_18_, S-2 μm, 12 nm, 50 × 2.0 mm, flow rate: 0.2 mL/min) at RT, and monitored by UV absorption at 340 nm. Aqueous CH_3_CN containing 0.1% TFA was used as the mobile phase in a gradient mode (10–50% CH_3_CN for 0–40 min). From each standard, 50 mM aqueous solution of d- or l-amino acid (Ala, Asp, Phe, Ile, *allo*-Ile, Leu, Asn, Pro, Ser, Thr, *allo*-Thr, and Val) were taken, and 1 M NaHCO_3_ (20 μL) and a solution of *N*-*α*-(2,4-dinitro-5-fluorophenyl)-l-alaninamide (l-FDAA, Marfey’s reagent, Sigma, 100 μL, 1%) in acetone was added. The reaction mixture was heated at 37 ^o^C for 1 h, quenched by adding 1 N HCl (20 μL), and then dissolved in CH_3_CN (800 μL). A volume of 5 μL of the FDAA derivatives were analyzed by LC/MS (YMC UltraHT Pro C_18_, S-2 μm, 12 nm, 50 × 2.0 mm, flow rate: 0.2 mL/min) at RT and monitored by UV absorption at 340 nm. Aqueous CH_3_CN containing 0.1% TFA was used as the mobile phase in a gradient mode (10–50% CH_3_CN for 0–40 min). The following retention times (min) were observed for the l-FDAA derivatives of the standards, respectively: 19.5 (l-Ala) and 22.7 (d-Ala), 15.0 (l-Asp) and 18.1 (d-Asp), 29.7 (l-Phe) and 33.4 (d-Phe), 29.5 (l-Leu) and 34.4 (d-Leu), 30.3 (l-Ile), 31.0(l-*allo*-Ile) and 35.2 (d-Ile), 20.8 (l-Pro) and 22.1 (d-Pro), 13.6 (l-Ser) and 14.5 (d-Ser), 14.7 (l-Thr), 15.2 (l-*allo*-Thr) and 18.2 (d-Thr), and 25.4 (l-Val) and 29.6 (d-Val).

### 3.7. Cytotoxicity Assay

Human non-small cell lung cancer A549 and human stomach adenocarcinoma MKN-28 cells were purchased from the American Type Culture Collection (ATCC; Manassas, VA, USA). A549 and MKN-28 cells were cultured as monolayers in RPMI1640 medium containing 10% fetal bovine serum (FBS), 1% penicillin/streptomycin at 37 °C in a humidified atmosphere of 5% CO_2_. Growth-inhibitory effect of the isolated compounds on A549 cells and MKN-28 cells was evaluated using MTT assay [28]. Briefly, 5 × 10^3^ cells of A549 cells or MKN-28 cells were seeded in each well of a 96-well plate, respectively, and incubated for 24 h. A549 cells or MKN-28 cells were then treated with various concentrations of compounds **1–5**. The concentration range of the compound tested for the evaluation of the cytotoxic activity was 5–200 µM. After incubation of 48 h, MTT (3-(4,5-Dimethylthiazol-2-yl)-2,5-diphenyltetrazolium bromide) solution was added to each well and the plate was incubated for 4 h. The medium in each well was replaced with dimethyl sulfoxide (DMSO) to dissolve blue formazan crystals. The absorbance at 540 nm was measured using microplate reader (Molecular devices; SpectraMax, CA, USA). All data processing and IC_50_ values were analyzed using GraphPad Prism v.5 (GraphPad Software, La Jolla, CA, USA). Docetaxel was used as a positive control with an IC_50_ value of 0.08 μM on A549 cells [29].

## 4. Conclusions

We report the isolation and structure determination of five undescribed orbitides, dianthiamides A–E, from the whole plants of *D. chinensis*. The previously reported orbitides isolated from the genus *Dianthus* tend to have five to six amino acid residues [4,5,6,7,8,9]. All orbitides in this study, however, are characteristic of the presence of six to eight amino acids, while they are featured by containing at least one proline residue. All isolates were tested for their cytotoxic activity, and dianthiamide A (**1**) exhibited weak activity against the A549 cell line. Furthermore, from a chemotaxonomical point of view, it is noteworthy that this finding expands the orbitides diversity in the genus *Dianthus*.

## Figures and Tables

**Table 1:** ^1^H and ^13^C NMR data for dianthiamide A (**1**) (DMSO-*d*_6_, 700 MHz, d in ppm, *J* in Hz).

Position	δ_C_	δ_H_ (*J* in Hz)	Position	δ_C_	δ_H_ (*J* in Hz)
**Gly^1^**			***cis*** **-Pro_b_^5^**		
C=O	169.8, C		C=O	170.9, C	
NH		8.70 (t, 4.2)	*α*	60.8, CH	4.55 (d, 7.0)
*α*	44.4, CH_2_	3.55 (dd, 16.8, 4.2)	*β*	31.1, CH_2_	2.33 (dd, 11.9, 7.0)
		3.40 (dd, 16.8, 5.6)			1.92 (m)
**Phe^2^**			*γ*	22.1, CH_2_	1.85 (m)
C=O	171.1, C				1.46 (m)
NH		7.69 (d, 9.1)	*δ*	46.4, CH_2_	3.46 (m)
*α*	56.0, CH	4.38 (m)			3.25 (t, 9.8)
*β*	37.0, CH_2_	3.01 (m)	**Ile^6^**		
		2.97 (m)	C=O	171.4, C	
Phe-1′	138.4, C		NH		8.49 (d, 7.7)
2′,6′	129.1, CH	7.15–7.27 (m)	*α*	61.6, CH	3.66 (dd, 9.8, 7.7)
3′,5′	128.8, CH	7.15–7.27 (m)	*β*	35.3, CH	2.05 (m)
4′	126.8, CH	7.15–7.27 (m)	*γ*-CH_3_	16.0, CH_3_	0.90 (t, 7.7)
**Leu^3^**					
C=O	168.8, C		*γ*-CH_2_	25.9, CH_2_	1.50 (m)
NH		7.28 (m)			1.15 (m)
*α*	48.6, CH	4.48 (m)	*δ*-CH_3_	10.9, CH_3_	0.82 (d, 7.7)
*β*	41.0, CH_2_	1.68 (m)	**Asn^7^**		
		1.19 (m)	C=O	172.3, C	
*γ*-CH	24.6, CH	1.51 (m)	NH		7.26 (m)
*δ*-CH_3_	23.6, CH_3_	0.85 (d, 6.3)	*α*	49.6, CH	4.18 (m)
*δ*-CH_3_	22.6, CH_3_	0.88 (d, 6.3)	*β*	35.8, CH_2_	3.15 (m)
***trans*-Pro_a_^4^**					3.05 (m)
C=O	170.8, C		C=O	173.4, C	
*α*	59.6, CH	4.45 (t, 7.0)	NH_2_		7.97 (s)
*β*	28.5, CH_2_	2.27 (m)			7.54 (s)
		1.64 (m)			
*γ*	25.3, CH_2_	1.96 (m)			
		1.83 (m)			
*δ*	47.1, CH_2_	3.44 (m)			

**Table 2:** ^1^H and ^13^C NMR data for dianthiamide B (**2**) (DMSO-*d*_6_, 700 MHz, d in ppm, *J* in Hz).

Position	δ_C_	δ_H_ (*J* in Hz)	Position	δ_C_	δ_H_ (*J* in Hz)
**Gly^1^**			**Phe^4^**		
C=O	168.7, C		C=O	170.7, C	
					
NH		8.68 (m)	NH		8.68 (m)
*α*	43.3, CH_2_	3.65 (t, 7.0)	*α*	57.2, CH	3.80 (m)
**Leu^2^**			*β*	33.8, CH_2_	3.33 (m)
C=O	170.7, C				3.30 (m)
NH		6.50 (m)	Phe-1′	139.6, C	
*α*	48.6, CH	4.51 (m)	2′,6′	129.6, CH	7.10–7.30 (m)
*β*	40.5, CH_2_	1.40 (m)	3′,5′	128.6, CH	7.10–7.30 (m)
		1.20 (m)	4′	126.6, CH	7.10–7.30 (m)
*γ*-CH	24.6, CH	1.62 (m)	**Asp^5^**		
*δ*-CH_3_	23.8, CH_3_	0.91 (d, 7.0)	C=O	176.2, C	
*δ*-CH_3_	21.3, CH_3_	0.93 (d, 7.0)	NH		7.95 (d, 8.4)
***trans*-Pro^3^**			*α*	47.3, CH	5.23 (m)
C=O	171.7, C		*β*	36.5, CH_2_	3.22 (m)
*α*	61.0, CH	3.92 (t, 7.0)			2.05 (d, 3.5)
*β*	29.3, CH_2_	1.85 (m)	C=O	177.2, C	
		1.67 (m)	**Ile^6^**		
*γ*	25.4, CH_2_	2.01 (m)	C=O	167.0, C	
		1.83 (m)	*α*	61.5, CH	4.19 (d, 11.9)
*δ*	47.5, CH_2_	3.68 (m)	*β*	30.4, CH	2.68 (m)
		3.42 (m)	*γ*-CH_3_	16.1, CH_3_	0.87 (t, 7.7)
			*γ*-CH_2_	24.3, CH_2_	1.38 (m)
					0.95 (m)
			*δ*-CH_3_	10.5, CH_3_	0.79 (d, 7.7)

**Table 3:** ^1^H and ^13^C NMR data for dianthiamide C (**3**) (DMSO-*d*_6_, 700 MHz, d in ppm, *J* in Hz).

Position	δ_C_	δ_H_ (*J* in Hz)	Position	δ_C_	δ_H_ (*J* in Hz)
**Gly^1^**			**Phe^5^**		
C=O	169.1, C		C=O	170.7, C	
NH		8.91 (t, 4.9)	NH		7.43 (d, 9.8)
*α*	43.2, CH	4.00 (m)	*α*	54.6, CH	4.38 (m)
		3.33 (m)	*β*	38.4, CH_2_	3.21 (dd, 13.3, 3.5)
**Leu_a_^2^**					2.62 (t, 13.3)
C=O	171.2, C		Phe-1′	138.4, C	
NH		8.10 (d, 10.5)	2′,6′	129.4, CH	7.18–7.31 (m)
*α*	53.0, CH	4.52 (m)	3′,5′	128.6, CH	7.18–7.31 (m)
*β*	43.8, CH_2_	1.42 (m)	4′	126.8, CH	7.18–7.31 (m)
		1.34 (m)	**Ile^6^**		
*γ*-CH	24.5, CH	1.55 (m)	C=O	171.6, C	
*δ*-CH_3_	22.9, CH_3_	0.87 (d, 6.3)	NH		7.01 (d, 9.1)
*δ*-CH_3_	22.2, CH_3_	0.84 (d, 6.3)	*α*	56.4, CH	4.35 (m)
**Ser^3^**			*β*	37.9, CH	1.71 (m)
C=O	168.9, C		*γ*-CH_3_	15.1, CH_3_	0.82 (t, 7.7)
NH		8.73 (d, 7.0)	*γ*-CH_2_	24.7, CH_2_	1.39 (m)
*α*	54.2, CH	4.89 (br s)			1.00 (m)
*β*	61.9, CH_2_	4.24 (m)	*δ*-CH_3_	11.0, CH_3_	0.81 (d, 7.7)
		3.71 (d, 10.5)	**Leu_b_^7^**		
***trans*-Pro^4^**			C=O	172.3, C	
C=O	170.5, C		NH		8.71 (br s)
*α*	62.2, CH	3.98 (m)	*α*	54.2, CH	3.87 (m)
*β*	29.0, CH_2_	1.97 (m)	*β*	40.1, CH_2_	1.47 (m)
		1.24 (m)	*γ*-CH	24.4, CH	1.54 (m)
*γ*	25.8, CH_2_	1.79 (m)	*δ*-CH_3_	23.4, CH_3_	0.86 (m)
		1.68 (m)	*δ*-CH_3_	22.6, CH_3_	0.93 (m)
*δ*	47.8, CH_2_	3.91 (m)			
		3.42 (m)			

**Table 4:** ^1^H and ^13^C NMR data for dianthiamide D (**4**) (DMSO-*d*_6_, 700 MHz, d in ppm, *J* in Hz).

Position	δ_C_	δ_H_ (*J* in Hz)	Position	δ_C_	δ_H_ (*J* in Hz)
**Gly^1^**			***trans*-Pro^5^**		
C=O	168.4, C		C=O	170.7, C	
NH		8.97 (t, 4.9)	*α*	62.0, CH	4.29 (m)
*α*	43.2, CH	3.84 (dd, 14.7, 4.9)	*β*	27.6, CH_2_	2.11 (m)
		3.33 (dd, 14.7, 4.2)			1.91 (m)
**Ala^2^**			*γ*	25.0, CH_2_	1.89 (m)
C=O	173.1, C				1.79 (m)
NH		7.30 (d, 7.0)	*δ*	48.2, CH_2_	3.79 (m)
*α*	47.2, CH	4.62 (t, 7.0)			3.62 (m)
*β*	19.6, CH_3_	1.42 (d, 7.0)	**Val^6^**		
**Val^3^**			C=O	171.6, C	
C=O	171.4, C		NH		8.24 (d, 7.0)
NH		7.99 (d, 4.9)	*α*	60.0, CH	3.89 (dd, 7.0, 4.2)
*α*	60.8, CH	3.71 (t, 4.9)	*β*	29.3, CH	2.23 (m)
*β*	28.7, CH	2.14 (m)	*γ*-CH_3_	20.1, CH_3_	0.90 (m)
*γ*-CH_3_	19.9, CH_3_	0.92 (d, 6.3)	*γ*-CH_3_	18.4, CH_3_	0.85 (d, 5.6)
*γ*-CH_3_	19.0, CH_3_	0.93 (d, 6.3)	**Val^7^**		
**Ile^4^**			C=O	169.8, C	
C=O	173.0, C		NH		7.16 (d, 7.0)
NH		6.70 (d, 7.7)	*α*	55.6, CH	4.43 (t, 7.0)
*α*	54.7, CH	4.48 (d, 7.7)	*β*	31.1, CH	2.02 (m)
*β*	35.9, CH	1.81 (m)	*γ*-CH_3_	19.9, CH_3_	0.86 (m)
*γ*-CH_3_	15.6, CH_3_	0.88 (t, 5.6)	*γ*-CH_3_	18.4, CH_3_	0.75 (d, 5.6)
*γ*-CH_2_	24.3, CH_2_	1.49 (m)	***trans*-Pro^8^**		
		1.08 (m)	C=O	173.0, C	
*δ*-CH_3_	11.0, CH_3_	0.83 (d, 7.0)	*α*	60.8, CH	4.18 (m)
			*β*	29.4, CH_2_	2.08 (m)
					1.74 (m)
			*γ*	25.4, CH_2_	1.99 (m)
					1.84 (m)
			*δ*	47.9, CH_2_	3.76 (m)
					3.53 (m)

**Table 5:** ^1^H and ^13^C NMR data for dianthiamide E (**5**) (DMSO-*d*_6_, 700 MHz, d in ppm, *J* in Hz).

Position	δ_C_	δ_H_ (*J* in Hz)	Position	δ_C_	δ_H_ (*J* in Hz)
**Gly^1^**			**Thr^5^**		
C=O	169.2, C		C=O	170.7, C	
NH		8.01 (m)	NH		7.57 (d, 9.1)
*α*	43.6, CH_2_	4.02 (m)	*α*	57.0, CH	4.67 (m)
		3.31 (m)	*β*	68.7, CH	4.25 (m)
**Asn^2^**			*γ*-CH_3_	19.4, CH_3_	1.03 (d, 6.3)
C=O	171.0, C		OH		5.26 (d, 6.3)
NH		7.15	**Ile^6^**		
*α*	48.8, CH	4.79 (dd, 14.0, 7.0)	C=O	171.1, C	
*β*	37.2, CH_2_	2.75 (m)	NH		8.00 (m)
		2.57 (m)	*α*	59.2, CH	3.95 (t, 5.6)
C=O	172.2, C		*β*	35.7, CH	1.88 (m)
NH_2_		7.75 (br s)	*γ*-CH_3_	15.9, CH_3_	0.70 (d, 7.0)
		7.20 (m)	*γ*-CH_2_	23.9, CH_2_	0.97 (m)
***trans*-Pro^3^**			*δ*-CH_3_	11.8, CH_3_	0.68 (d, 7.0)
C=O	171.5, C		**Phe^7^**		
*α*	62.1, CH	4.11 (t, 7.7)	C=O	171.6, C	
*β*	29.7, CH_2_	2.20 (m)	NH		7.78 (d, 2.1)
		1.71 (m)	*α*	54.9, CH	4.41 (m)
*γ*	25.3, CH_2_	1.92 (m)	*β*	37.3, CH_2_	3.13 (dd, 14.0, 5.6)
*δ*	47.5, CH_2_	3.73 (m)			2.84 (dd, 14.0, 9.1)
		3.56 (m)	Phe-1′	138.0, C	
**Leu^4^**			2′,6′	129.4, CH	7.17–7.29 (m)
C=O	172.2, C		3′,5′	128.7, CH	7.17–7.29 (m)
NH		8.39 (br s)	4′	126.8, CH	7.17–7.29 (m)
*α*	52.4, CH	4.00 (m)			
*β*	39.2, CH_2_	1.79 (m)			
		1.63 (m)			
*γ*	25.1, CH	1.54 (m)			
*δ*-CH_3_	23.7, CH_3_	0.88 (d, 7.0)			
*δ*-CH_3_	21.3, CH_3_	0.82 (d, 7.0)			

## Data Availability

The data is contained within the article or Supplementary Material.

## Supplementary Materials

The following are available online, Figures S1–S50: ^1^H, ^13^C-NMR, COSY, HSQC, HMBC, ROESY, ECD, HRESI–MS, ESI–MS/MS spectra, and Marfey’s analysis data of new compounds **1**–**5**.

## Sample Availability

Samples of the compounds are not available from the authors.
