# Supplementary material for: Dianthiamides A–E, Proline-Containing Orbitides from Dianthus chinensis
Source: Molecules. 2021 Nov 30;26(23):7275. doi: 10.3390/molecules26237275 (PMC8659278; doi:10.3390/molecules26237275)
Supplement: Supplementary file 1 [file molecules-26-07275-s001.zip › molecules-1469025-supplementary.pdf]

Supplementary Materials for

Dianthiamides A–E, Proline-Containing Orbitides from *Dianthus Chinensis*

Jin Woo Lee [1], Jun Gu Kim [1], Jae Sang Han [1], Yong Beom Cho [1], Yu Jin Lee [1], Dongho Lee [2], Dae Hwan Shin [1], Jin Tae Hong [1], Mi Kyeong Lee [1] and Bang Yeon Hwang [1,*]

[1] College of Pharmacy, Chungbuk National University, Cheongju 28160, Korea; jinu15@hanmail.net (J.W.L); sossgi@naver.com (J.G.K.); han00768@hanmail.net (J.S.H.); bum94@naver.com (Y.B.C.); yujinli@naver.com (Y.J.L.); dshin@chungbuk.ac.kr (D.H.S.); jinthong@chungbuk.ac.kr (J.T.H.); mklee@chungbuk.ac.kr (M.K.L.)

[2] Department of Plant Biotechnology, College of Life Sciences and Biotechnology, Korea University, Seoul 02841, Korea; dongholee@korea.ac.kr

* Correspondence: byhwang@chungbuk.ac.kr; Tel.: +82-43-261-2814

Figure S1. ^{1}H NMR spectrum of compound **1** (DMSO-d_6, 700 MHz)············ S4
Figure S2. ^{13}C NMR spectrum of compound **1** (DMSO-d_6, 175 MHz)············ S5
Figure S3. COSY spectrum of compound **1** ············ S6
Figure S4. HSQC spectrum of compound **1** ············ S7
Figure S5. HMBC spectrum of compound **1** ············ S8
Figure S6. ROESY spectrum of compound **1**············ S9
Figure S7. ECD spectrum of compound **1** ············ S10
Figure S8. HRESI-MS spectrum of compound **1** ············ S11
Figure S9. ESI-MS/MS spectrum of compound **1** ············ S12
Figure S10. Marfey's analysis of compound **1** ············ S13
Figure S11. ^{1}H NMR spectrum of compound **2** (DMSO-d_6, 700 MHz) ············ S14
Figure S12. ^{13}C NMR spectrum of compound **2** (DMSO-d_6, 175 MHz) ············ S15
Figure S13. COSY spectrum of compound **2**············ S16
Figure S14. HSQC spectrum of compound **2**············ S17
Figure S15. HMBC spectrum of compound **2**············ S18
Figure S16. ROESY spectrum of compound **2** ············ S19
Figure S17. ECD spectrum of compound **2**············ S20
Figure S18. HRESI-MS spectrum of compound **2** ············ S21
Figure S19. ESI-MS/MS spectrum of compound **2** ············ S22
Figure S20. Marfey's analysis of compound **2** ············ S23
Figure S21. ^{1}H NMR spectrum of compound **3** (DMSO-d_6, 700 MHz) ············ S24
Figure S22. ^{13}C NMR spectrum of compound **3** (DMSO-d_6, 175 MHz)············ S25
Figure S23. COSY spectrum of compound **3**············ S26
Figure S24. HSQC spectrum of compound **3**············ S27
Figure S25. HMBC spectrum of compound **3**············ S28
Figure S26. ROESY spectrum of compound **3** ············ S29
Figure S27. ECD spectrum of compound **3**············ S30
Figure S28. HRESI-MS spectrum of compound **3** ············ S31
Figure S29. ESI-MS/MS spectruma of compound **3**············ S32
Figure S30. Marfey's analysis of compound **3** ············ S33

Figure S31. ^{1}H NMR spectrum of compound **4** (DMSO-d_6, 700 MHz) ························ S34
Figure S32. ^{13}C NMR spectrum of compound **4** (DMSO-d_6, 175 MHz) ························ S35
Figure S33. COSY spectrum of compound **4** ·· S36
Figure S34. HSQC spectrum of compound **4** ·· S37
Figure S35. HMBC spectrum of compound **4** ·· S38
Figure S36. ROESY spectrum of compound **4** ·· S39
Figure S37. ECD spectrum of compound **4** ·· S40
Figure S38. HRESI-MS spectrum of compound **4** ·· S41
Figure S39. ESI-MS/MS spectrum of compound **4** ·· S42
Figure S40. Marfey's analysis of compound **4** ·· S43
Figure S41. ^{1}H NMR spectrum of compound **5** (DMSO-d_6, 700 MHz) ························ S44
Figure S42. ^{13}C NMR spectrum of compound **5** (DMSO-d_6, 175 MHz) ························ S45
Figure S43. COSY spectrum of compound **5** ·· S46
Figure S44. HSQC spectrum of compound **5** ·· S47
Figure S45. HMBC spectrum of compound **5** ·· S48
Figure S46. ROESY spectrum of compound **5** ·· S49
Figure S47. ECD spectrum of compound **5** ·· S50
Figure S48. HRESI-MS spectrum of compound **5** ·· S51
Figure S49. ESI-MS/MS spectrum of compound **5** ·· S52
Figure S50. Marfey's analysis of compound **5** ·· S53

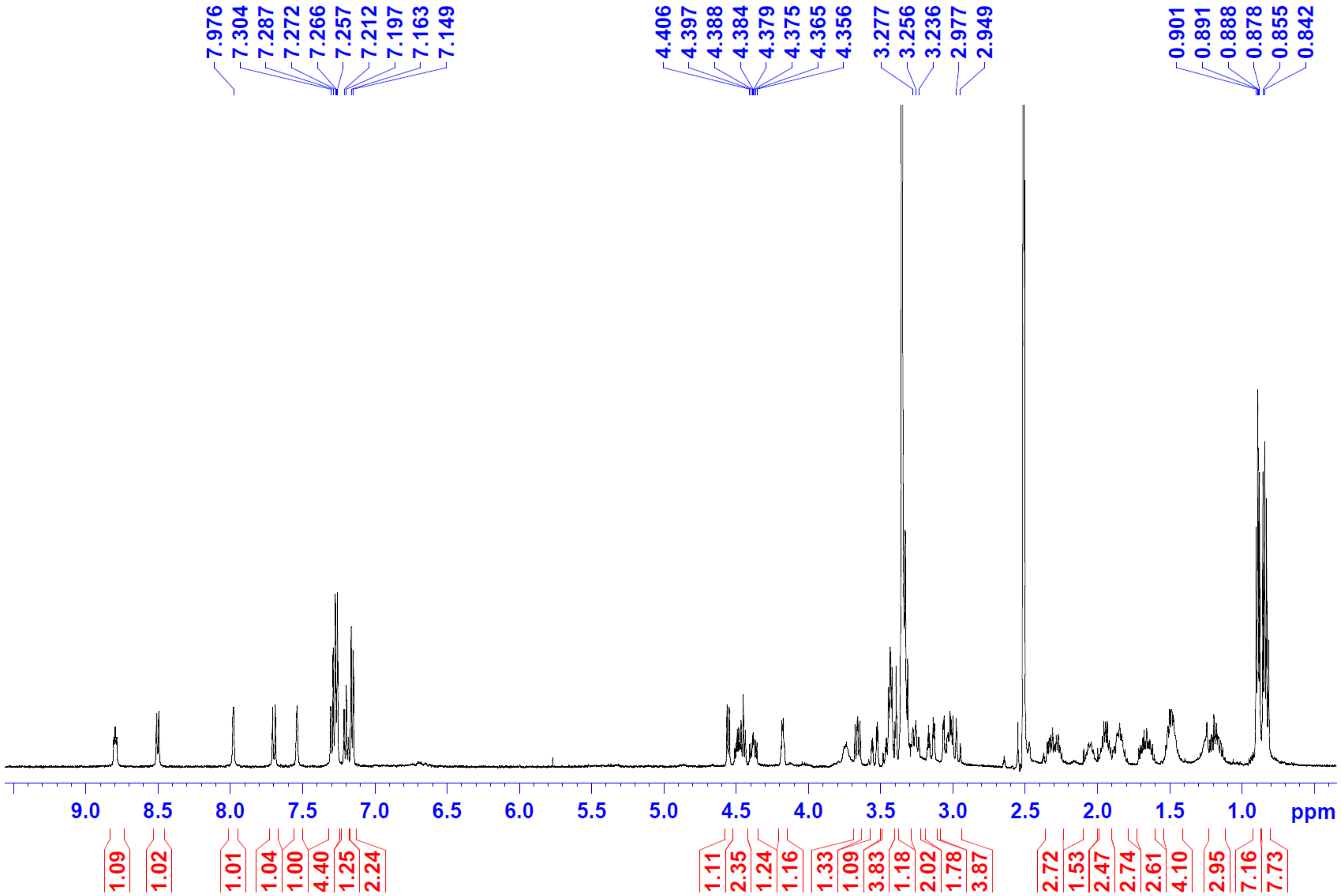

Figure S1. ^{1}H NMR spectrum of compound **1** (DMSO-d_6, 700 MHz)

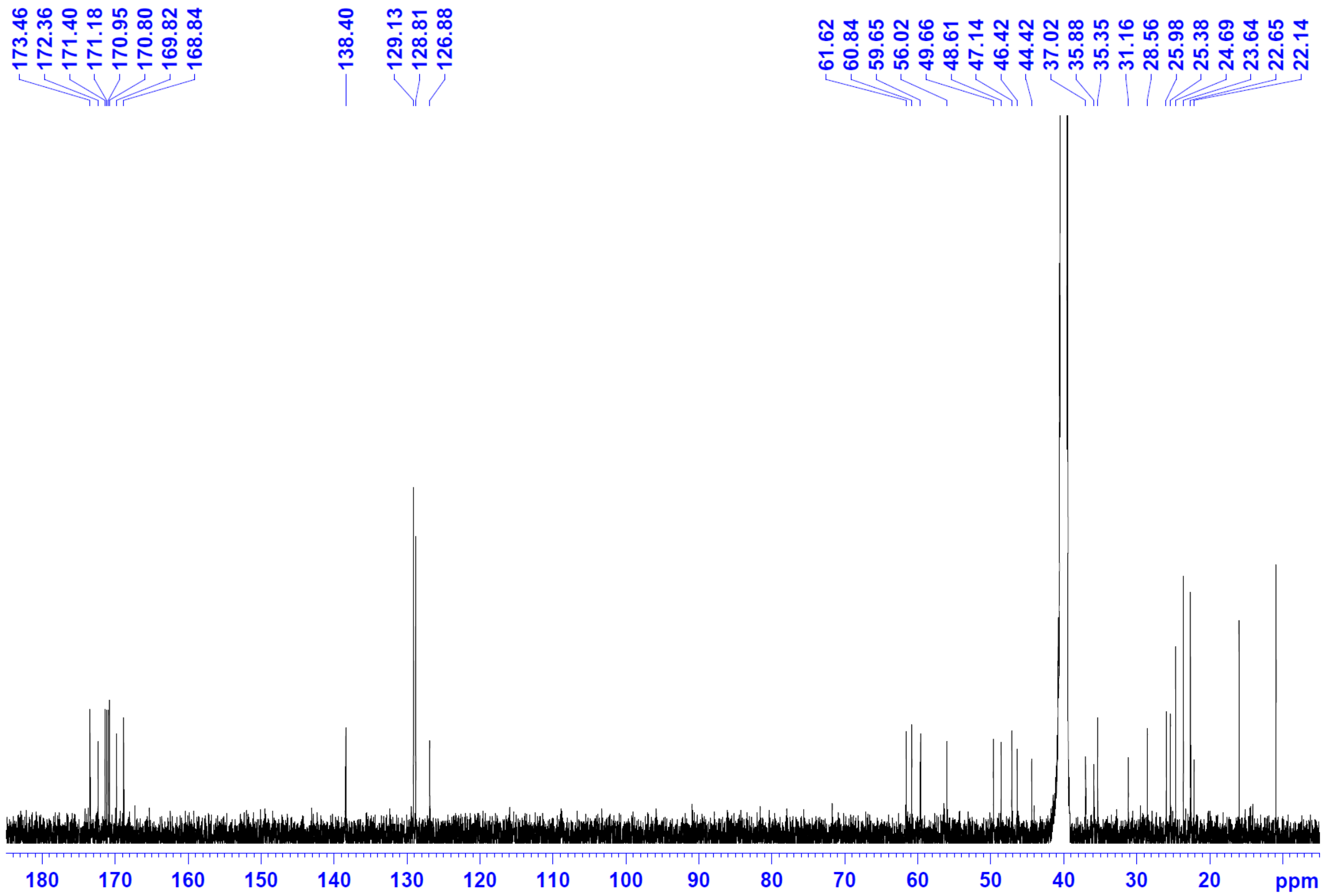

Figure S2. ^{13}C NMR spectrum of compound **1** (DMSO-d_6, 175 MHz)

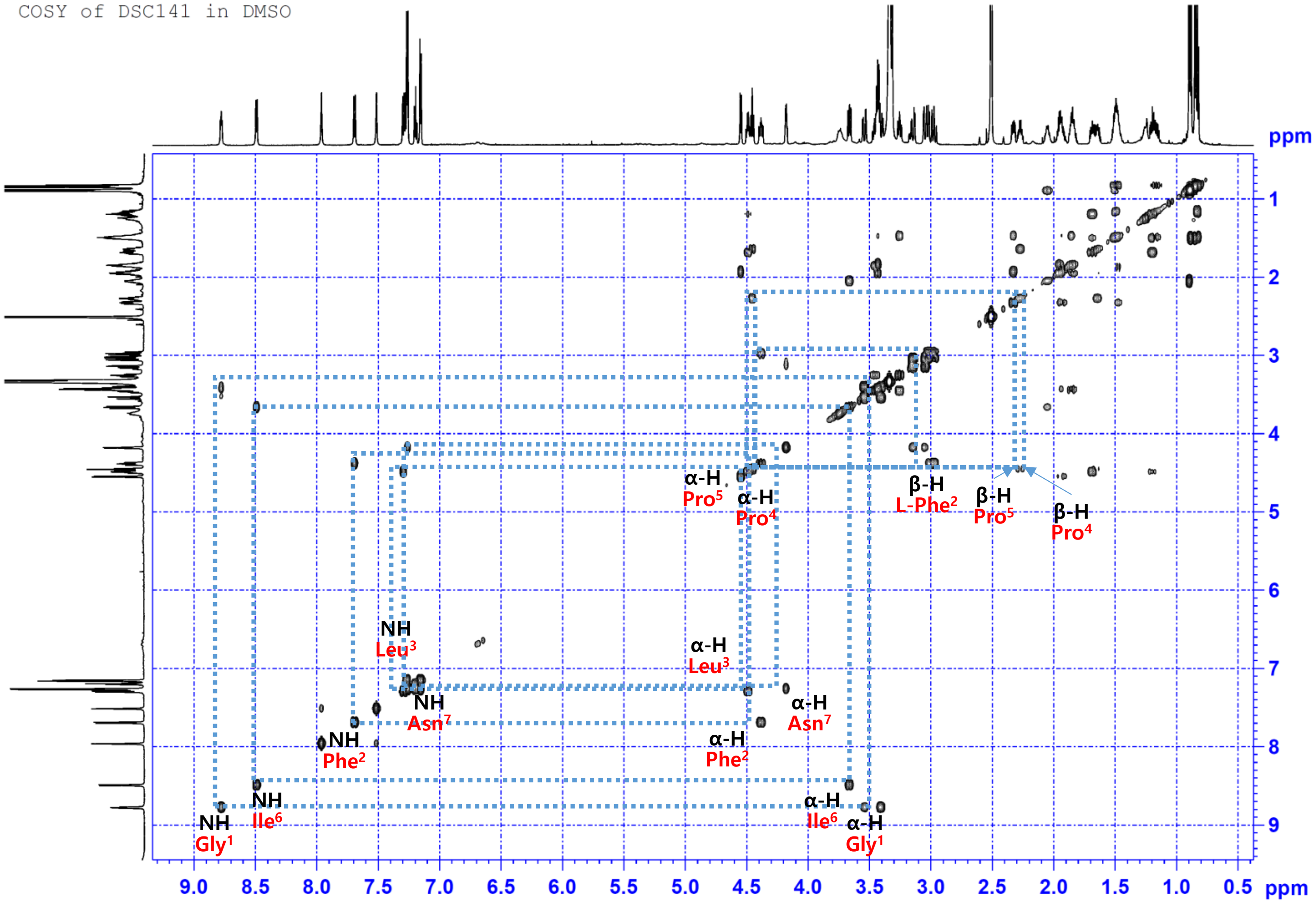

Figure S3. COSY spectrum of compound **1**

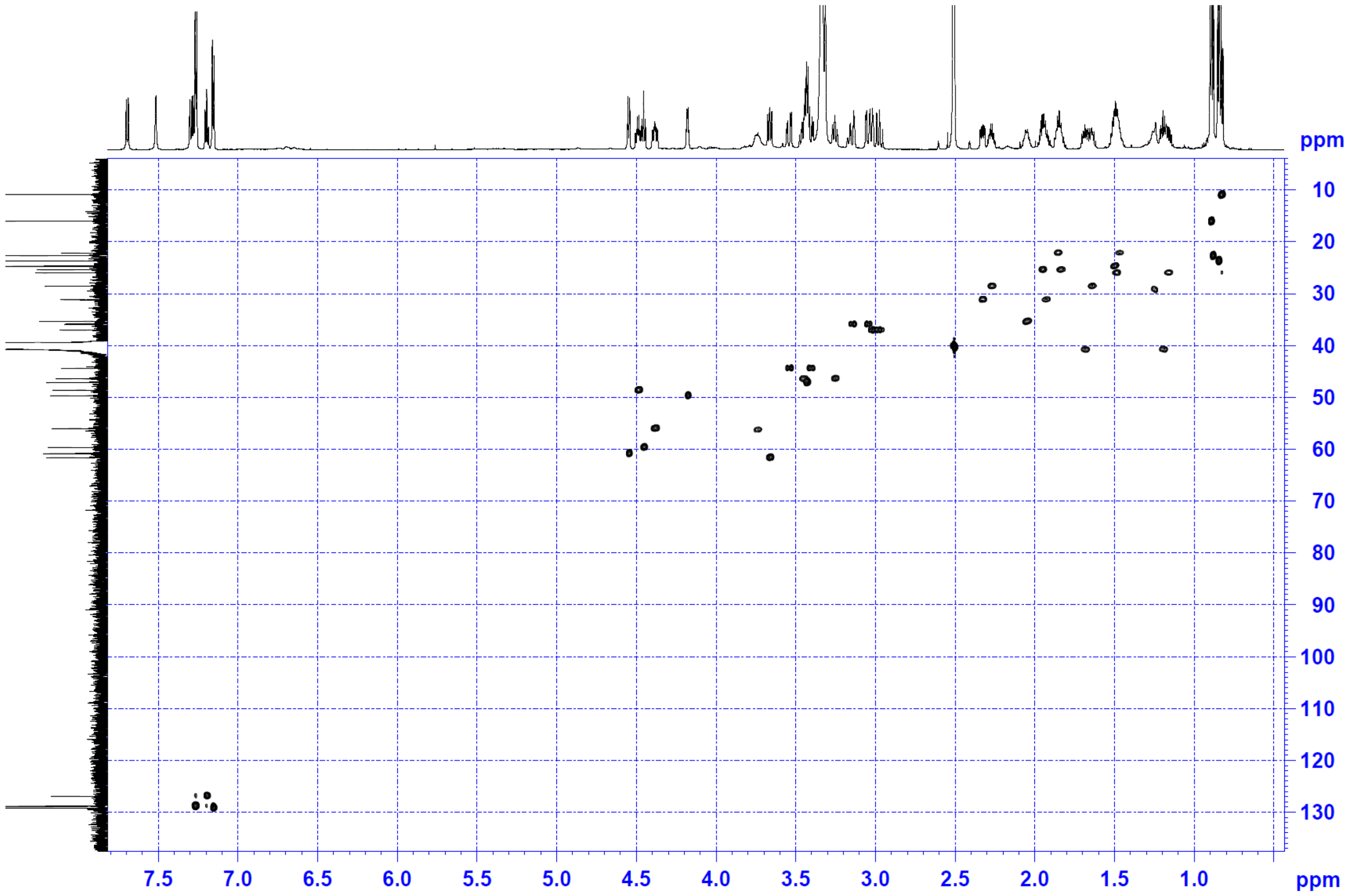

Figure S4. HSQC spectrum of compound **1**

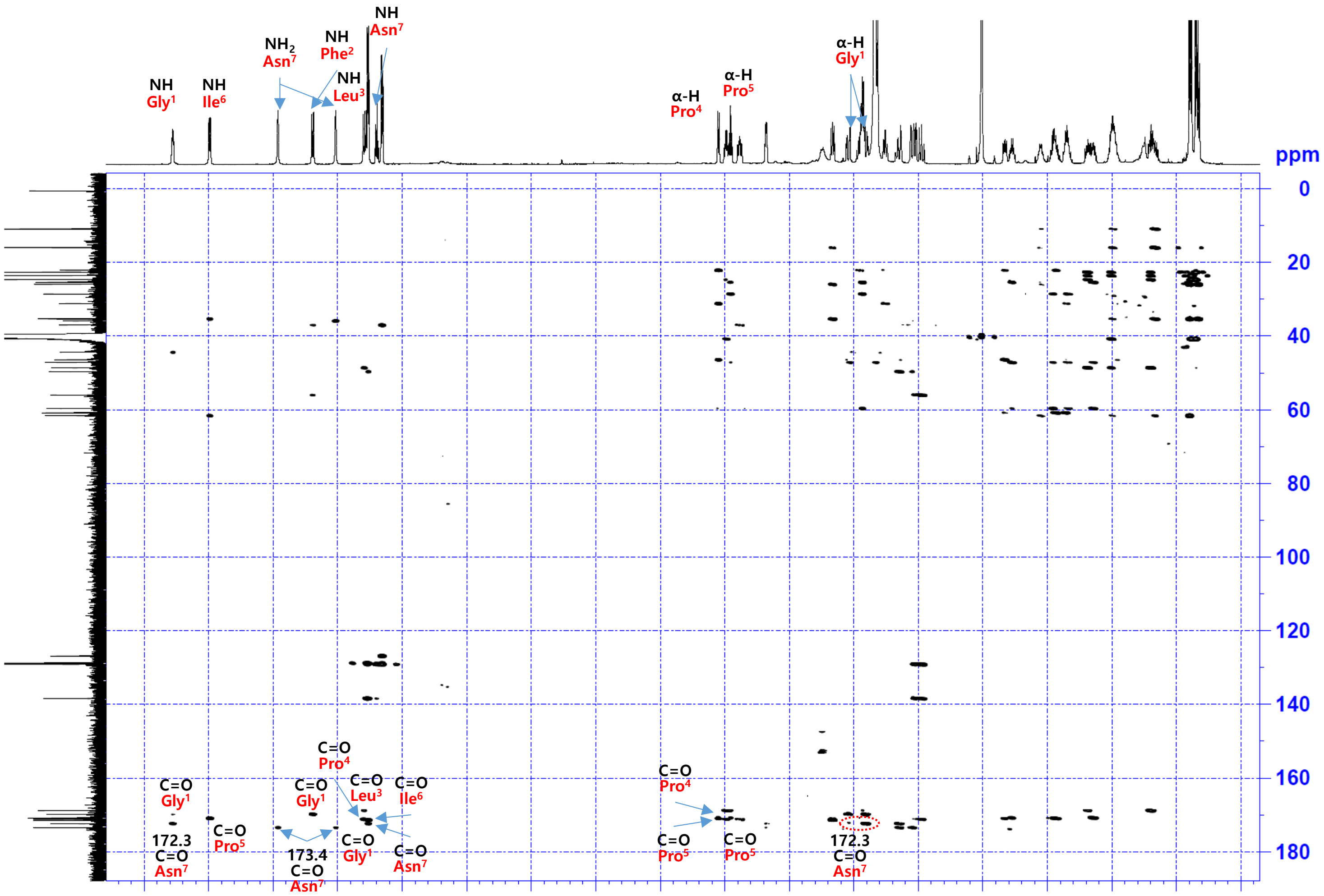

Figure S5. HMBC spectrum of compound **1**

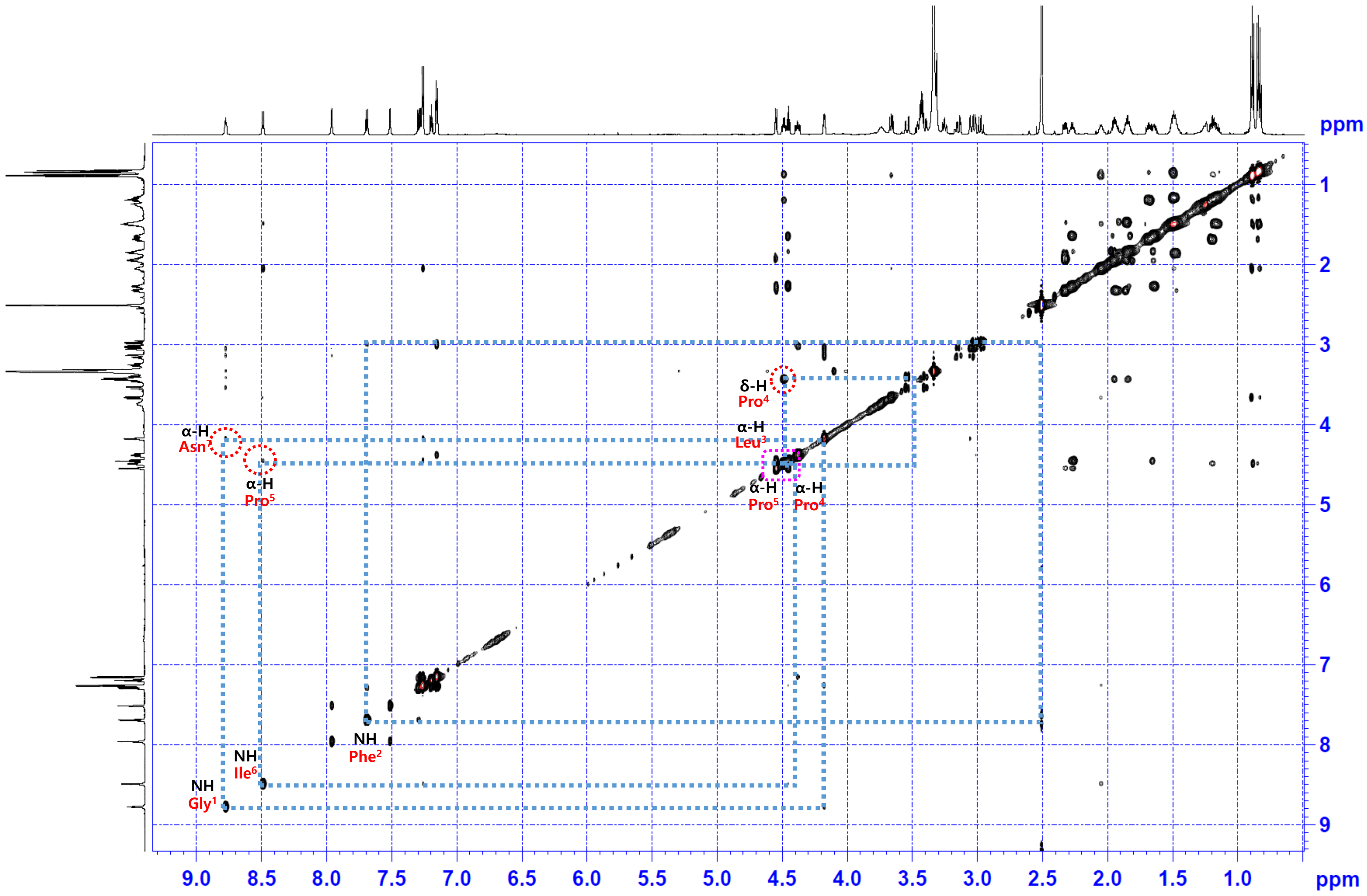

Figure S6. ROESY spectrum of compound **1**

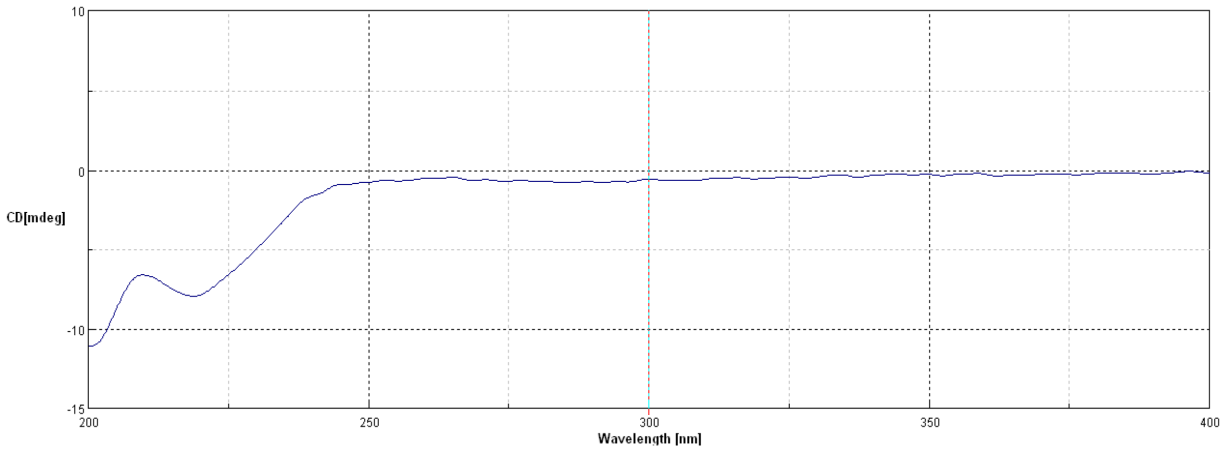

Figure S7. ECD spectrum of compound **1**

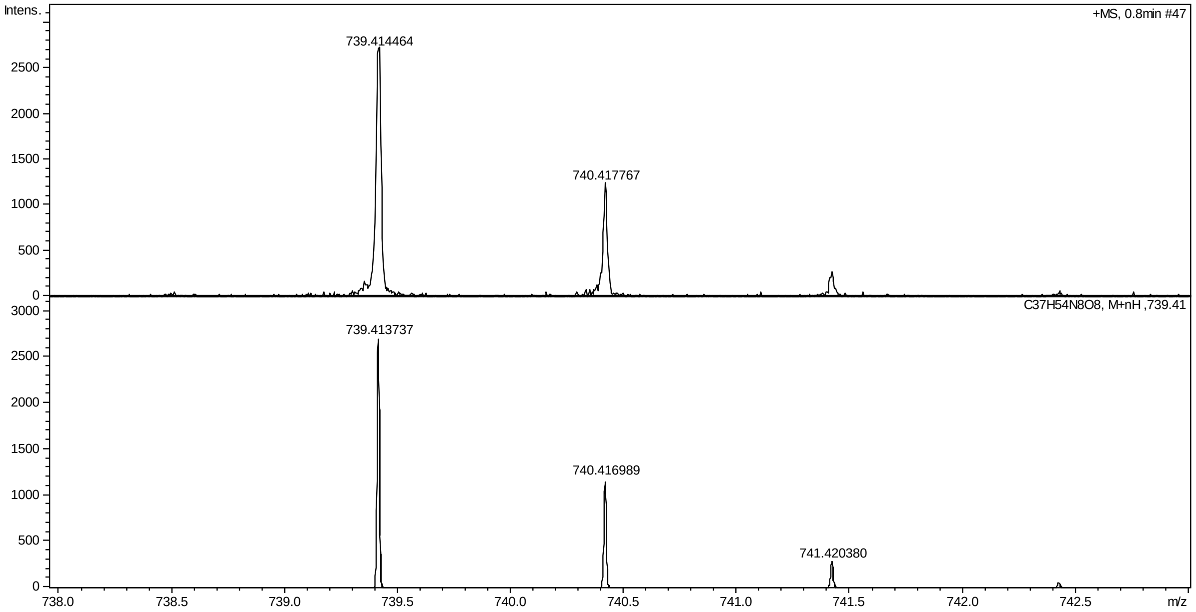

Figure S8. HRESI-MS spectrum of compound **1**

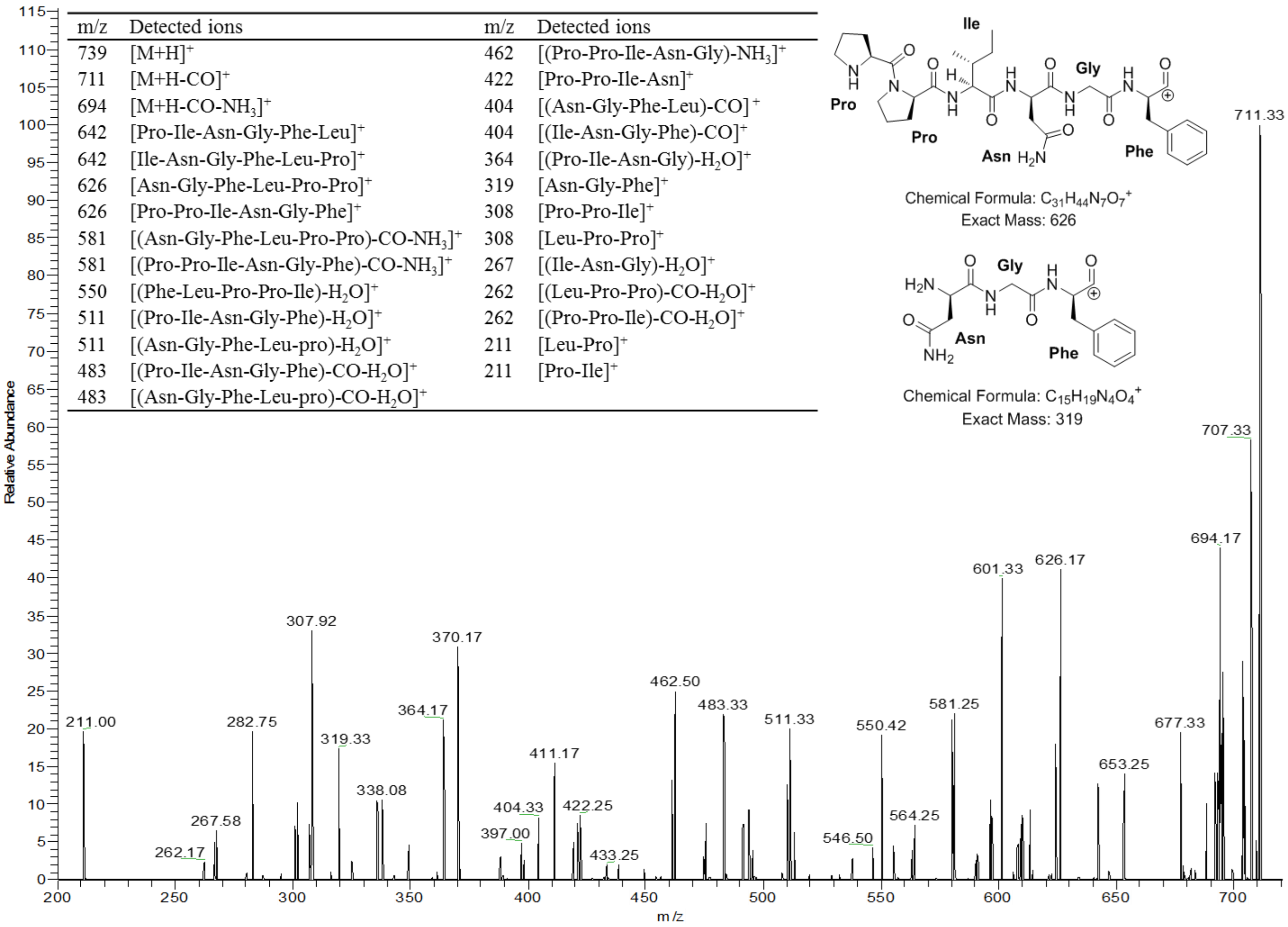

m/z	Detected ions	m/z	Detected ions
739	$[M+H]^+$	462	$[(Pro-Pro-Ile-Asn-Gly)-NH_3]^+$
711	$[M+H-CO]^+$	422	$[Pro-Pro-Ile-Asn]^+$
694	$[M+H-CO-NH_3]^+$	404	$[(Asn-Gly-Phe-Leu)-CO]^+$
642	$[Pro-Ile-Asn-Gly-Phe-Leu]^+$	404	$[(Ile-Asn-Gly-Phe)-CO]^+$
642	$[Ile-Asn-Gly-Phe-Leu-Pro]^+$	364	$[(Pro-Ile-Asn-Gly)-H_2O]^+$
626	$[Asn-Gly-Phe-Leu-Pro-Pro]^+$	319	$[Asn-Gly-Phe]^+$
626	$[Pro-Pro-Ile-Asn-Gly-Phe]^+$	308	$[Pro-Pro-Ile]^+$
581	$[(Asn-Gly-Phe-Leu-Pro-Pro)-CO-NH_3]^+$	308	$[Leu-Pro-Pro]^+$
581	$[(Pro-Pro-Ile-Asn-Gly-Phe)-CO-NH_3]^+$	267	$[(Ile-Asn-Gly)-H_2O]^+$
550	$[(Phe-Leu-Pro-Pro-Ile)-H_2O]^+$	262	$[(Leu-Pro-Pro)-CO-H_2O]^+$
511	$[(Pro-Ile-Asn-Gly-Phe)-H_2O]^+$	262	$[(Pro-Pro-Ile)-CO-H_2O]^+$
511	$[(Asn-Gly-Phe-Leu-pro)-H_2O]^+$	211	$[Leu-Pro]^+$
483	$[(Pro-Ile-Asn-Gly-Phe)-CO-H_2O]^+$	211	$[Pro-Ile]^+$
483	$[(Asn-Gly-Phe-Leu-pro)-CO-H_2O]^+$		

Figure S9. ESI-MS/MS spectrum of compound **1**

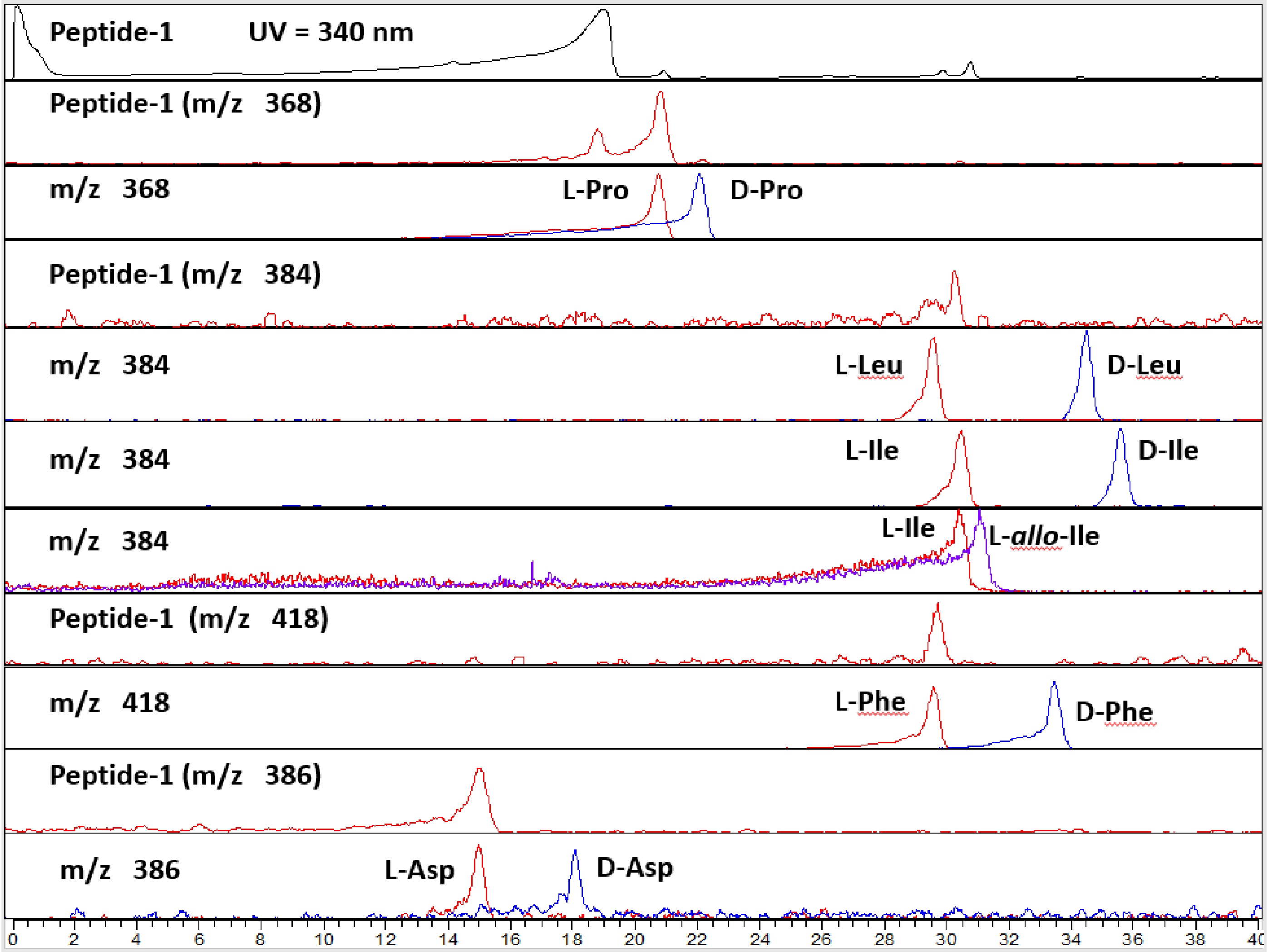

Figure S10. Marfey's analysis of compound **1**

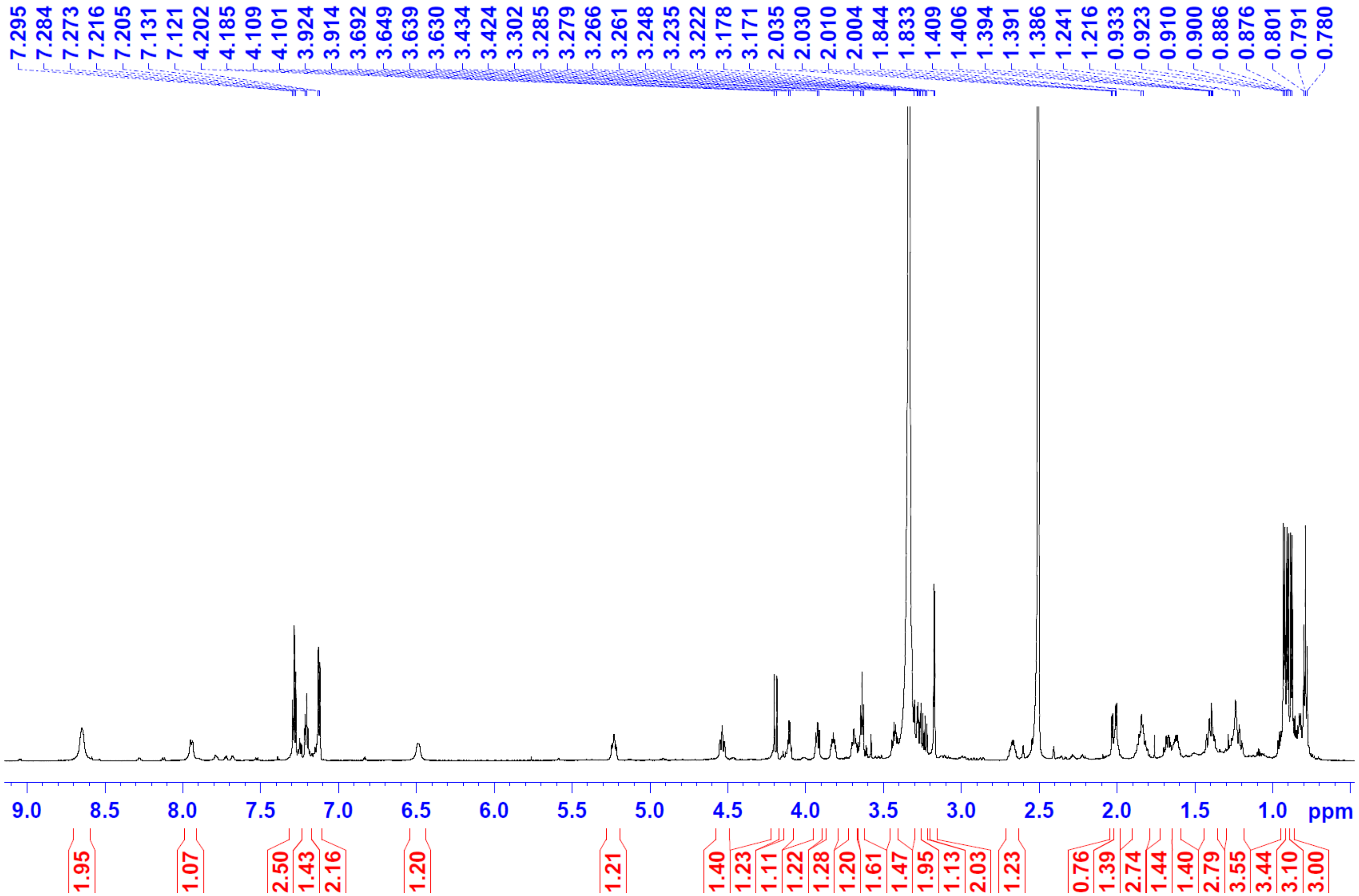

Figure S11. ^{1}H NMR spectrum of compound **2** (DMSO-d_6, 700 MHz)

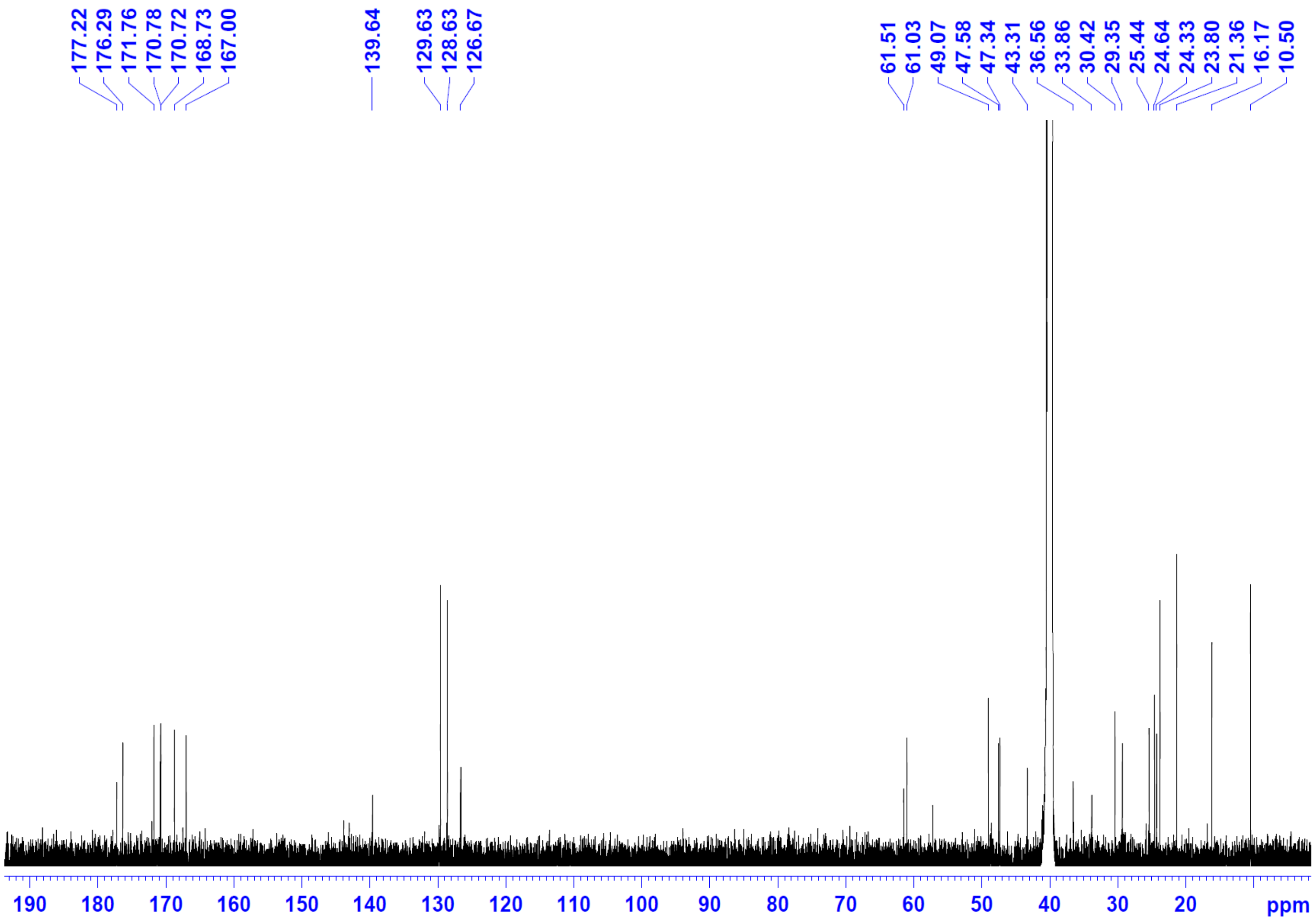

Figure S12. ^{13}C NMR spectrum of compound **2** (DMSO-d_6, 175 MHz)

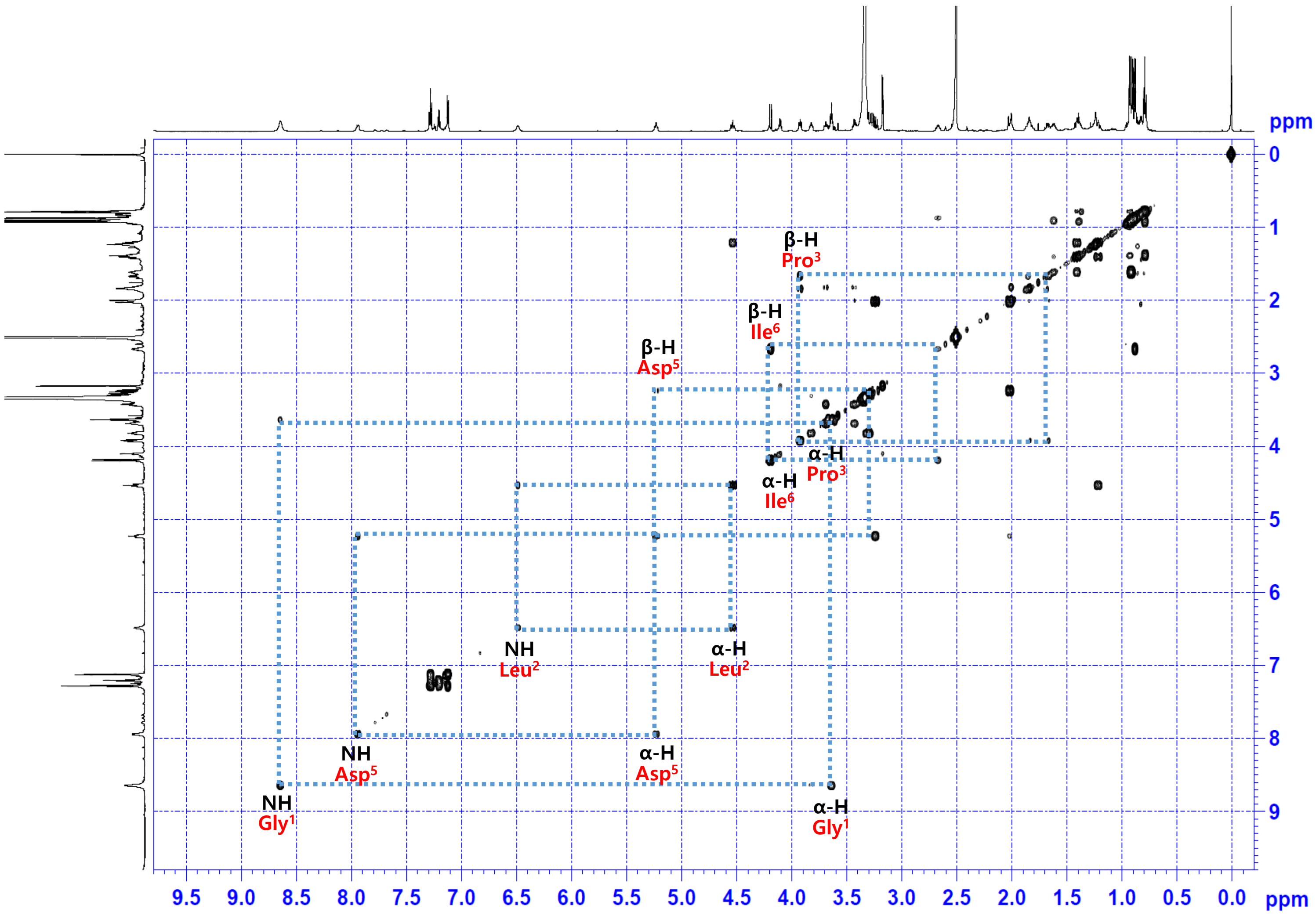

Figure S13. COSY spectrum of compound **2**

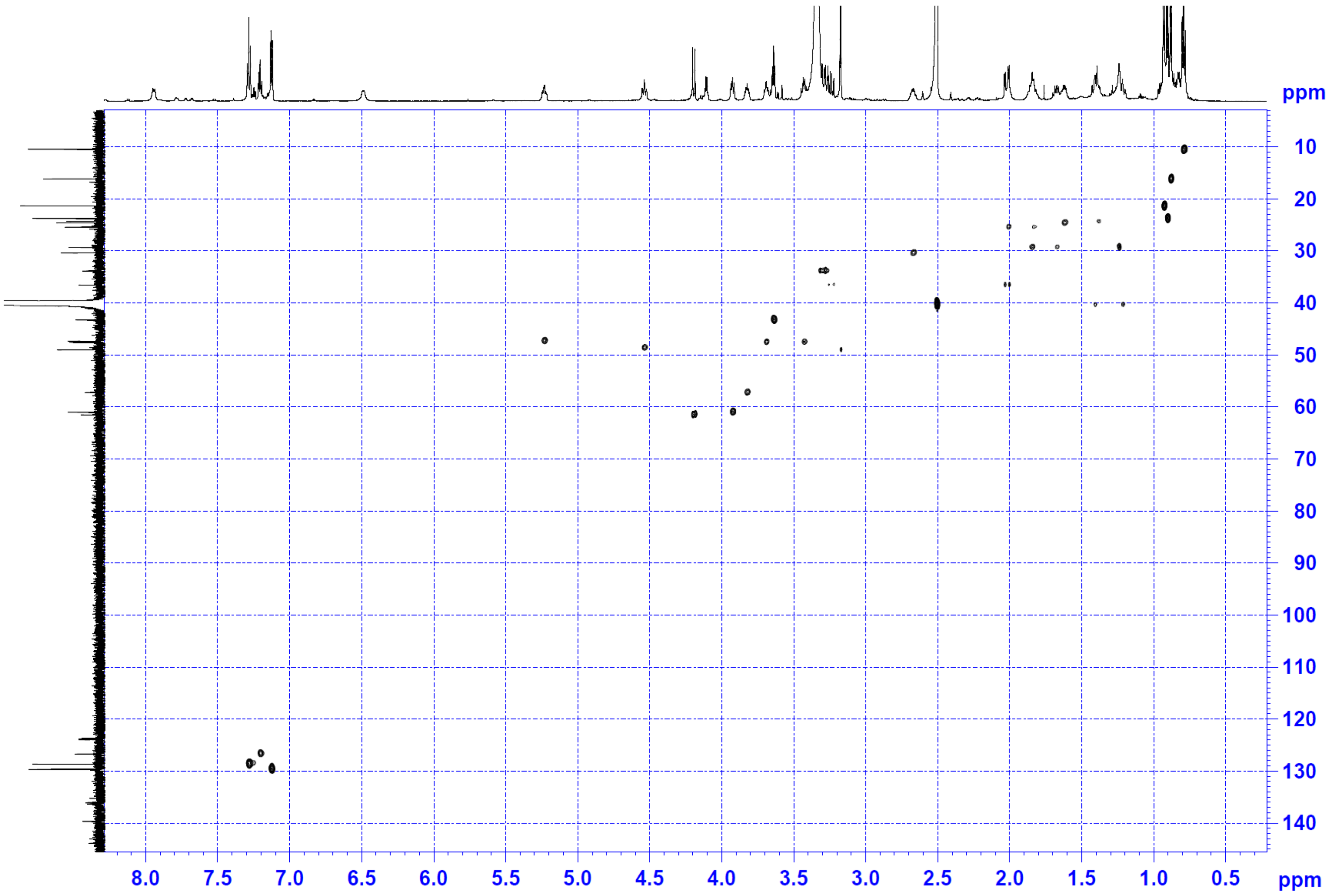

Figure S14. HSQC spectrum of compound **2**

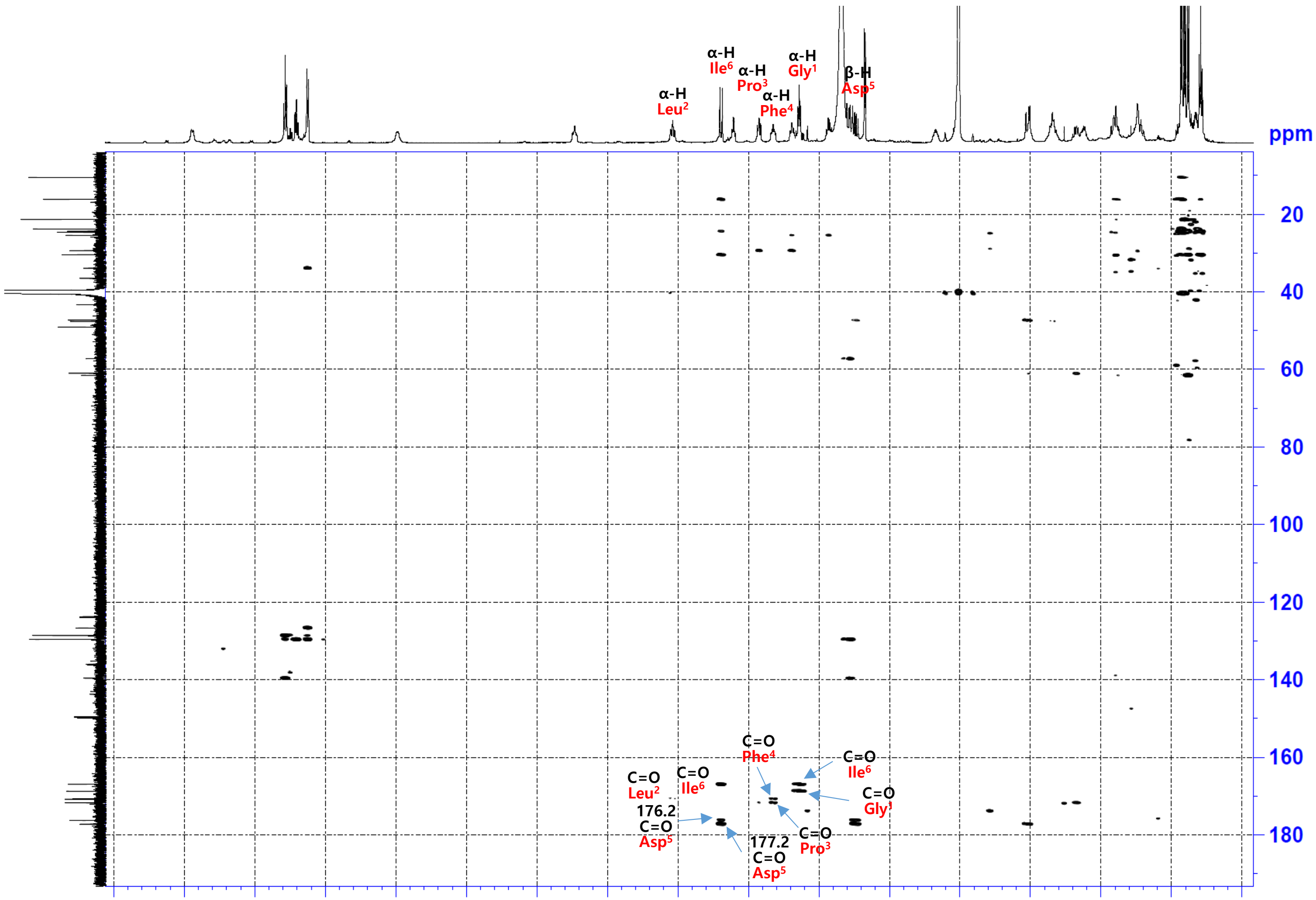

Figure S15. HMBC spectrum of compound **2**

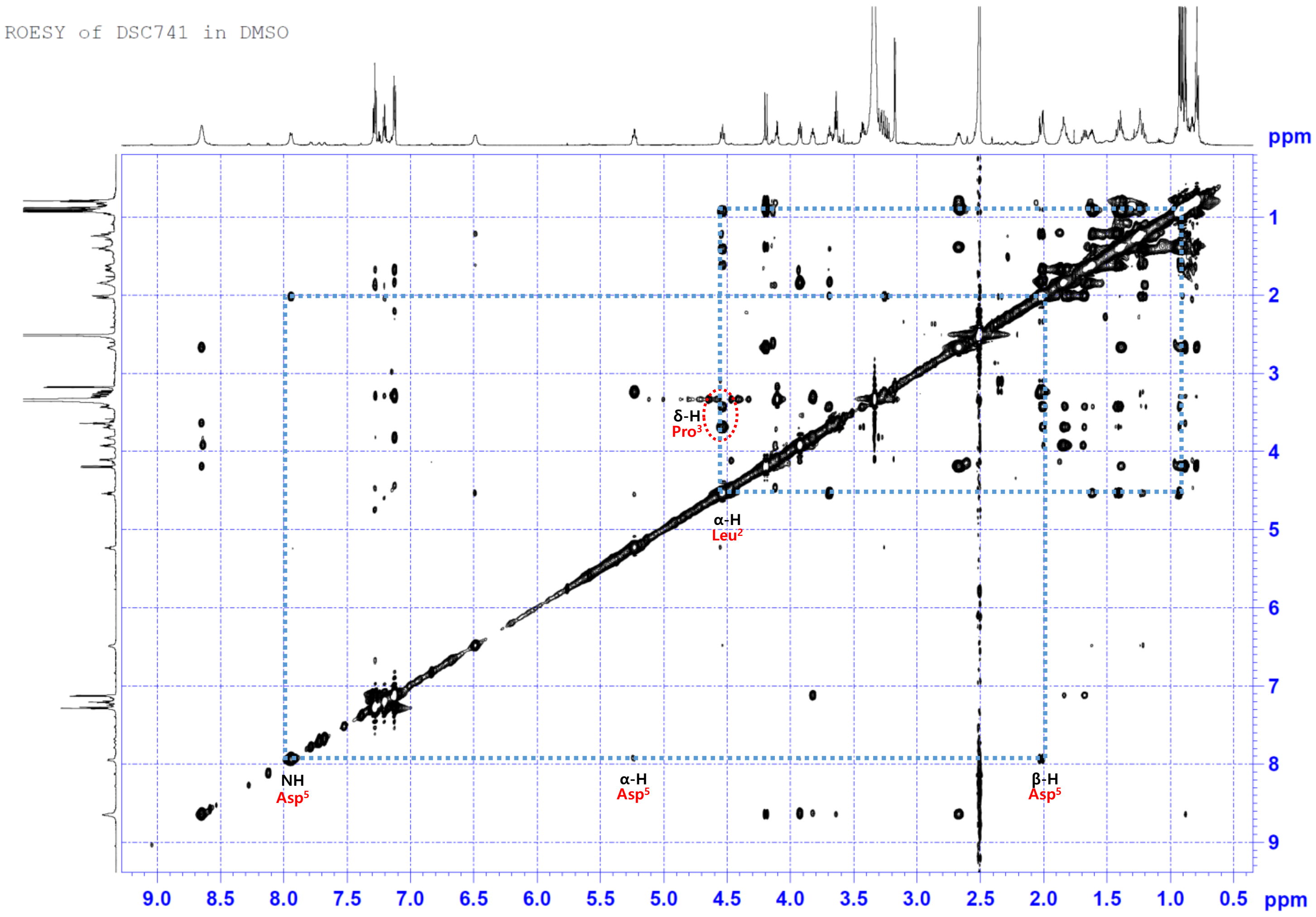

Figure S16. ROESY spectrum of compound **2**

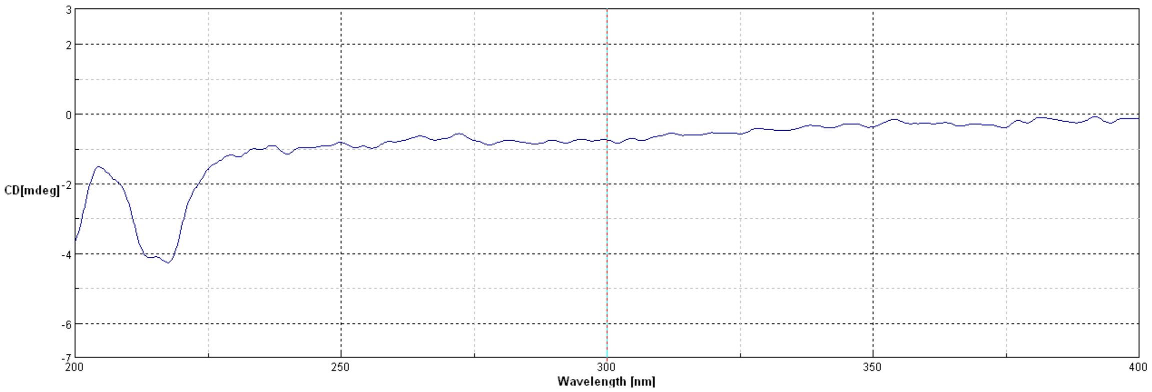

Figure S17. ECD spectrum of compound **2**

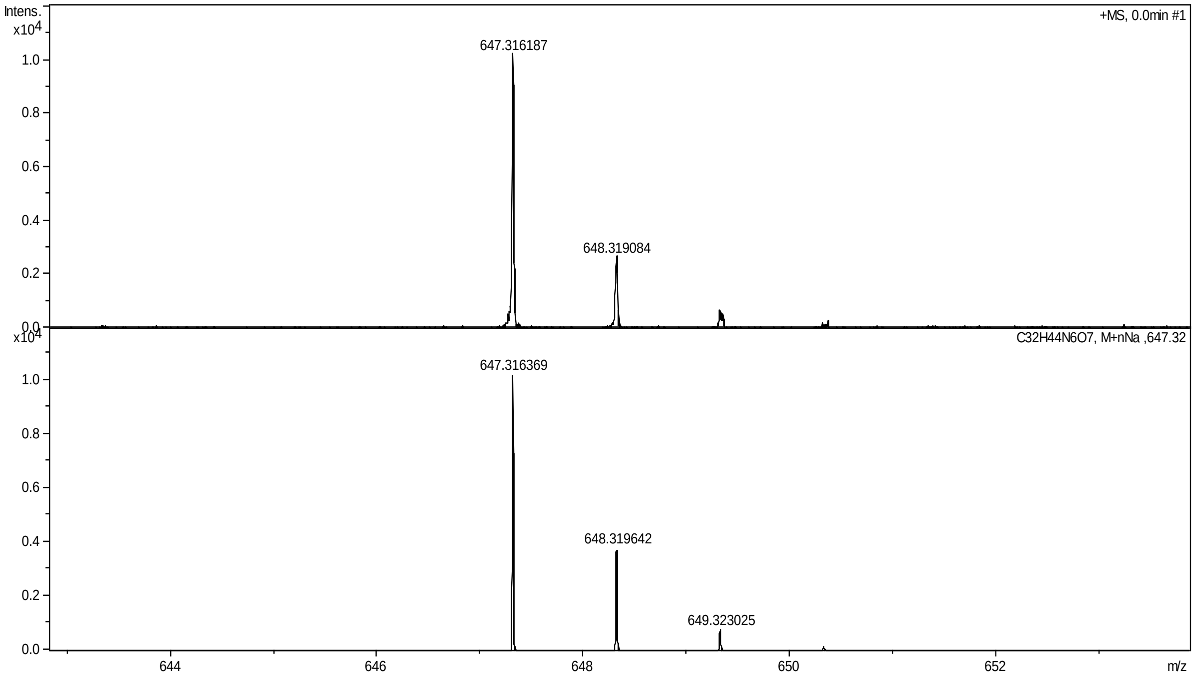

Figure S18. HRESI-MS spectrum of compound **2**

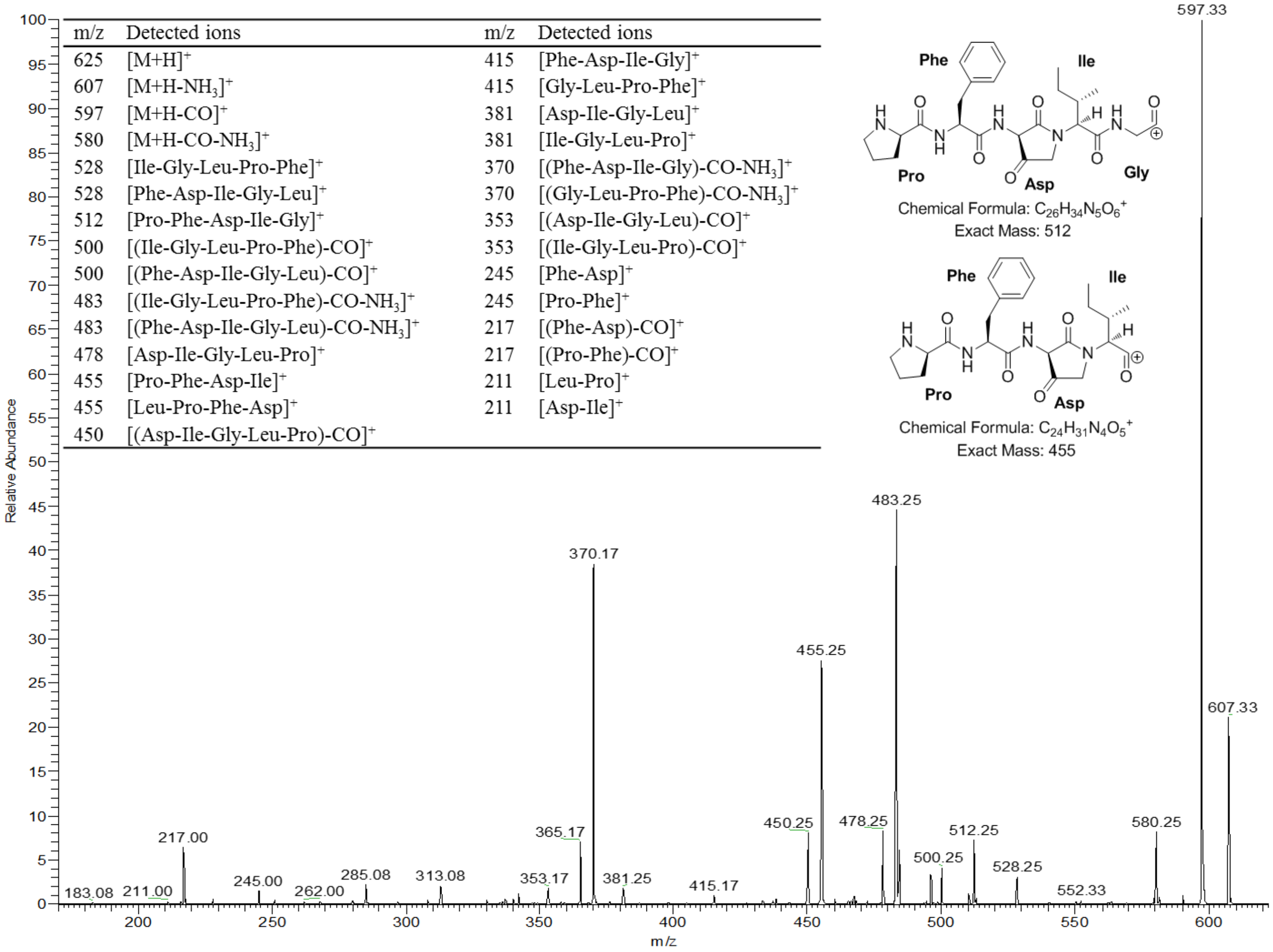

m/z	Detected ions	m/z	Detected ions
625	$[M+H]^+$	415	[Phe-Asp-Ile-Gly]$^+$
607	$[M+H-NH_3]^+$	415	[Gly-Leu-Pro-Phe]$^+$
597	$[M+H-CO]^+$	381	[Asp-Ile-Gly-Leu]$^+$
580	$[M+H-CO-NH_3]^+$	381	[Ile-Gly-Leu-Pro]$^+$
528	[Ile-Gly-Leu-Pro-Phe]$^+$	370	[(Phe-Asp-Ile-Gly)-CO-NH_3]$^+$
528	[Phe-Asp-Ile-Gly-Leu]$^+$	370	[(Gly-Leu-Pro-Phe)-CO-NH_3]$^+$
512	[Pro-Phe-Asp-Ile-Gly]$^+$	353	[(Asp-Ile-Gly-Leu)-CO]$^+$
500	[(Ile-Gly-Leu-Pro-Phe)-CO]$^+$	353	[(Ile-Gly-Leu-Pro)-CO]$^+$
500	[(Phe-Asp-Ile-Gly-Leu)-CO]$^+$	245	[Phe-Asp]$^+$
483	[(Ile-Gly-Leu-Pro-Phe)-CO-NH_3]$^+$	245	[Pro-Phe]$^+$
483	[(Phe-Asp-Ile-Gly-Leu)-CO-NH_3]$^+$	217	[(Phe-Asp)-CO]$^+$
478	[Asp-Ile-Gly-Leu-Pro]$^+$	217	[(Pro-Phe)-CO]$^+$
455	[Pro-Phe-Asp-Ile]$^+$	211	[Leu-Pro]$^+$
455	[Leu-Pro-Phe-Asp]$^+$	211	[Asp-Ile]$^+$
450	[(Asp-Ile-Gly-Leu-Pro)-CO]$^+$		

Figure S19. ESI-MS/MS spectrum of compound **2**

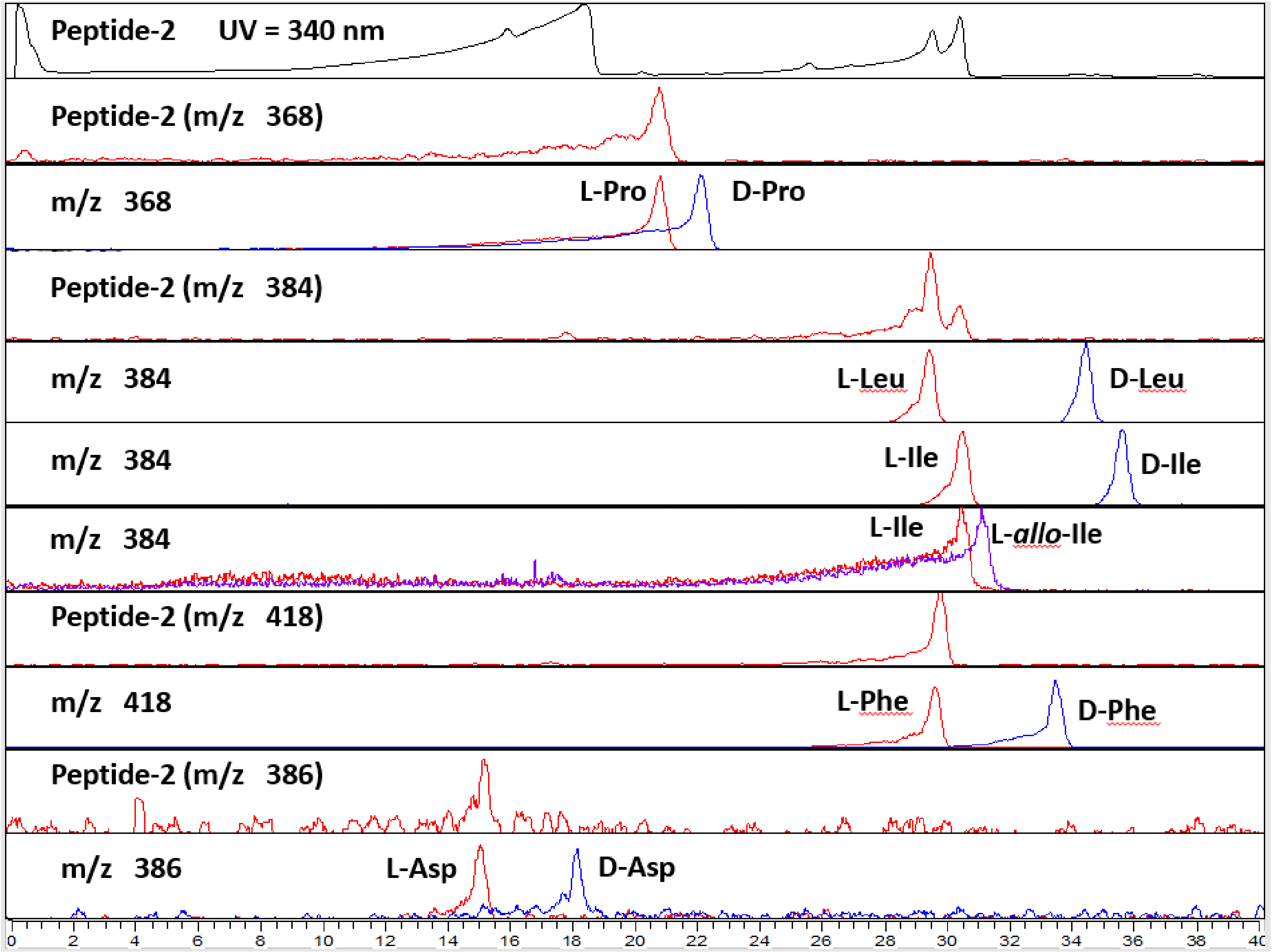

Figure S20. Marfey's analysis of compound **2**

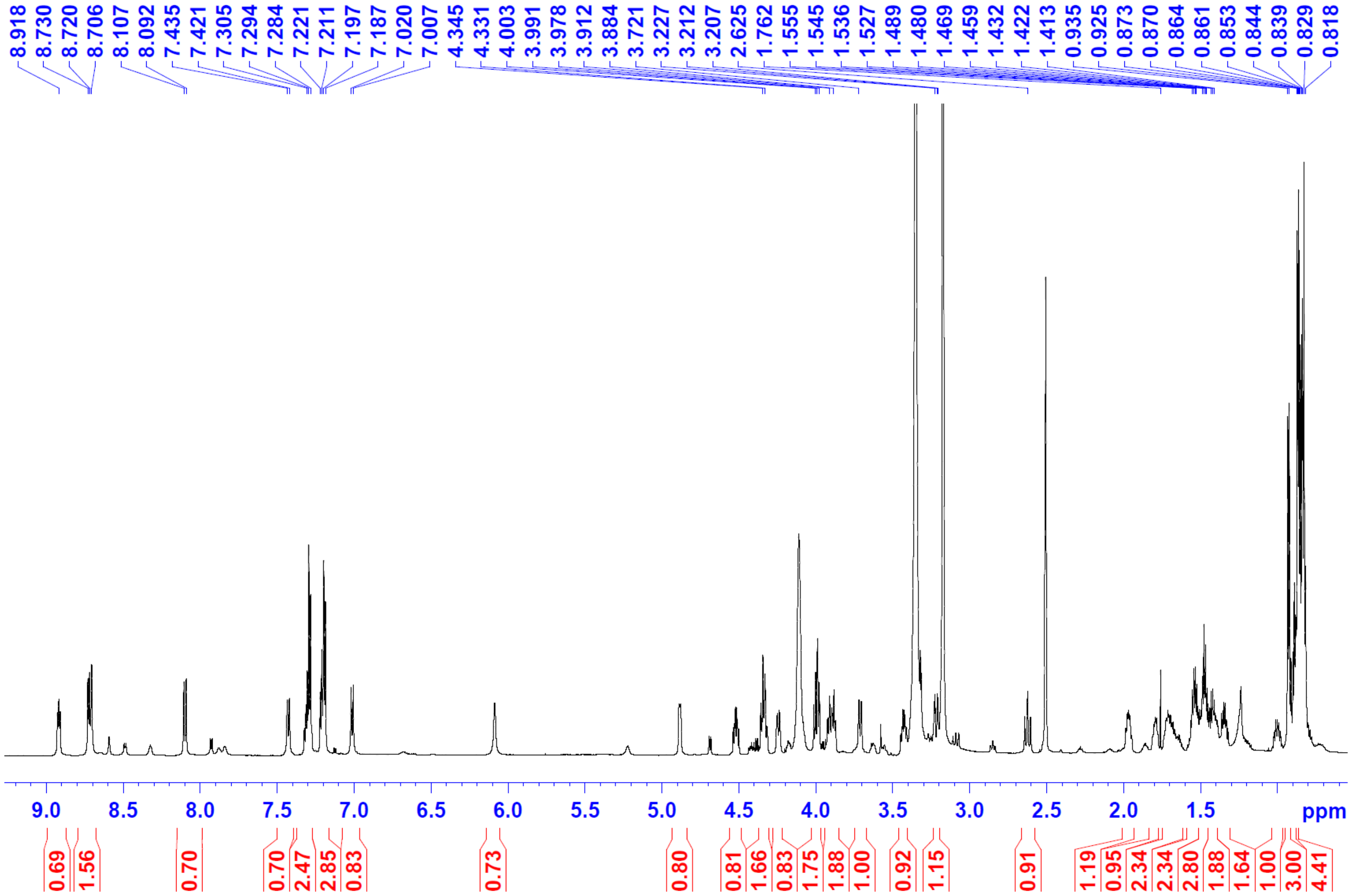

Figure S21. ^{1}H NMR spectrum of compound **3** (DMSO-d_6, 700 MHz)

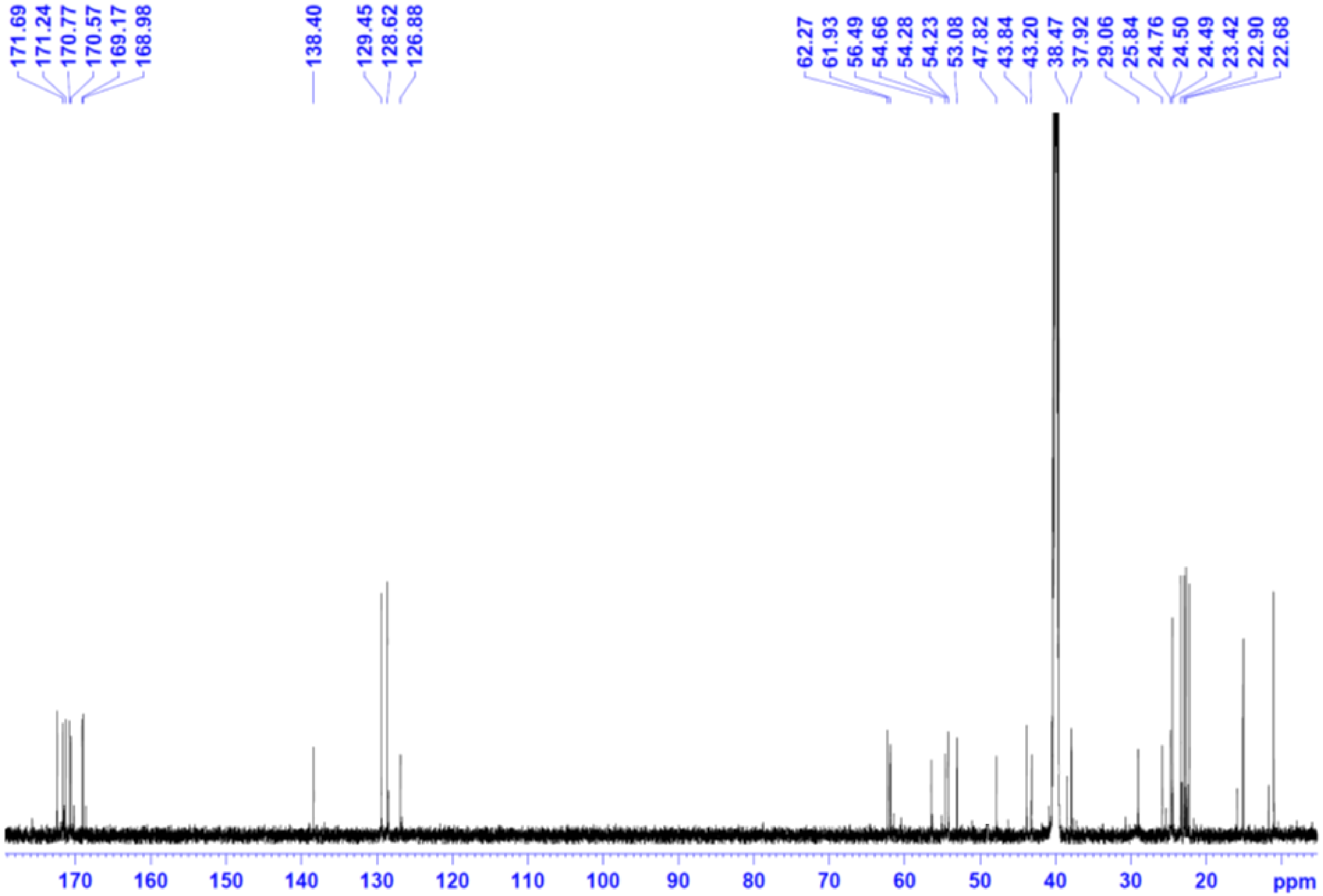

Figure S22. ^{13}C NMR spectrum of compound **3** (DMSO-d_6, 175 MHz)

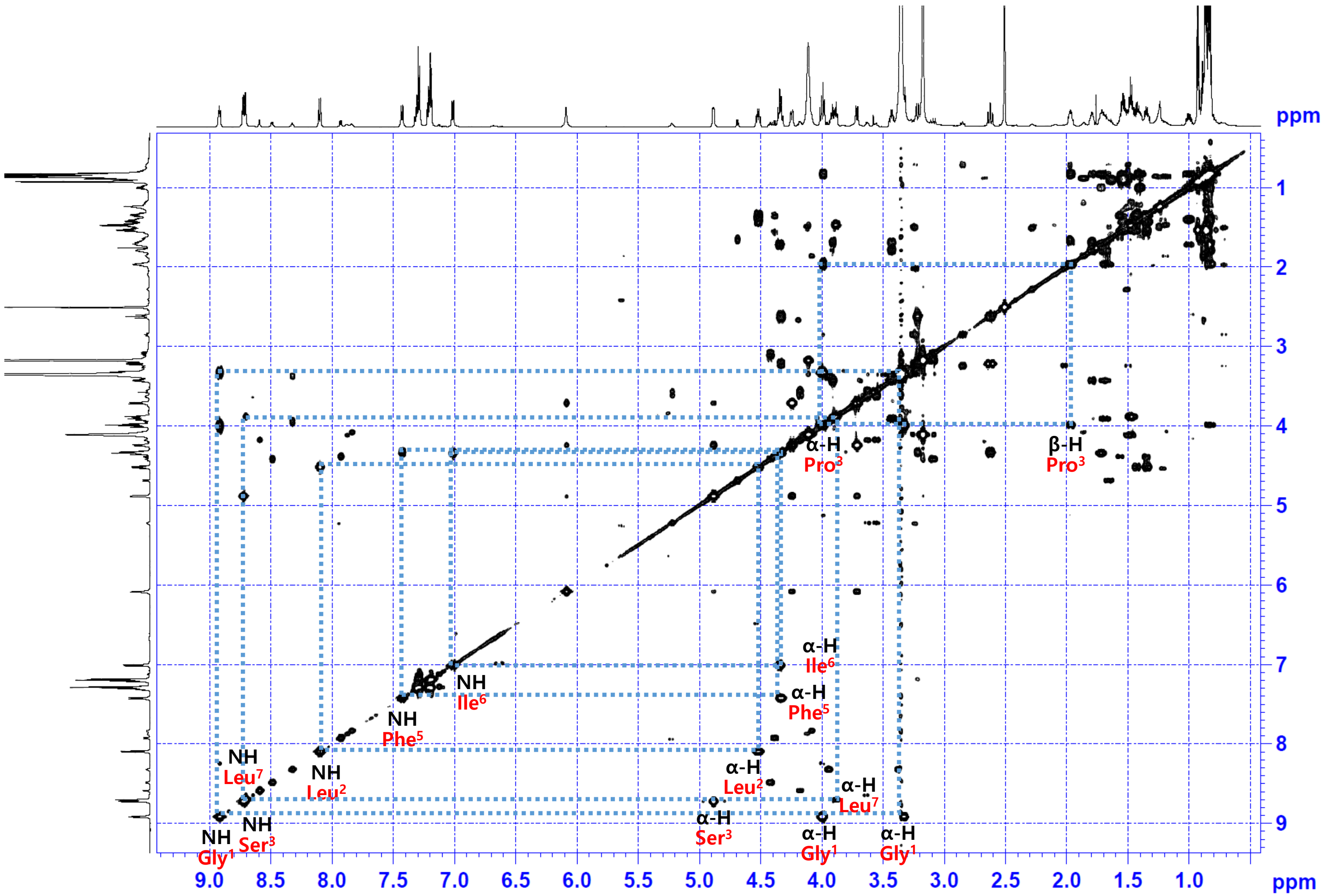

Figure S23. COSY spectrum of compound **3**

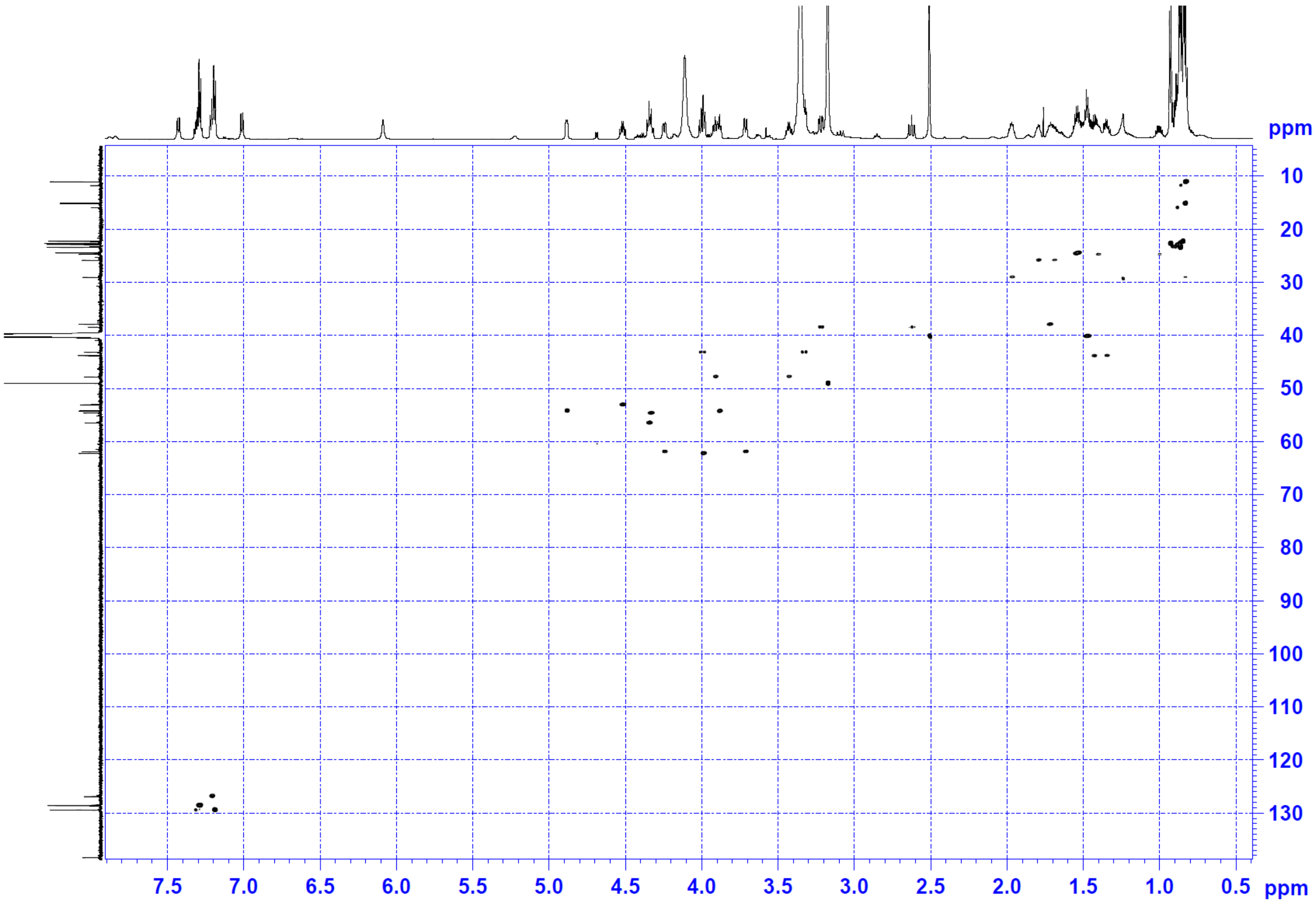

Figure S24. HSQC spectrum of compound **3**

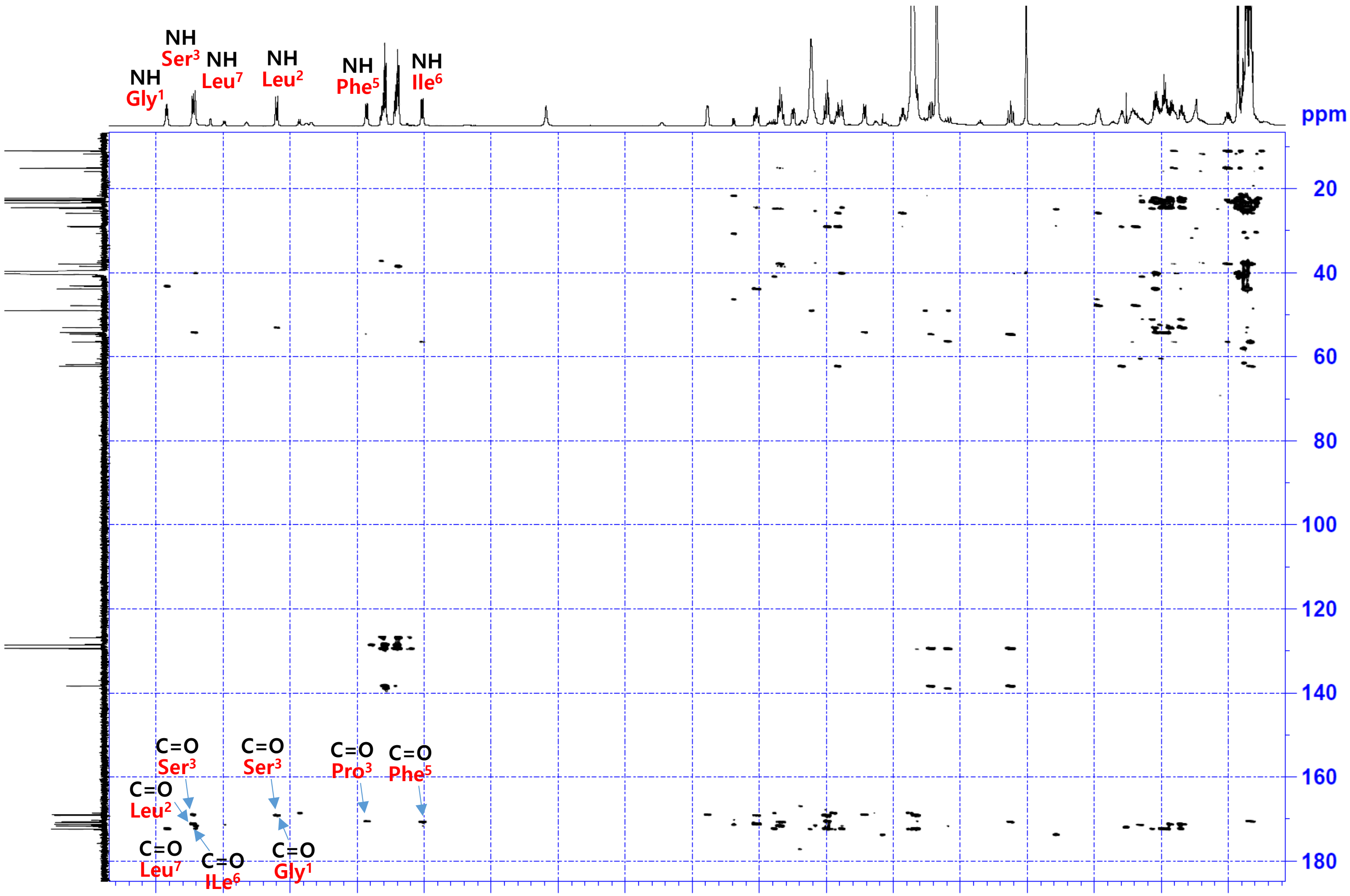

Figure S25. HMBC spectrum of compound **3**

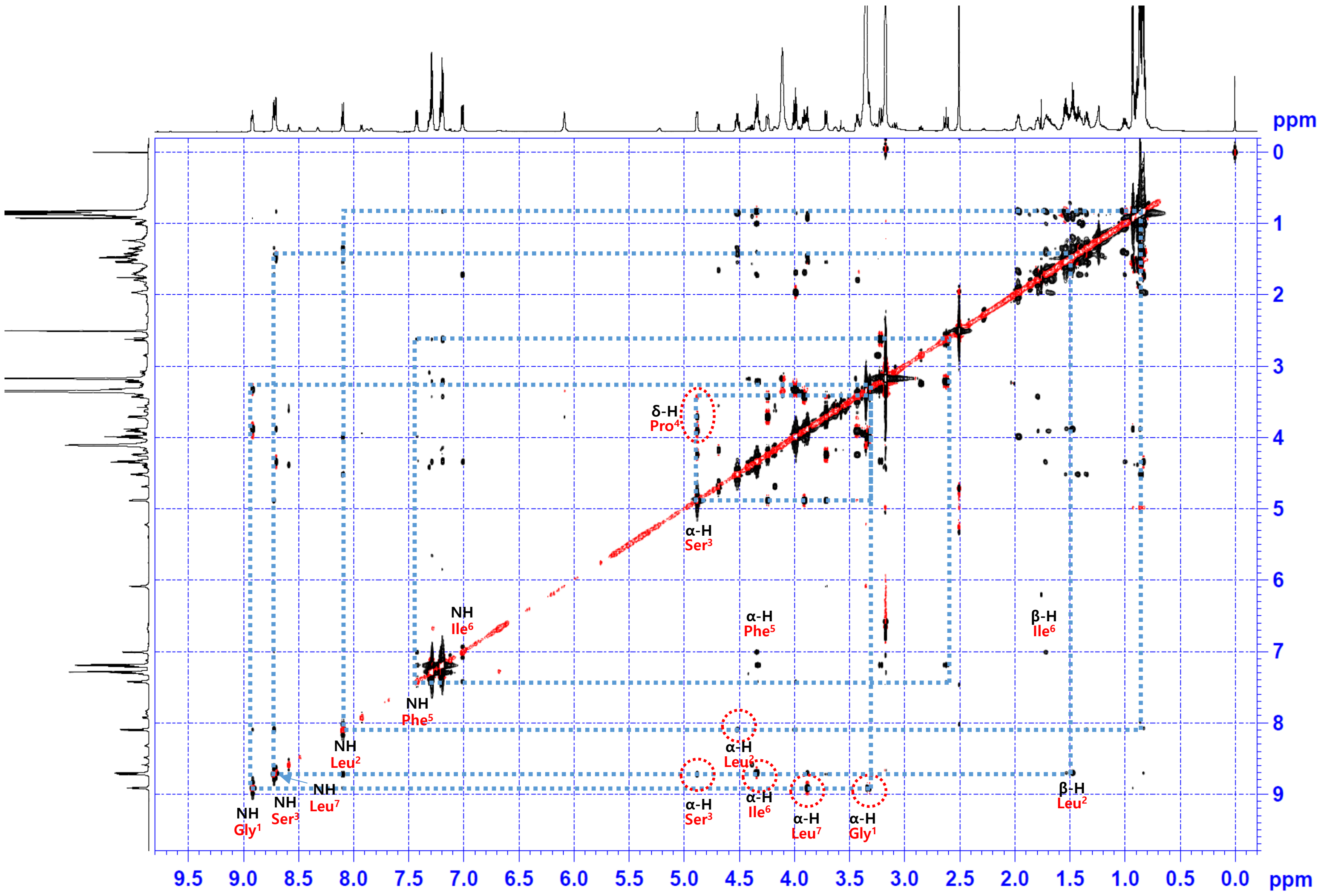

Figure S26. ROESY spectrum of compound **3**

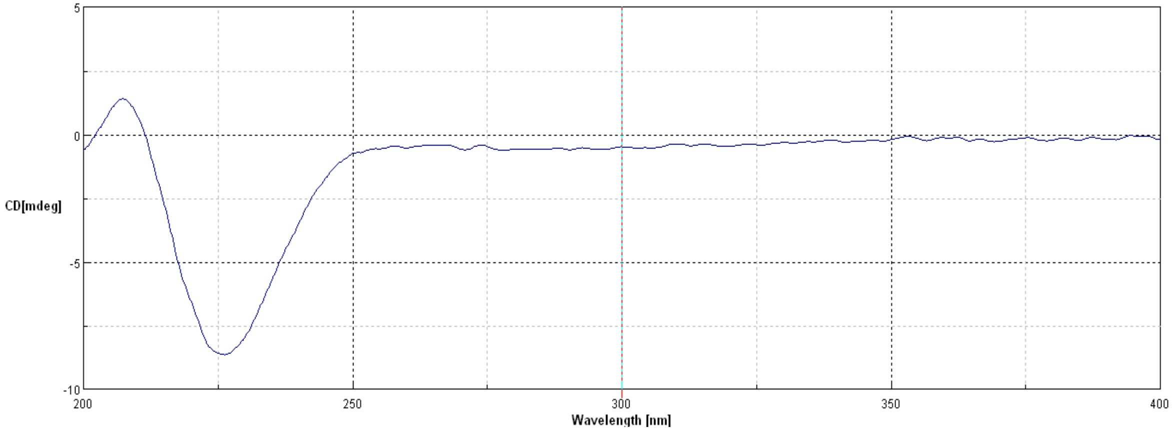

Figure S27. ECD spectrum of compound **3**

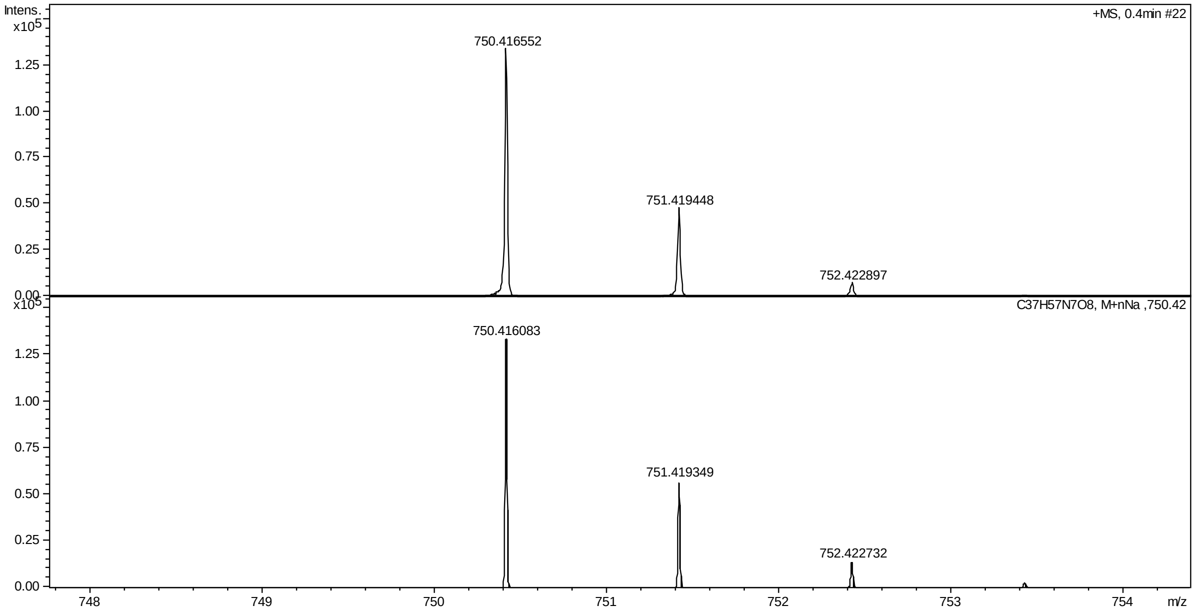

Figure S28. HRESI-MS spectrum of compound **3**

m/z	Detected ions	m/z	Detected ions
728	$[M+H]^+$	471	$[\text{Pro-Phe-Ile-Leu}]^+$
710	$[M+H\text{-}H_2O]^+$	358	$[\text{Pro-Phe-Ile}]^+$
693	$[M+H\text{-}NH_3\text{-}H_2O]^+$	337	$[(\text{Gly-Leu-Ser-Pro})\text{-}H_2O]^+$
641	$[\text{Pro-Phe-Ile-Leu-Gly-Leu}]^+$	329	$[(\text{Phe-Ile-Leu})\text{-CO-}NH_3]^+$
613	$[(\text{Pro-Phe-Ile-Leu-Gly-Leu})\text{-CO}]^+$	314	$[(\text{Ser-Pro-Phe})\text{-}H_2O]^+$
528	$[\text{Pro-Phe-Ile-Leu-Gly}]^+$	245	$[\text{Pro-Phe}]^+$

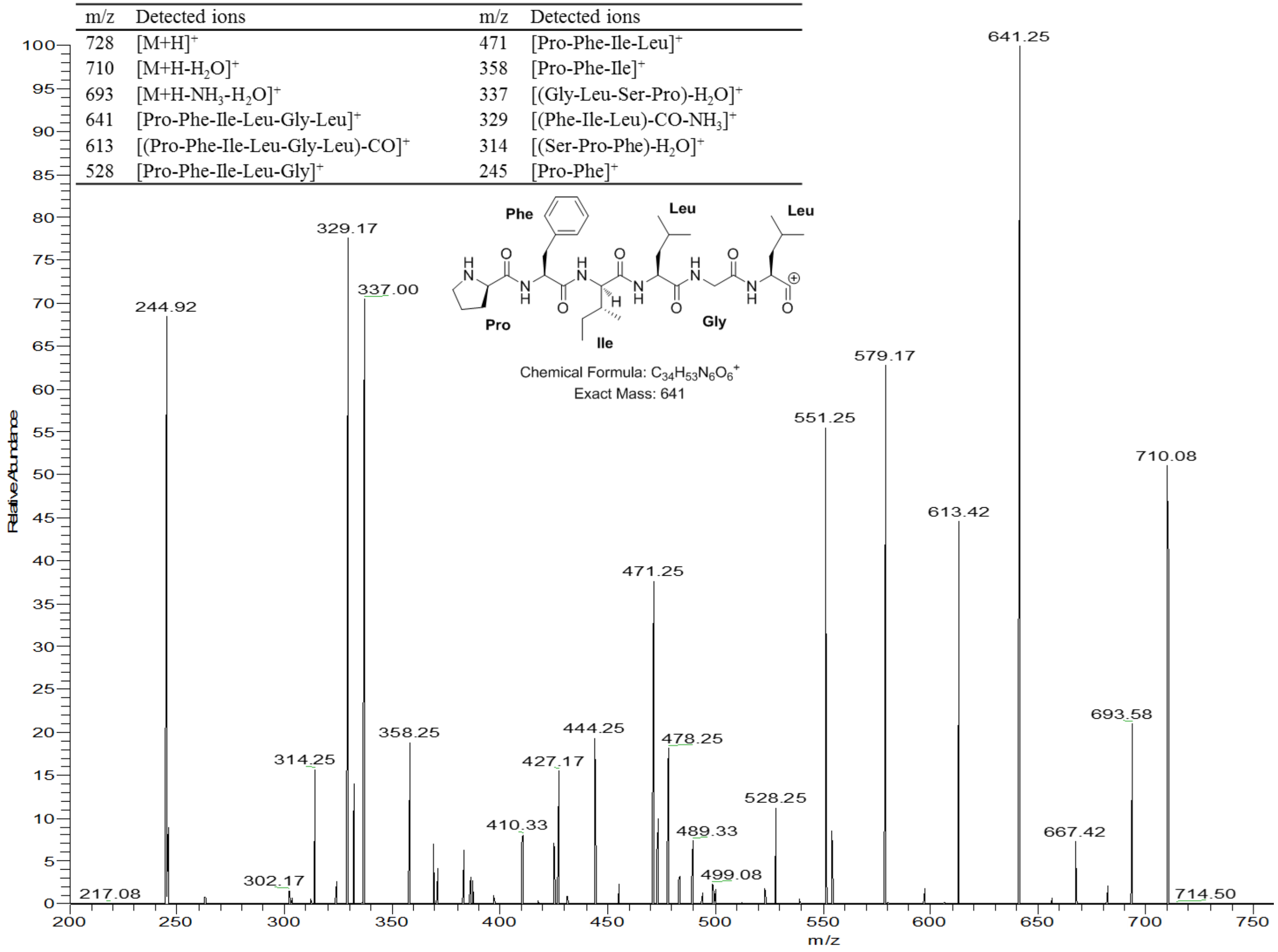

Figure S29. ESI-MS/MS spectrum of compound **3**

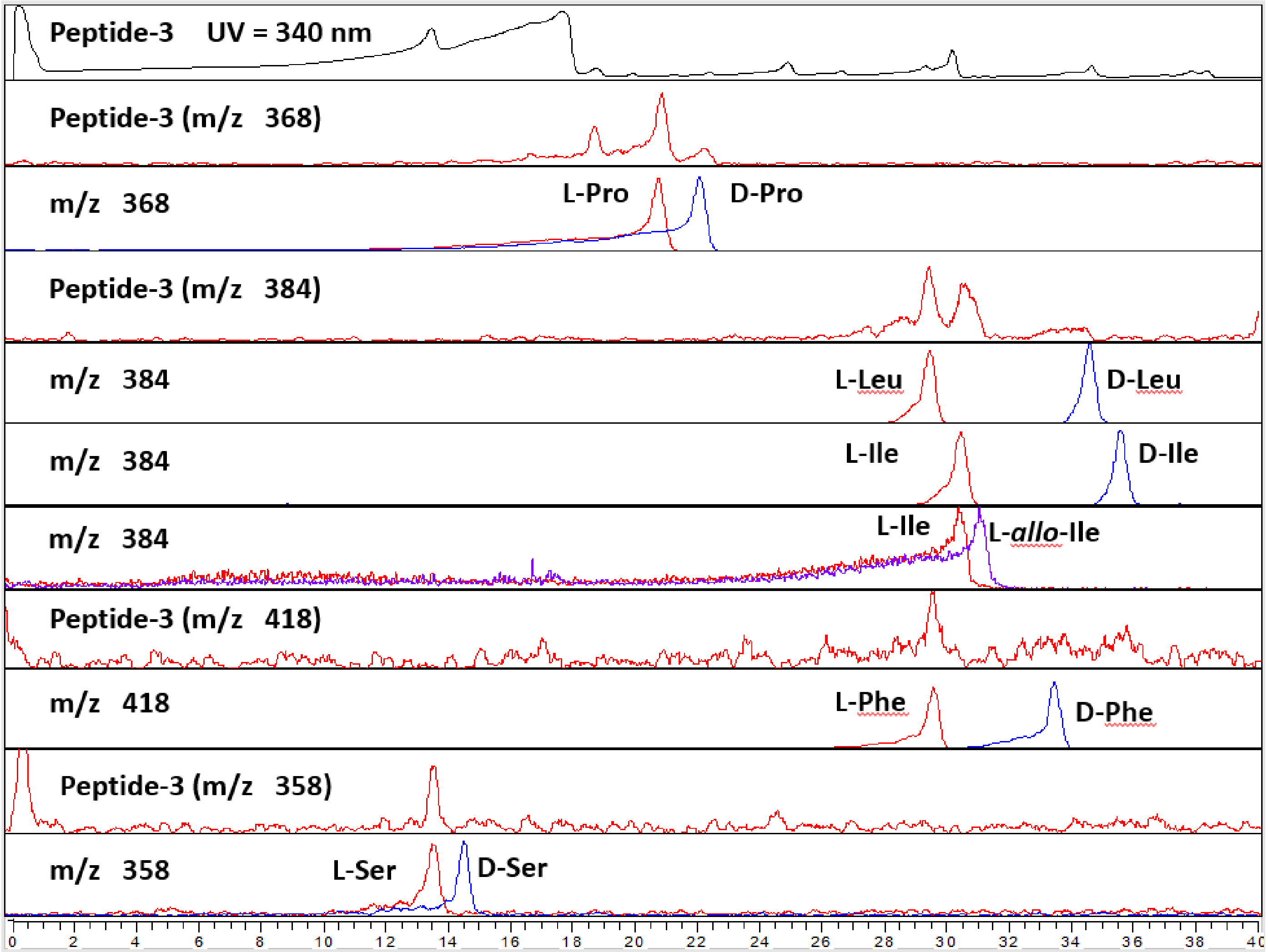

Figure S30. Marfey's analysis of compound **3**

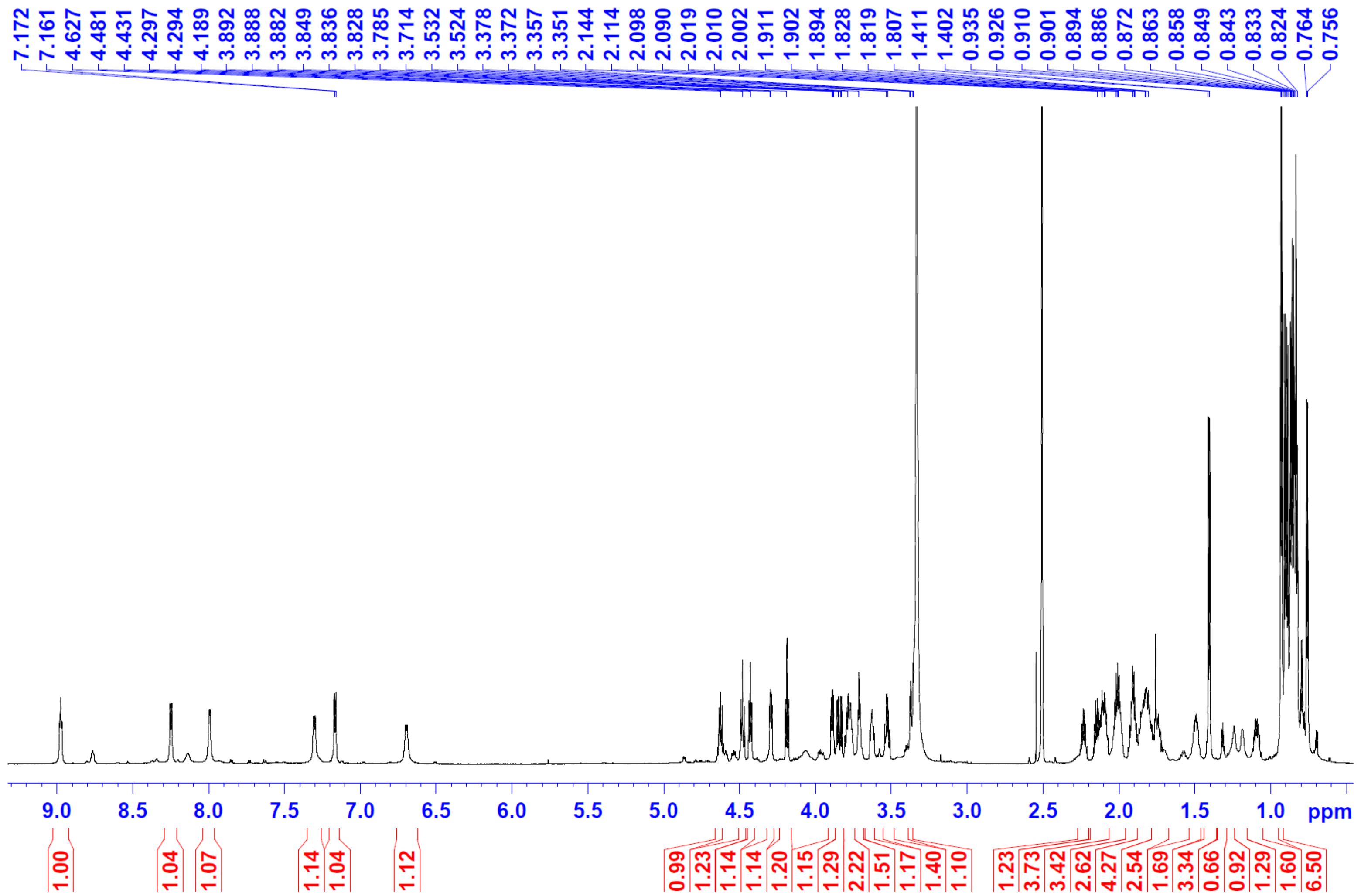

Figure S31. ^{1}H NMR spectrum of compound **4** (DMSO-d_6, 700 MHz)

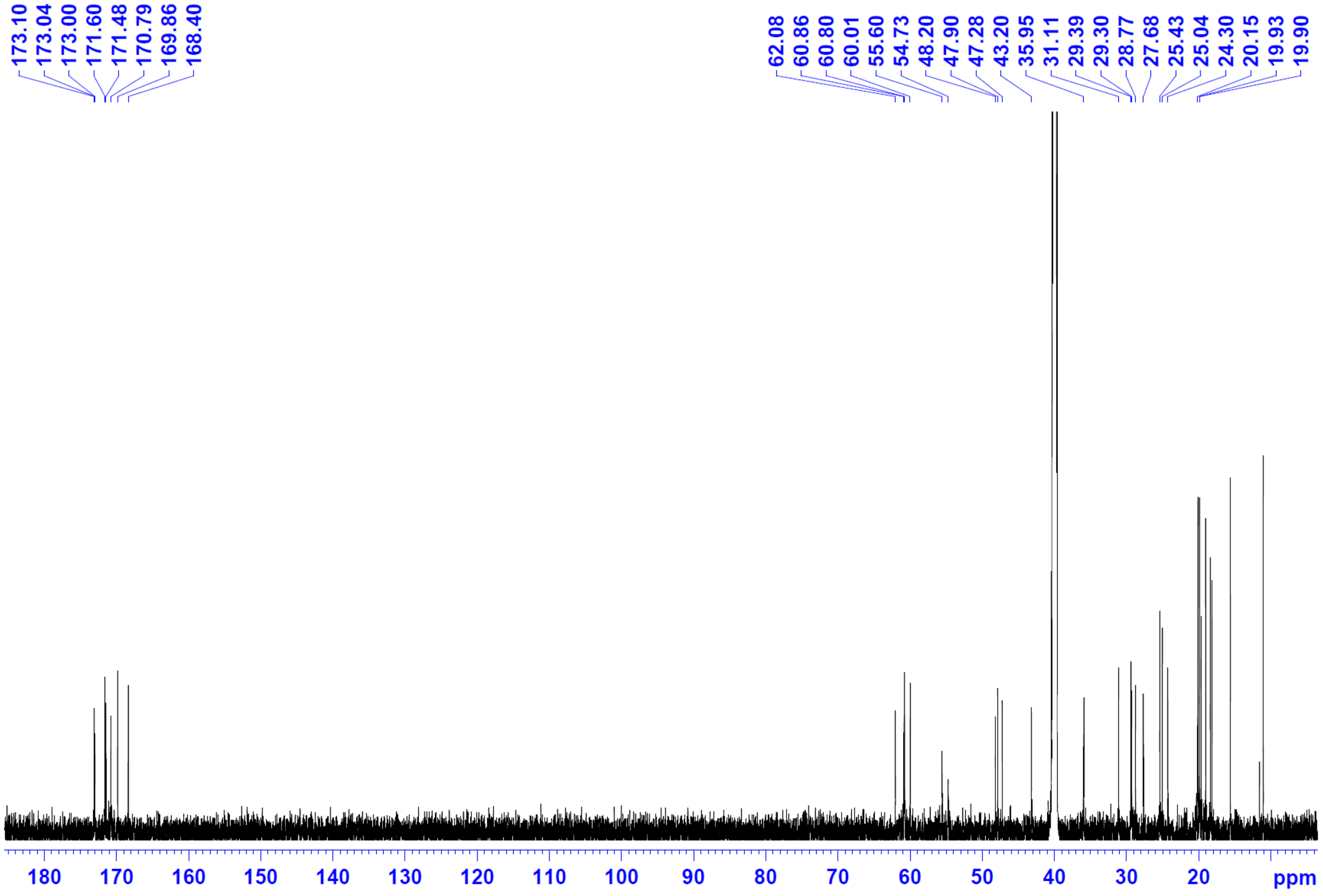

Figure S32. ^{13}C NMR spectrum of compound **4** (DMSO-d_6, 175 MHz)

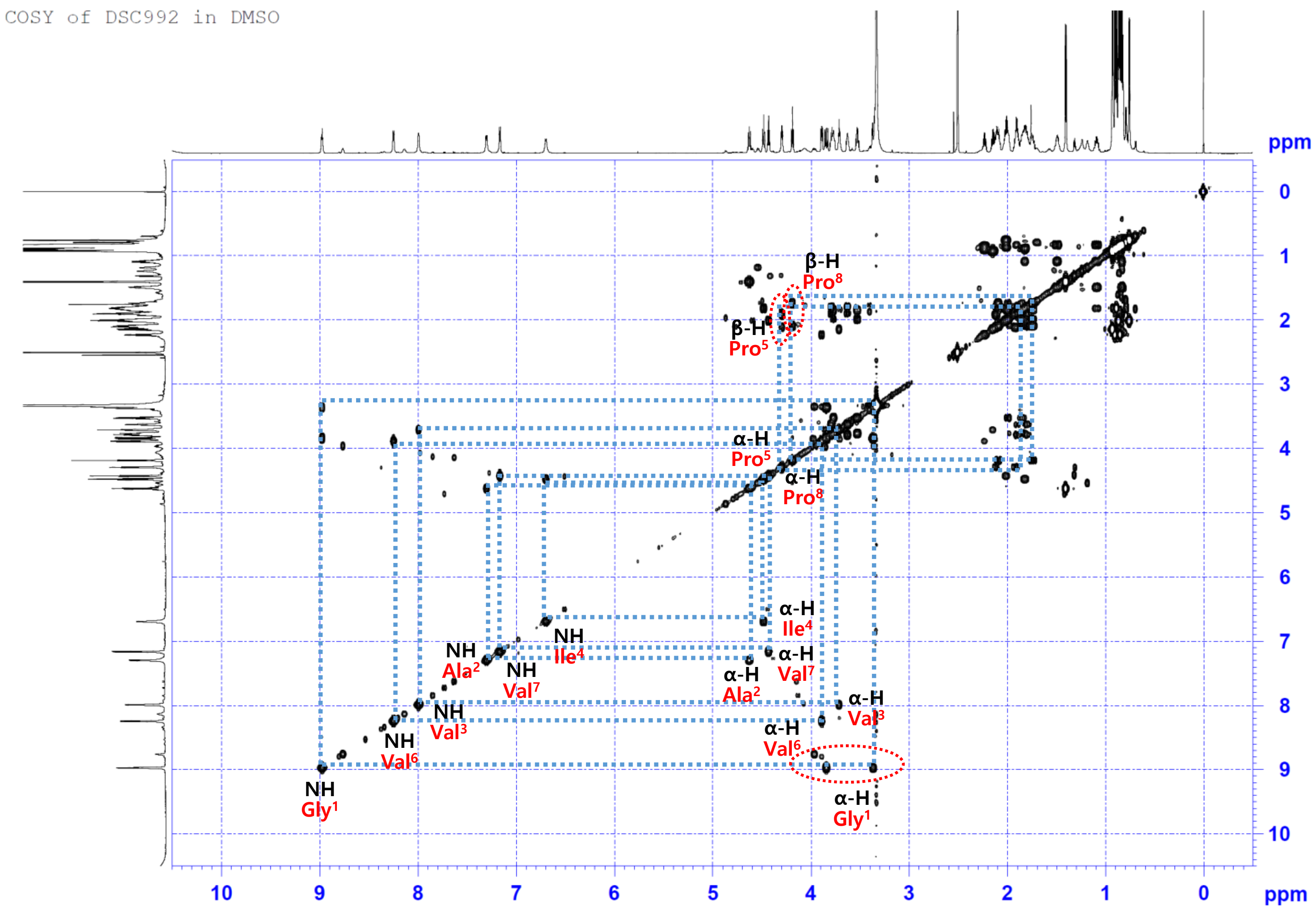

Figure S33. COSY spectrum of compound **4**

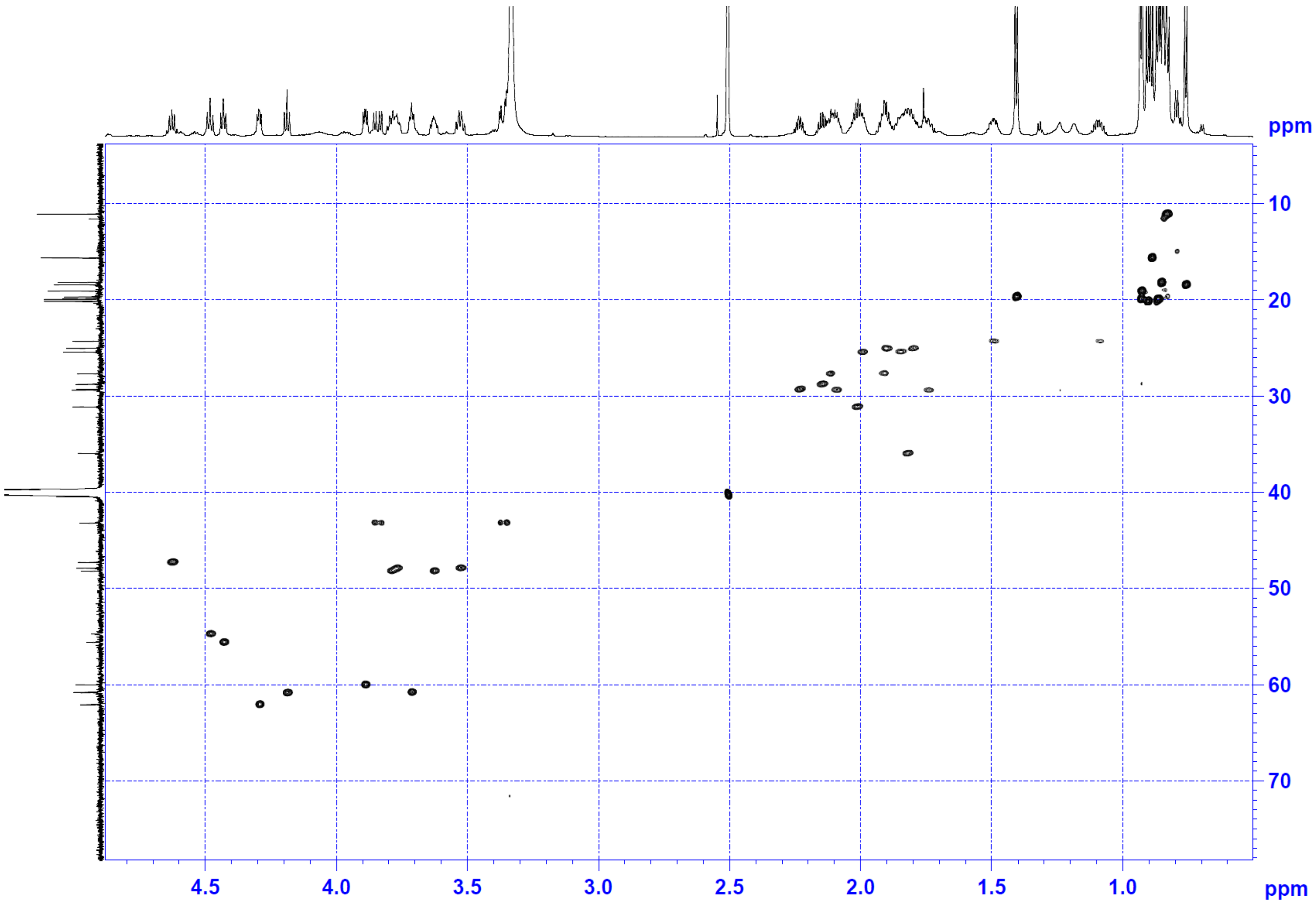

Figure S34. HSQC spectrum of compound **4**

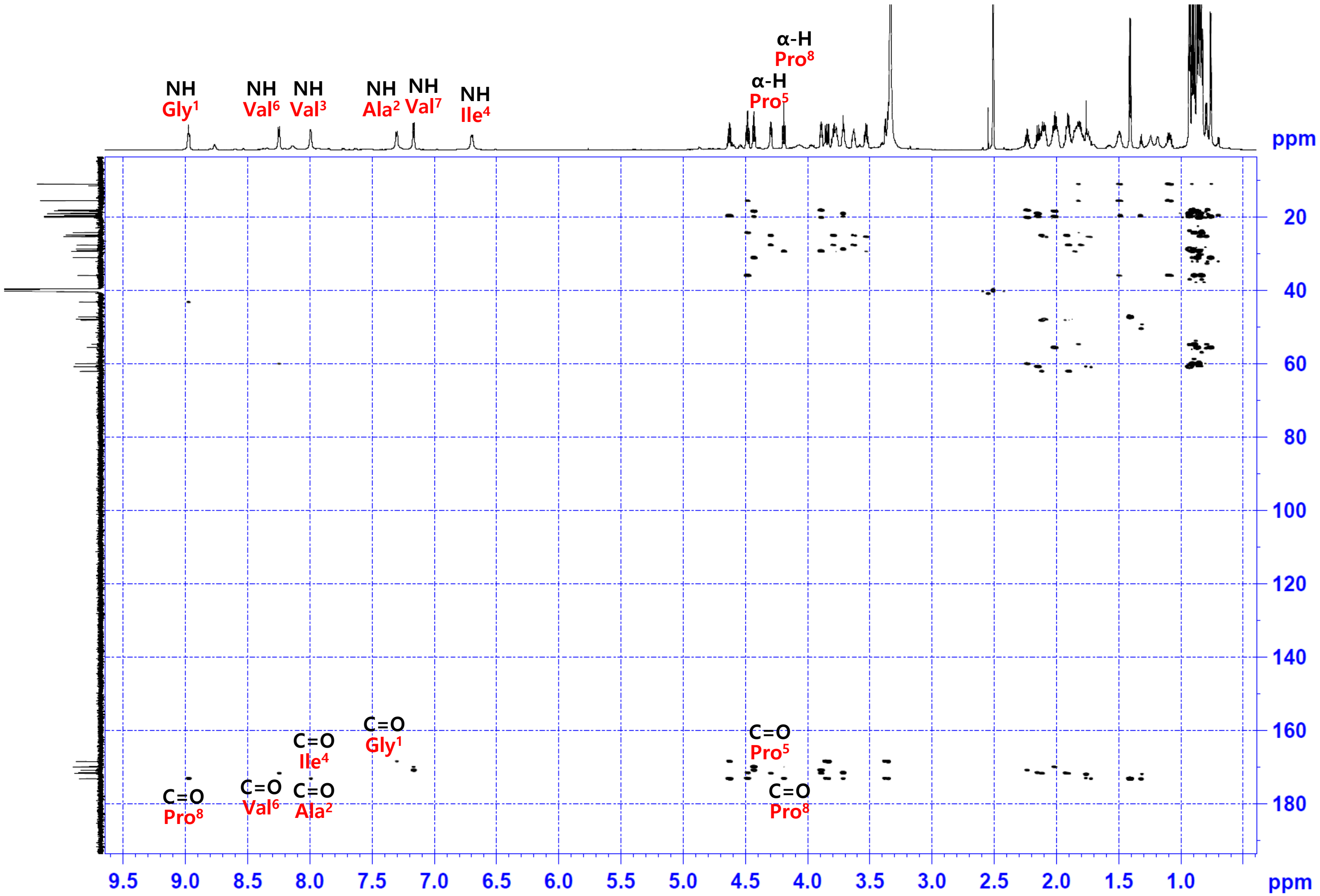

Figure S35. HMBC spectrum of compound **4**

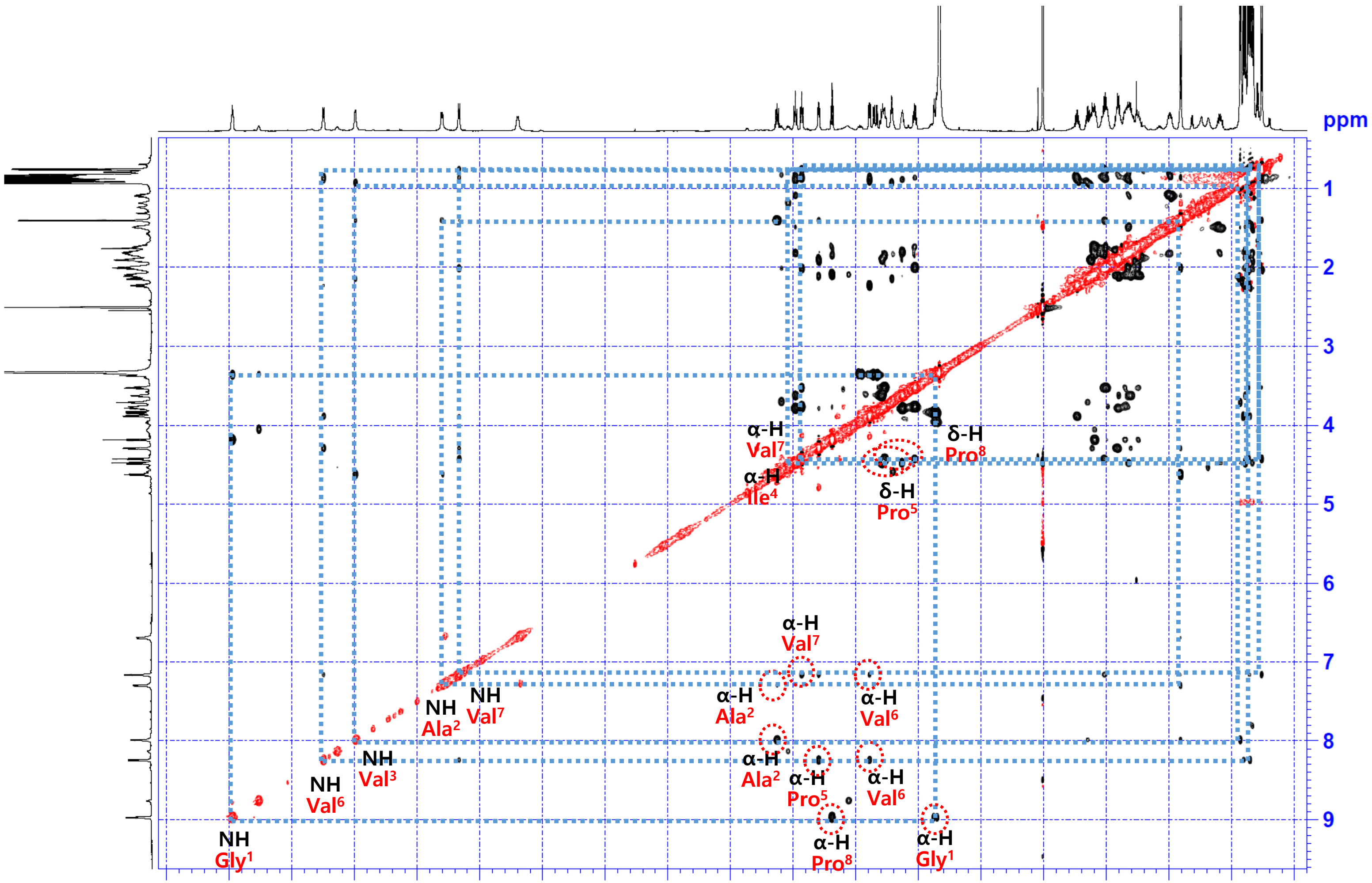

Figure S36. ROESY spectrum of compound **4**

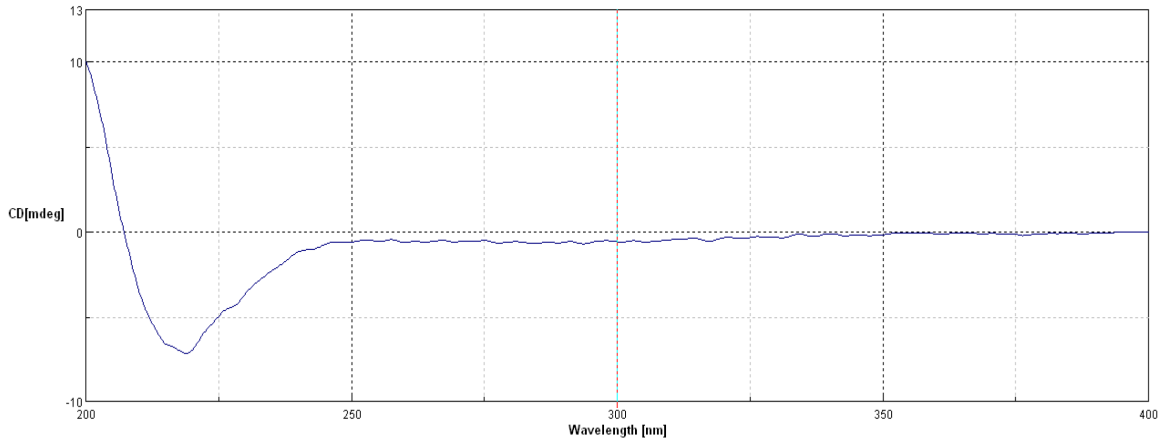

Figure S37. ECD spectrum of compound **4**

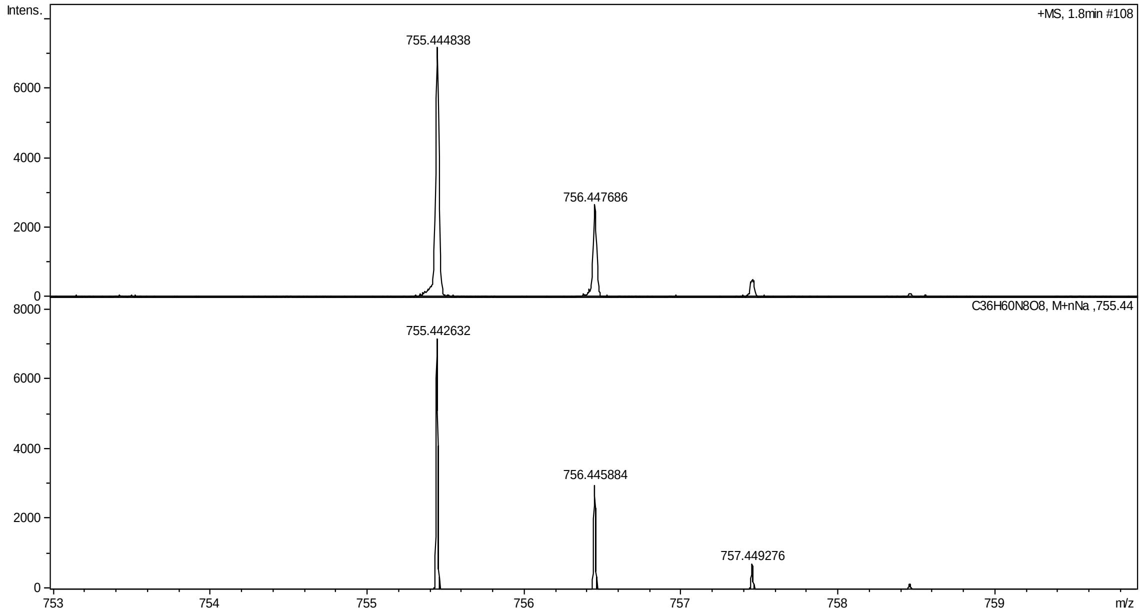

Figure S38. HRESI-MS spectrum of compound **4**

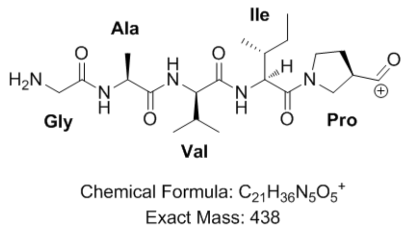

m/z	Detected ions	m/z	Detected ions
733	[M+H]$^+$	508	[Val-Ile-Pro-Val-Val]$^+$
705	[M+H-CO]$^+$	438	[Gly-Ala-Val-Ile-Pro]$^+$
662	[Val-Ile-Pro-Val-Val-Pro-Gly]$^+$	438	[Pro-Gly-Ala-Val-Ile]$^+$
634	[Ile-Pro-Val-Val-Pro-Gly-Ala]$^+$	409	[Val-Ile-Pro-Val]$^+$
634	[Val-Pro-Gly-Ala-Val-Ile-Pro]$^+$	409	[Ile-Pro-Val-Val]$^+$
634	[Pro-Gly-Ala-Val-Ile-Pro-Val]$^+$	325	[Val-Pro-Gly-Ala]$^+$
620	[Pro-Val-Val-Pro-Gly-Ala-Val]$^+$	325	[Pro-Gly-Ala-Val]$^+$
592	[(Pro-Val-Val-Pro-Gly-Ala-Val)-CO]$^+$	325	[(Val-Val-Pro-Gly)-CO]$^+$
551	[(Ala-Val-Ile-Pro-Val-Val)-CO]$^+$	313	[(Gly-Ala-Val-Ile)-CO]$^+$
537	[Gly-Ala-Val-Ile-Pro-Val]$^+$	226	[Pro-Gly-Ala]$^+$
537	[Val-Pro-Gly-Ala-Val-Ile]$^+$	226	[(Val-Pro-Gly)-CO]$^+$
521	[Pro-Val-Val-Pro-Gly-Ala]$^+$		

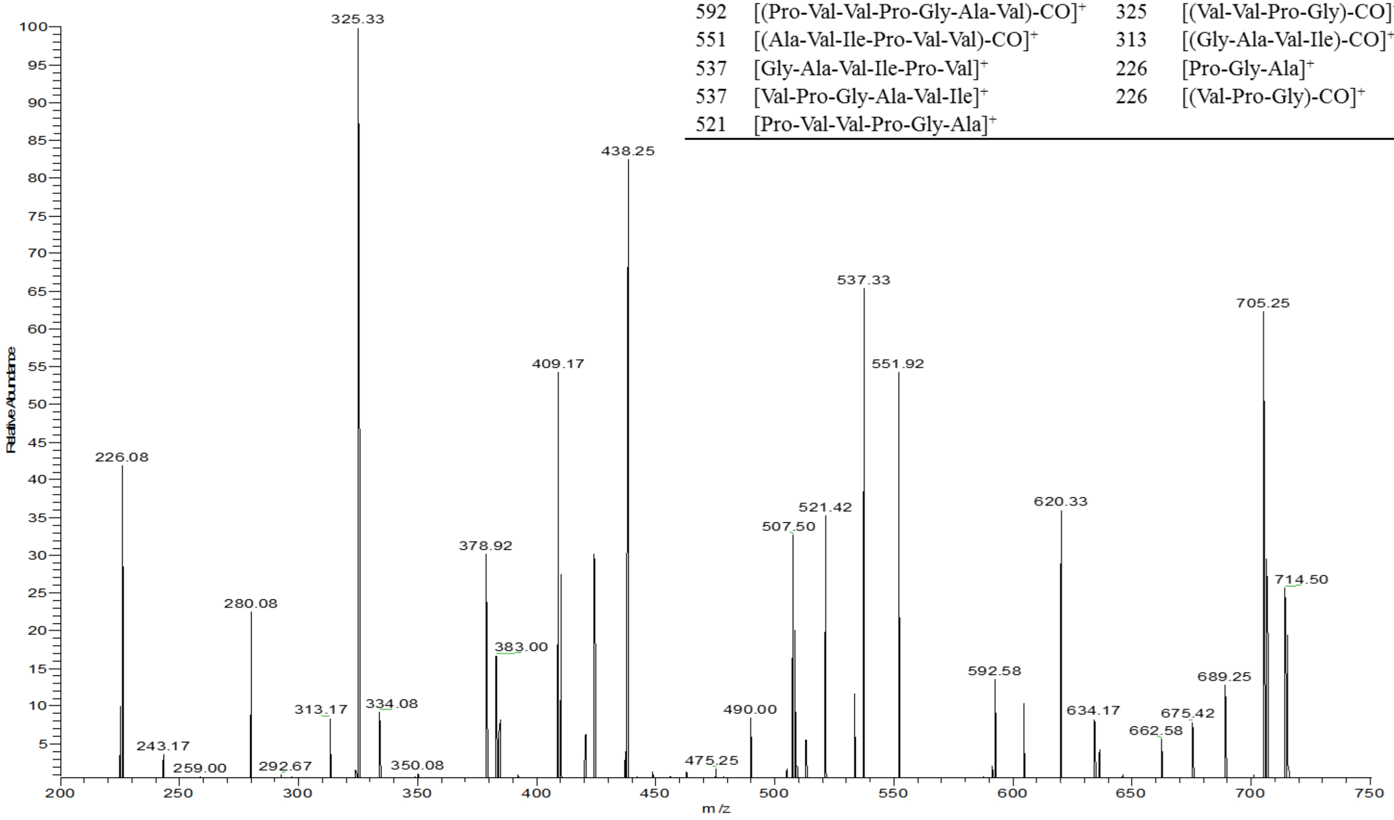

Figure S39. ESI-MS/MS spectrum of compound **4**

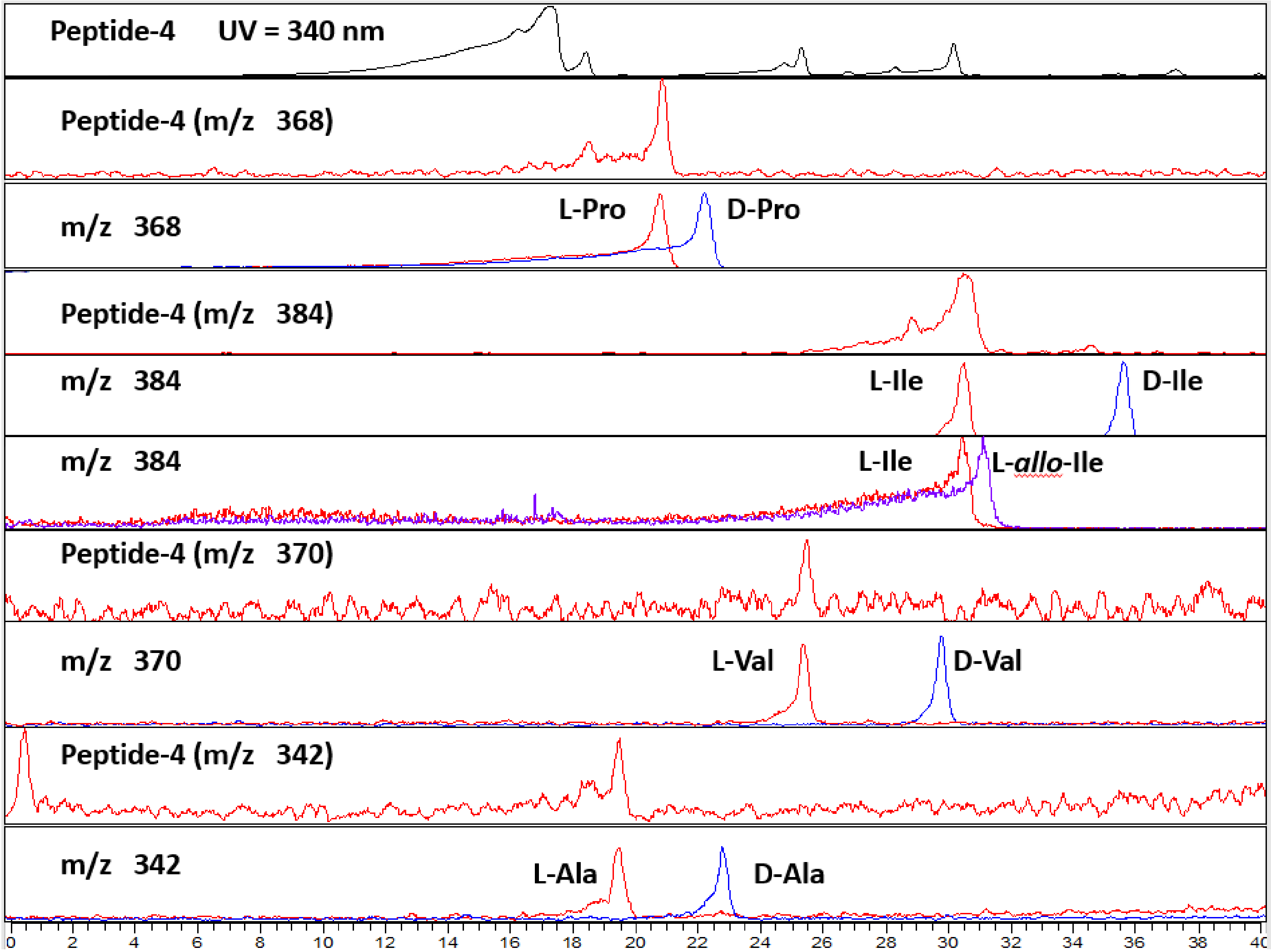

Figure S40. Marfey's analysis of compound **4**

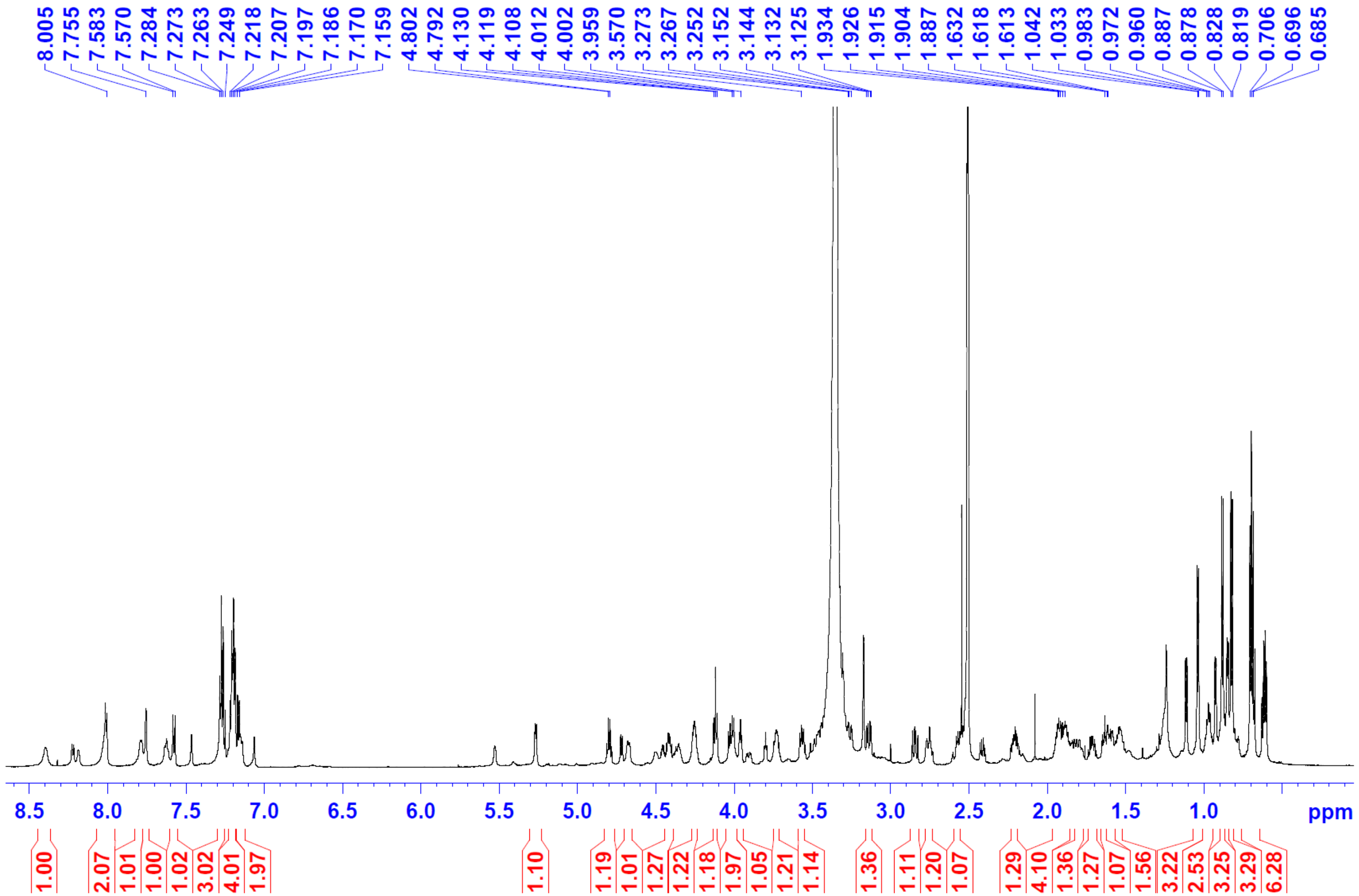

Figure S41. ^{1}H NMR spectrum of compound **5** (DMSO-d_6, 700 MHz)

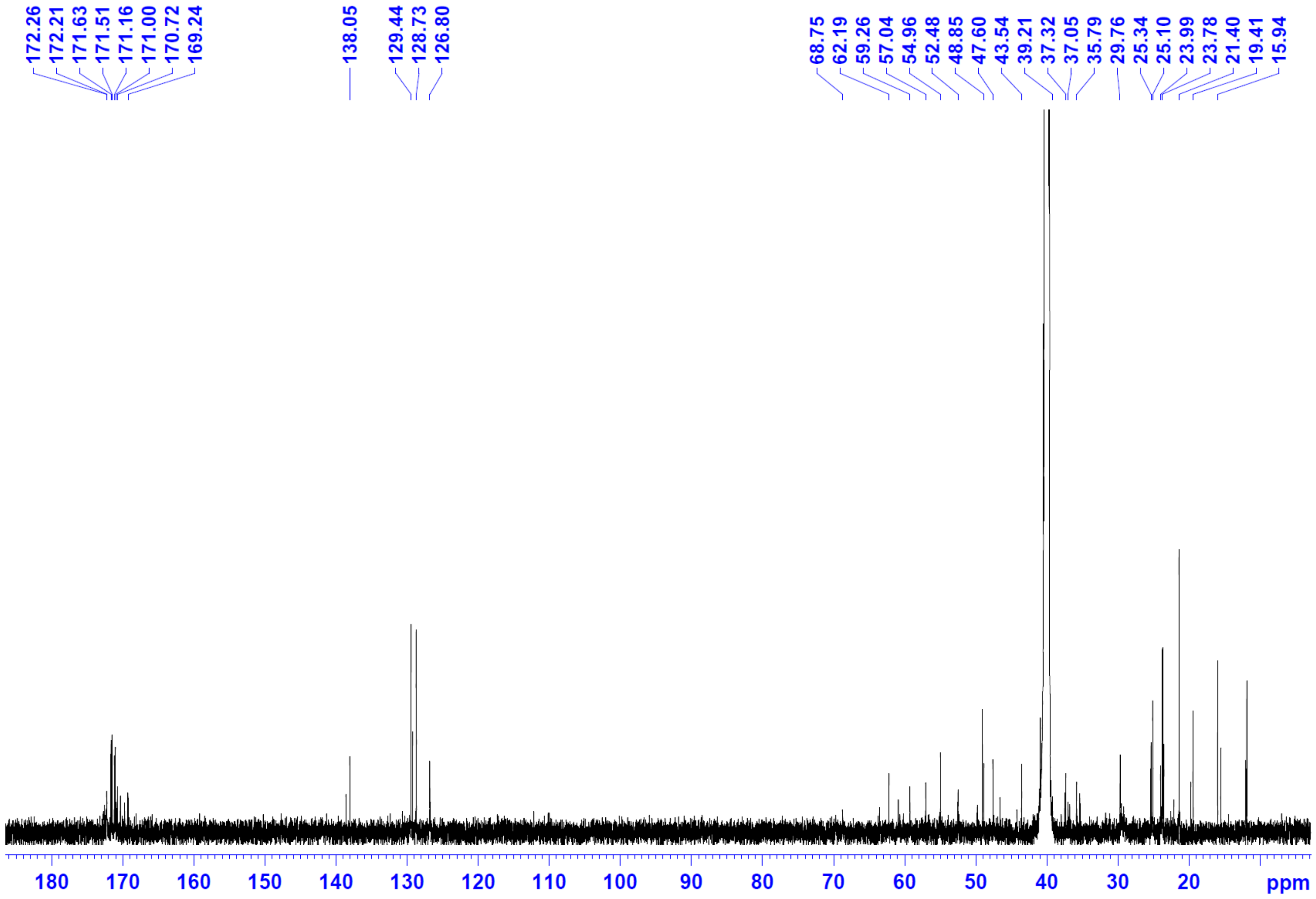

Figure S42. ^{13}C NMR spectrum of compound **5** (DMSO-d_6, 175 MHz)

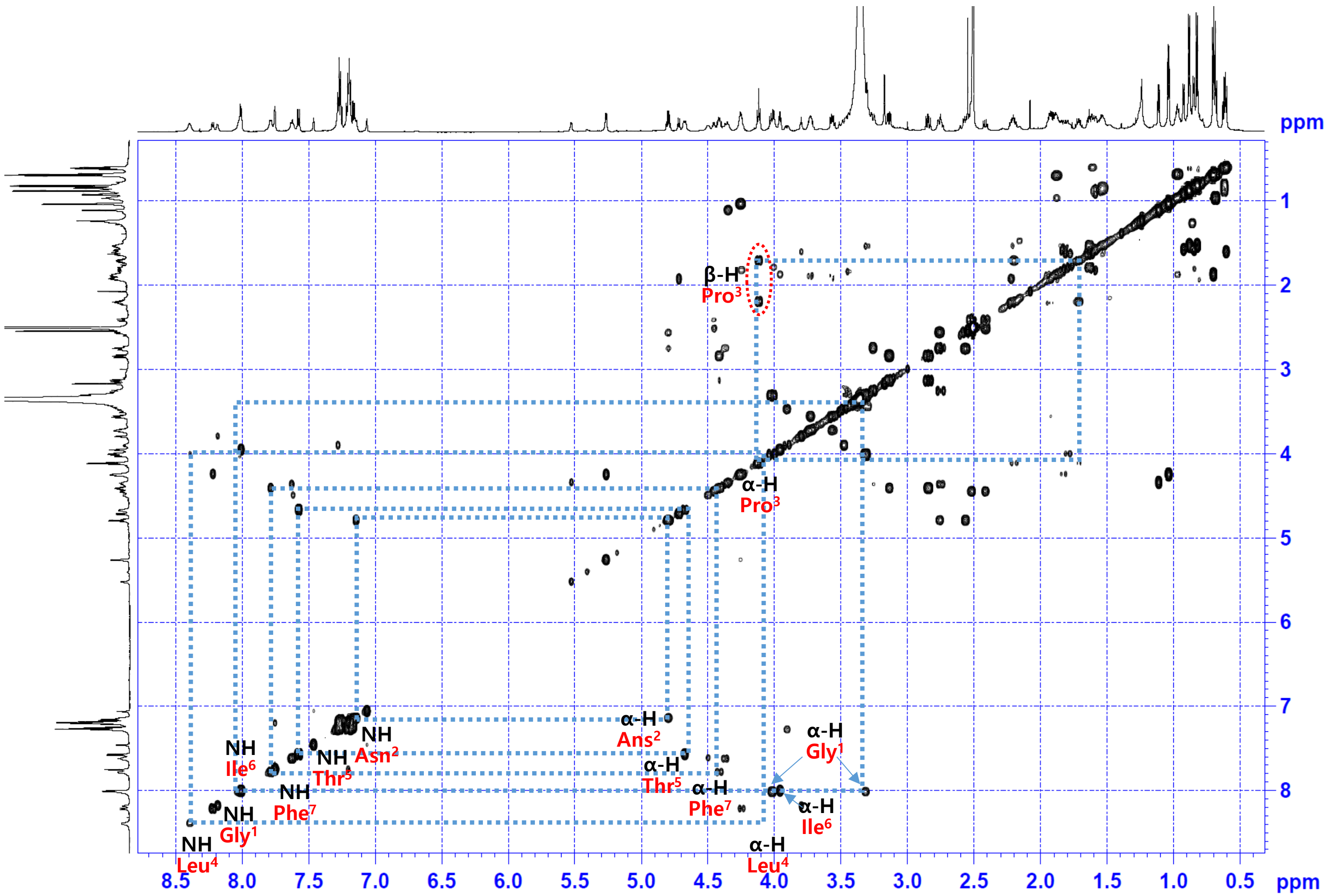

Figure S43. COSY spectrum of compound **5**

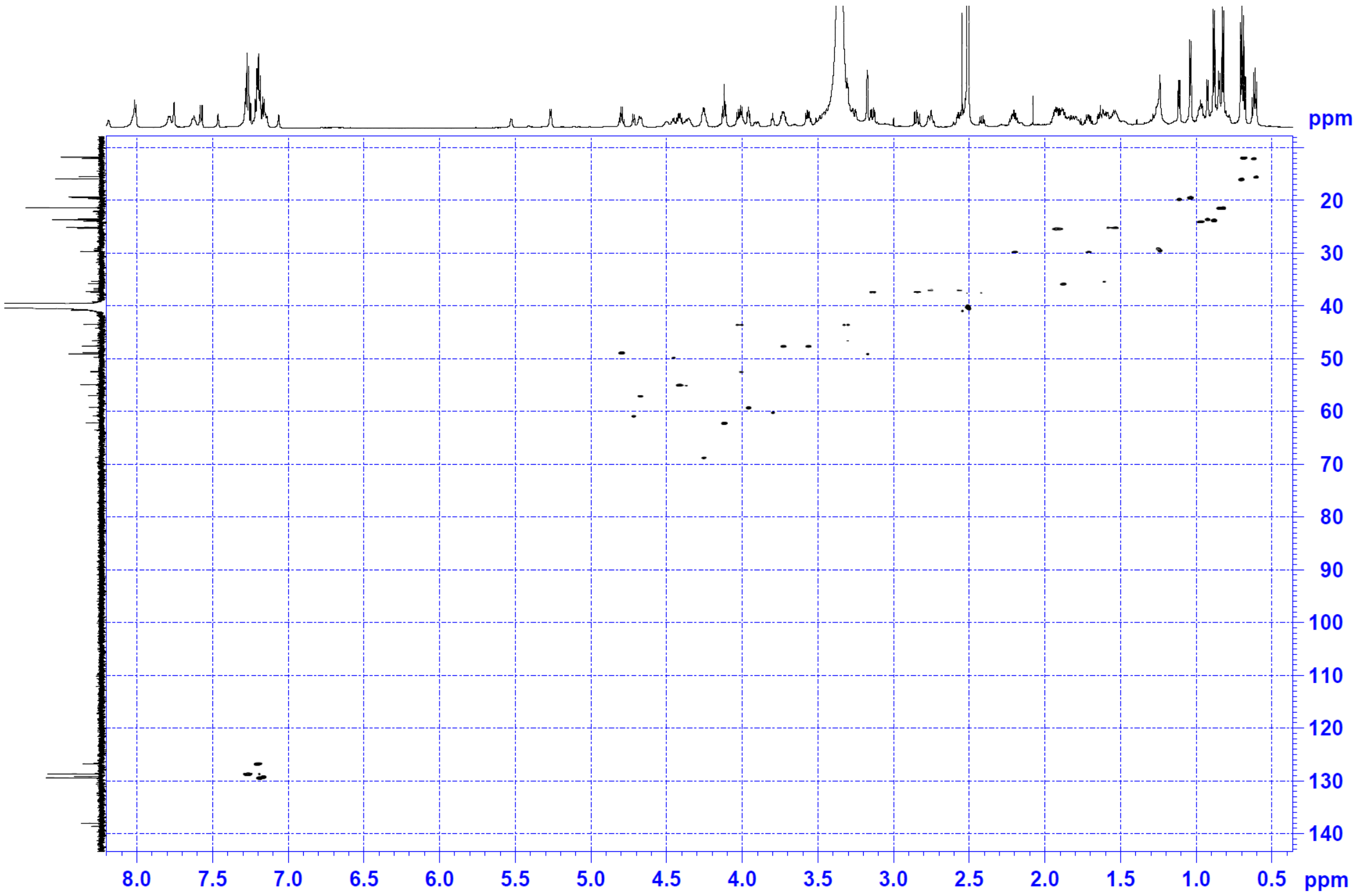

Figure S44. HSQC spectrum of compound **5**

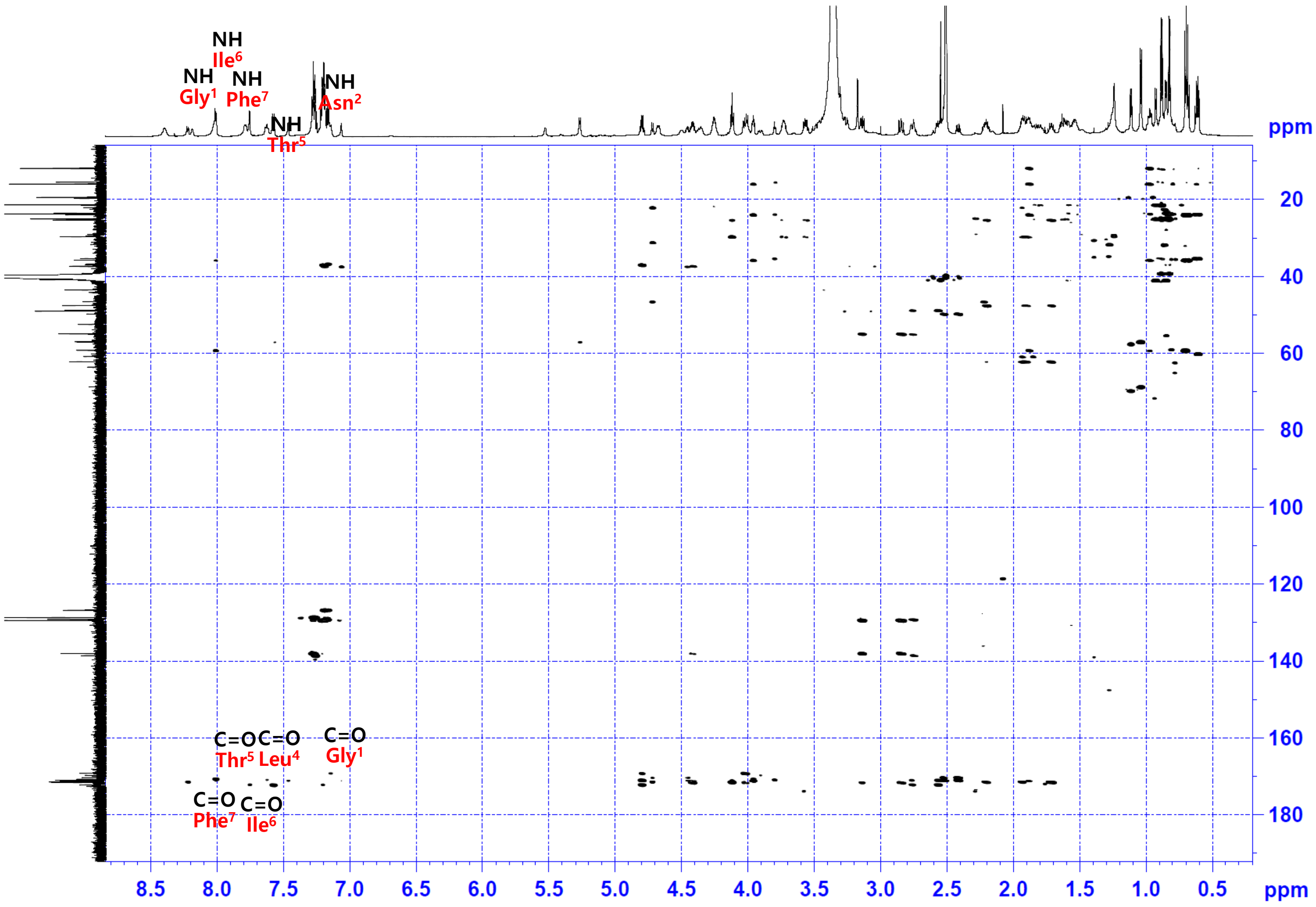

Figure S45. HMBC spectrum of compound **5**

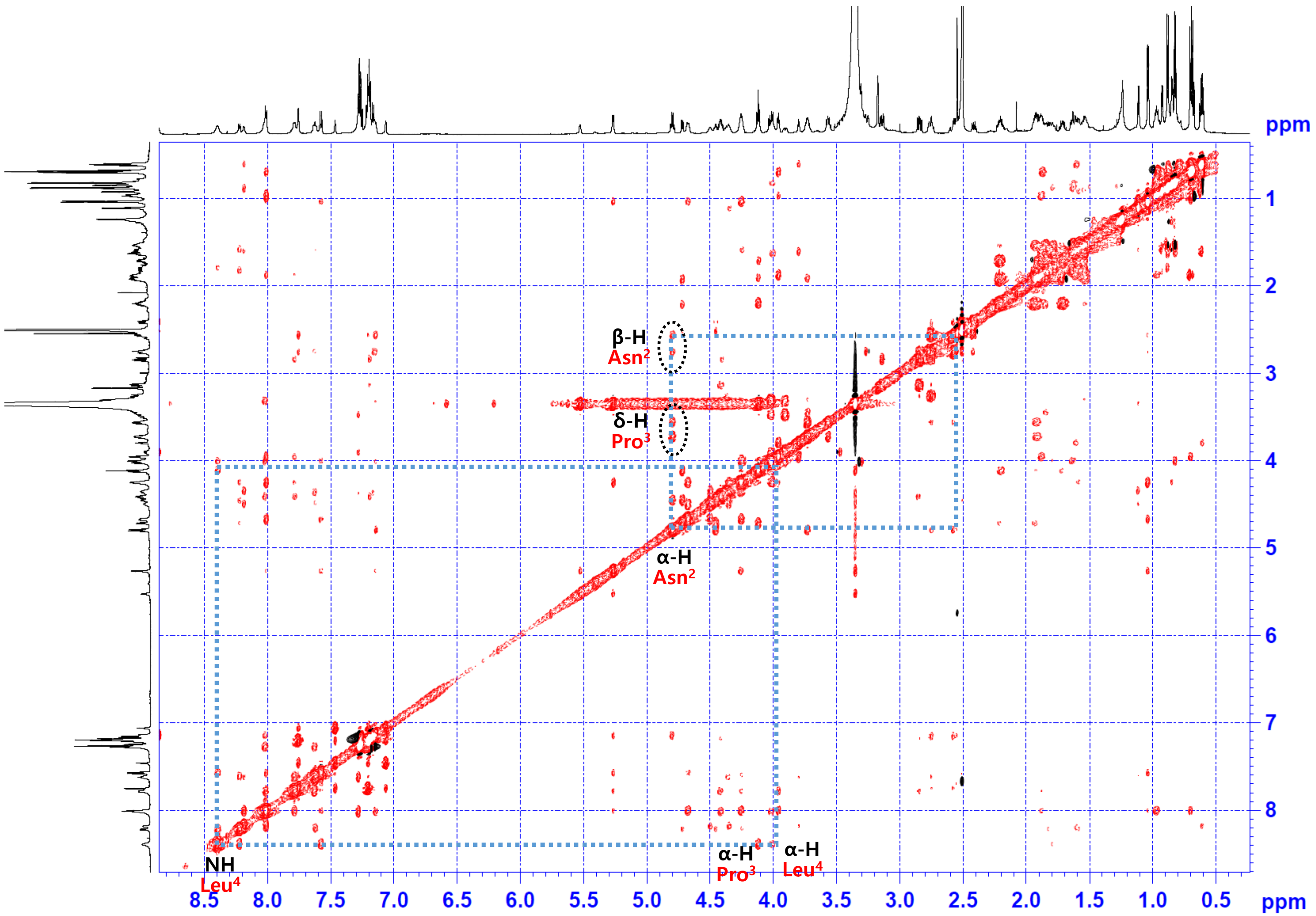

Figure S46. ROESY spectrum of compound **5**

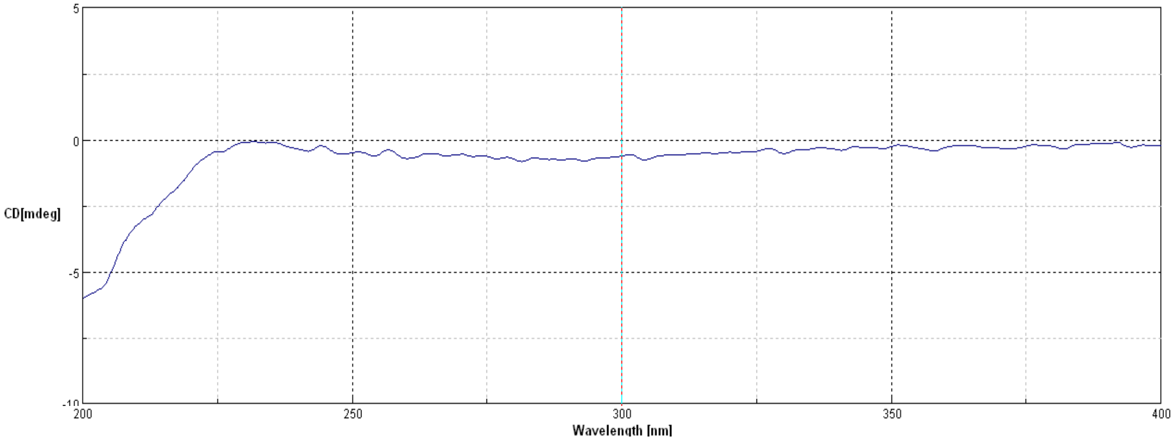

Figure S47. ECD spectrum of compound **5**

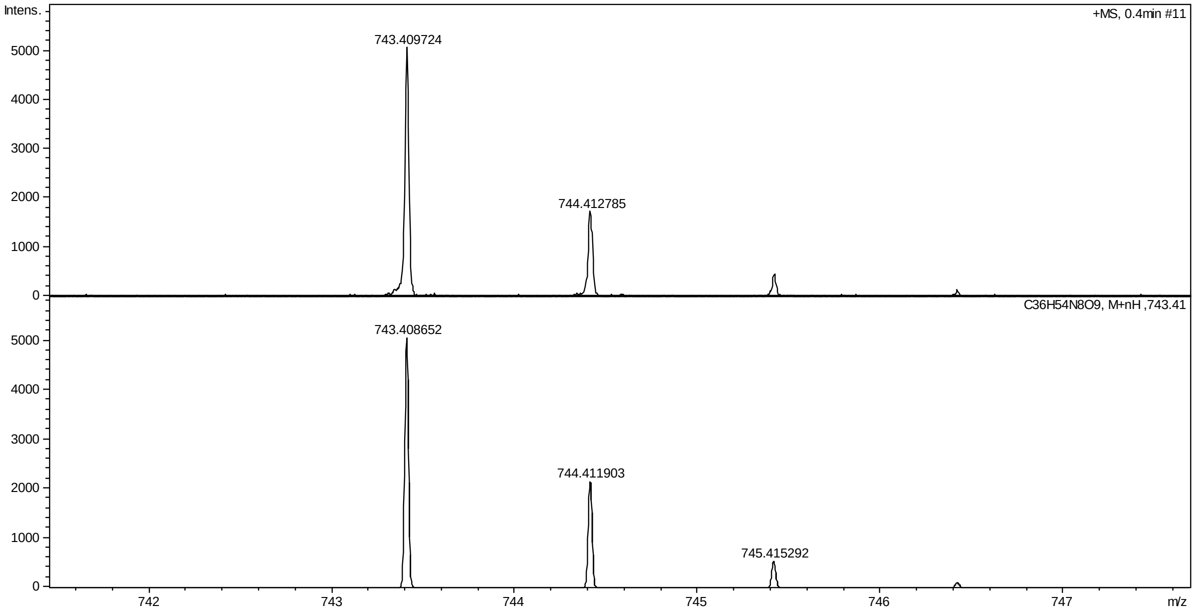

Figure S48. HRESI-MS spectrum of compound **5**

m/z	Detected ions	m/z	Detected ions
743	$[M+H]^+$	397	$[(Pro\text{-}Leu\text{-}Thr\text{-}Ile)\text{-}CO]^+$
725	$[M+H\text{-}H_2O]^+$	379	$[(Pro\text{-}Leu\text{-}Thr\text{-}Ile)\text{-}CO\text{-}H_2O]^+$
708	$[M+H\text{-}H_2O\text{-}NH_3]^+$	361	$[(Gly\text{-}Asn\text{-}Pro\text{-}Leu\text{-}Thr\text{-}Ile)\text{-}NH_3]^+$
629	$[Pro\text{-}Leu\text{-}Thr\text{-}Ile\text{-}Phe\text{-}Gly]^+$	319	$[(Thr\text{-}Ile\text{-}Phe\text{-}Gly\text{-}Asn)\text{-}NH_3]^+$
601	$[(Pro\text{-}Leu\text{-}Thr\text{-}Ile\text{-}Phe\text{-}Gly)\text{-}CO]^+$	311	$[(Leu\text{-}Thr\text{-}Ile)\text{-}NH_3]^+$
579	$[(Gly\text{-}Asn\text{-}Pro\text{-}Leu\text{-}Thr\text{-}Ile)\text{-}NH_3]^+$	294	$[(Pro\text{-}Leu\text{-}Thr)\text{-}NH_3]^+$
516	$[(Thr\text{-}Ile\text{-}Phe\text{-}Gly\text{-}Asn)\text{-}NH_3]^+$		

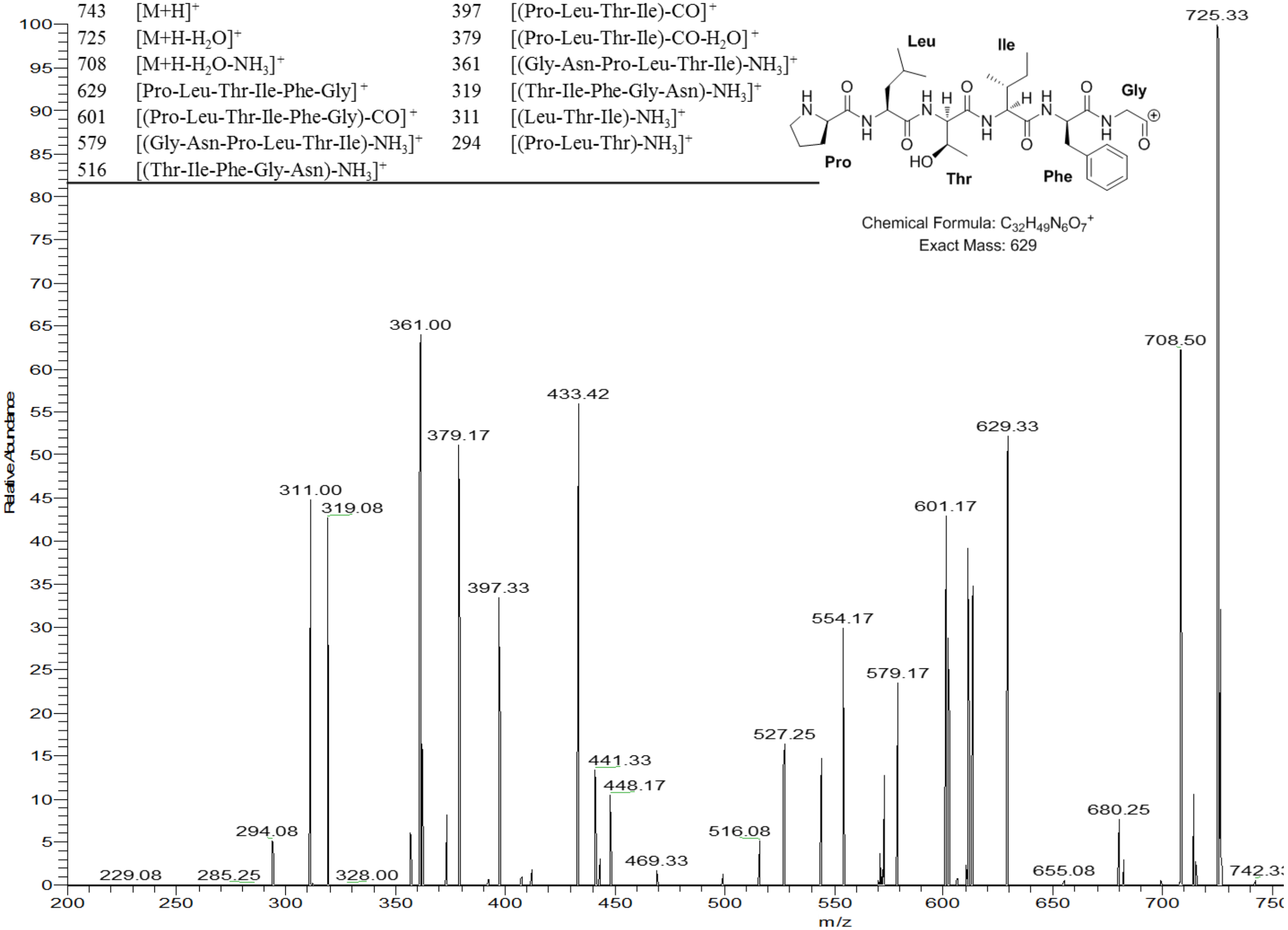

Figure S49. ESI-MS/MS spectrum of compound **5**

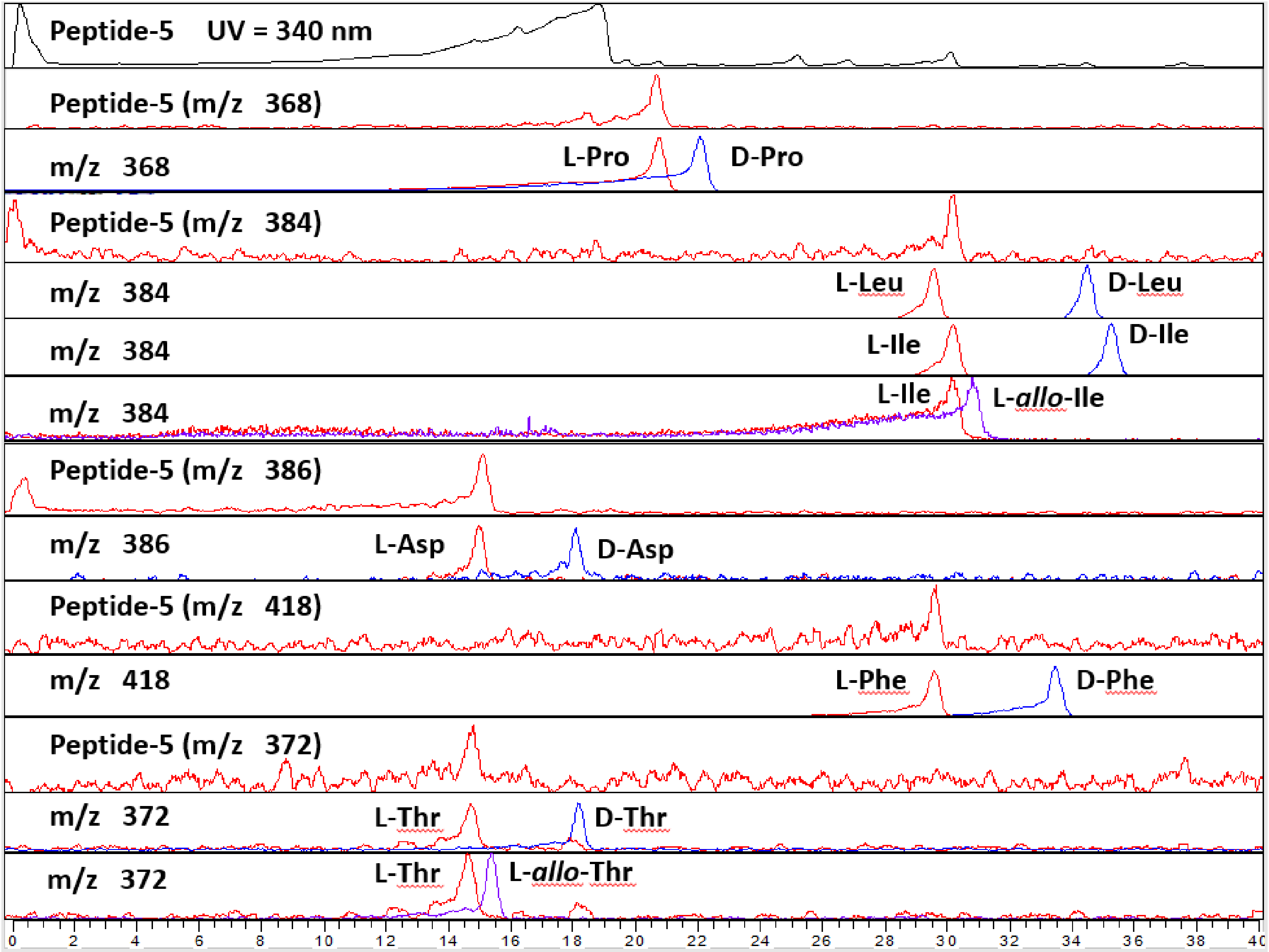

Figure S50. Marfey's analysis of compound **5**
